# Computational Screening for the Anticancer Potential of Seed-Derived Antioxidant Peptides: A Cheminformatic Approach

**DOI:** 10.3390/molecules26237396

**Published:** 2021-12-06

**Authors:** Tsun-Thai Chai, Jiun-An Koh, Clara Chia-Ci Wong, Mohamad Zulkeflee Sabri, Fai-Chu Wong

**Affiliations:** 1 Department of Chemical Science, Faculty of Science, Universiti Tunku Abdul Rahman, Kampar 31900, Malaysia; jiunan30@1utar.my (J.-A.K.); clara2000genesis@1utar.my (C.C.-C.W.); wongfc@utar.edu.my (F.-C.W.); 2 Center for Agriculture and Food Research, Universiti Tunku Abdul Rahman, Kampar 31900, Malaysia; 3 Green Chemistry and Sustainable Technology Cluster, Bioengineering Section, Universiti Kuala Lumpur Malaysian Institute of Chemical and Bioengineering Technology (UniKL MICET), Lot 1988, Bandar Vendor Taboh Naning, Alor Gajah 78000, Malaysia; mzulkeflee@unikl.edu.my

**Keywords:** anticancer, cheminformatics, in silico, Keap1, molecular docking, molecular dynamics, myeloperoxidase, NADPH oxidase, seed antioxidant peptide, xanthine oxidase

## Abstract

Some seed-derived antioxidant peptides are known to regulate cellular modulators of ROS production, including those proposed to be promising targets of anticancer therapy. Nevertheless, research in this direction is relatively slow owing to the inevitable time-consuming nature of wet-lab experimentations. To help expedite such explorations, we performed structure-based virtual screening on seed-derived antioxidant peptides in the literature for anticancer potential. The ability of the peptides to interact with myeloperoxidase, xanthine oxidase, Keap1, and p47^phox^ was examined. We generated a virtual library of 677 peptides based on a database and literature search. Screening for anticancer potential, non-toxicity, non-allergenicity, non-hemolyticity narrowed down the collection to five candidates. Molecular docking found LYSPH as the most promising in targeting myeloperoxidase, xanthine oxidase, and Keap1, whereas PSYLNTPLL was the best candidate to bind stably to key residues in p47^phox^. Stability of the four peptide-target complexes was supported by molecular dynamics simulation. LYSPH and PSYLNTPLL were predicted to have cell- and blood-brain barrier penetrating potential, although intolerant to gastrointestinal digestion. Computational alanine scanning found tyrosine residues in both peptides as crucial to stable binding to the targets. Overall, LYSPH and PSYLNTPLL are two potential anticancer peptides that deserve deeper exploration in future.

## 1. Introduction

The past decade has seen a surge in scientific interest towards the exploration of bioactive peptides for potential applications in health promotion and disease management. Bioactive peptides identified from plant food and other natural origins often range between 2 and 20 residues, although this is not a hard-and-fast definition as exceptions do exist [1,2,3]. Plant bioactive peptides, known to exhibit diverse bioactivities, such as antioxidant, antihypertensive, antimicrobial, and antitumor activities, are often purified and identified from enzymatic hydrolysates of edible plant sources and plant-based agricultural by-products. The bioactive potency of some such peptides have also been demonstrated in cellular and animal models [2,4,5]. To date, a growing body of research has shown that plant seeds are a good source of antioxidant peptides [2,5,6]. While such peptides could be developed into natural additive for food processing and nutraceuticals for health maintenance, they may also be therapeutically relevant as some could modulate cellular and/or in vivo antioxidant status [2]. Cellular redox homeostasis is connected to the initiation and/or progression of certain cancers [7,8]. Perturbation in reactive oxygen species (ROS) homeostasis resulting from unchecked ROS production is associated with carcinogenesis; scavenging of excessive ROS accumulation may prevent early neoplasia [9]. Significant reduction in the antioxidant activity of the blood serum of patients with malignant neoplasms has also been reported [10].

In the body, cellular redox status is regulated by oxidative and antioxidative enzymes, non-enzymatic antioxidants, and certain protein-protein interactions involved in regulating antioxidant gene expression. Myeloperoxidase (MPO), xanthine oxidase (XO), and nicotinamide adenine dinucleotide phosphate oxidase (NADPH oxidase) are three examples of such oxidative enzymes. MPO, an abundant heme-containing enzyme in the human neutrophils, catalyzes the reaction between hydrogen peroxide and chloride, generating hypochlorous acid, a potent oxidant. MPO-mediated oxidative burst has been linked to the initiation and progression of cancer, including tumor cell metastasis. Notably, downregulation of MPO gene expression is connected to reduction in the risk of lung, breast, and ovarian cancers [7,8]. XO is an enzyme that catalyzes the conversion of hypoxanthine to xanthine and ultimately to uric acid, producing ROS during the reaction. The importance of XO as an anticancer target is highlighted by the discovery that XO inhibitor febuxostat could repress breast cancer cell migration and the metastasis of breast cancer to the lung in animal models [11,12]. Six isoforms of NADPH oxidase are known to date. NADPH oxidase is a membrane-bound enzyme complex in phagocytes, whose primary function is the production of superoxide anion radicals. The assembly and activation of NADPH oxidase requires protein-protein interaction between the cytosolic factor p47^phox^ and transmembrane component p22^phox^ [13,14]. Due to the importance of p47^phox^-p22^phox^ interaction in NADPH oxidase activation, the interaction can be targeted in structure-based virtual screening for NADPH oxidase inhibitors [15]. Notably, enhanced NADPH oxidase expression in multiple malignant diseases supports the recognition of the NADPH oxidase family as potential targets in cancer therapies [13,16]. The Kelch-like ECH-associated protein 1 (Keap1)-nuclear factor E2-related factor 2 (Nrf2) pathway is one of the major signaling cascades involved in protecting cells against oxidative stress. The Nrf2 transcription factor can activate the transcription of cytoprotective genes implicated in protection against cancer. However, Keap1-Nrf2 protein-protein interaction could trigger Nrf2 degradation mediated by the ubiquitin–proteasome pathway. Hence, there has been strong interest among researchers to discover inhibitors of Keap1-Nrf2 protein-protein interaction. Such inhibitors may preserve or enhance the transcription-activating role of Nrf2, counteracting ROS-mediated damage in cancers [17,18].

Although a growing number of seed-derived antioxidant peptides has been documented in the literature, knowledge of their ability to modulate cellular regulators of oxidative status (i.e., MPO, XO, NADPH oxidase, and Keap1-Nrf2), which are also promising targets of anticancer therapy, is still limited. A recent report of watermelon seed-derived antioxidant peptides targeting the Keap1-Nrf2 system [19] suggests that seed-derived antioxidant peptides should be explored more intensively as potential modulators of cellular regulators of ROS balance. Thus, this in silico study was undertaken to virtually screen the numerous seed-derived antioxidant peptides in the literature for their potential as anticancer peptides that can target two oxidative enzymes (MPO and XO) and two protein-protein interactions (Keap1-Nrf2 and p47^phox^-p22^phox^). *In silico* or virtual screening is a less costly and less time-consuming strategy to screen for desirable bioactive peptides and other compounds when compared with wet-lab screening [20]. In bioactive peptide screening, this approach can benefit from various freely available peptide databases (e.g., PlantPepDB [21], and other online tools, such as AntiCP 2.0 [22] and MLCPP [23], which are anticancer peptide and cell-penetrating potential prediction servers designed from machine learning models). Moreover, different molecular modelling and simulation methods [24,25,26] may also be used to clarify the mechanisms of action between the peptides and the protein targets of interest. Although virtual screening cannot replace wet-lab experimentation, the aforementioned benefits have driven increasing popularity of in silico research on bioactive peptides [20,27]. Notably, by narrowing down a large set of candidate peptides to a small number, in silico screening can facilitate a more focused research strategy in future wet-lab experimentation; this also allows more efficient use of limited research resources [20].

The goal of this in silico study was three-fold: (a) to compile a virtual library of seed antioxidant peptides from the literature, followed by screening for non-toxic, non-allergenic, and non-hemolytic anticancer peptides; (b) to perform structure-based screening of the predicted anticancer peptides for ability to target Keap1-Nrf2, MPO, XO, and p47^phox^-p22^phox^, followed by molecular dynamics validation of peptide-target interactions; and (c) to further characterize the predicted anticancer peptides based on computational alanine mutagenesis and prediction of cell- and blood-brain barrier penetrating potential, as well as plasma and gastrointestinal (GI) stability.

## 2. Results and Discussion

**Figure 1:**
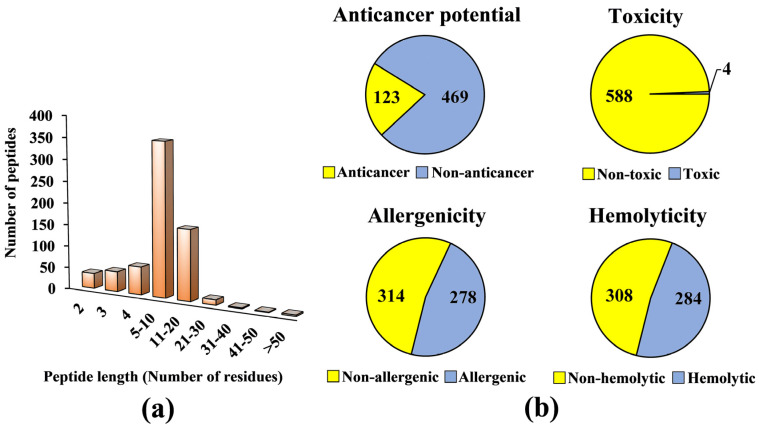
(**a**) The distribution of peptide length in the virtual library of seed-derived antioxidant peptides. (**b**) Outcome of the screening of 592 seed-derived antioxidant peptides of 4–50 residues for anticancer potential, toxicity, allergenicity, and hemolyticity. Numbers in the pie charts represent numbers of peptides with the predicted properties.

A virtual library consisting of 677 seed-derived antioxidant peptides was generated (Table S1), based on peptide sequences collected from Scopus and PlantPepDB databases, as outlined in Materials and Methods. The collection encompassed antioxidant peptides of 2–57 residues in length and 192–5338 Da in molecular mass. Seed sources in the virtual library included legumes, such as faba bean and soybean; cereals, such as wheat and rye; and seeds of plantation crop species, such as oil palm and coconut. The types of antioxidant activities reported for the seed-derived peptides included in vitro free radical scavenging activities, lipid peroxidation inhibitory activity, cellular antioxidant activity, and in vivo antioxidant activity (Table S1). Based on Figure 1a, 52% of the seed-derived antioxidant peptides contain five to ten residues. By contrast, seed-derived antioxidant peptides with more than 20 residues comprised only 0.15–0.59% of the virtual antioxidant peptide library. Among the 63 Scopus-indexed publications we examined for the preparation of the virtual library, 42 (67%) reported peptides of 5–10 residues. The prevalence of such peptide length could be accounted by many seed-derived antioxidant peptides being purified and identified from protein fractions of a relatively low molecular mass range, such as <3 kDa fractions [28,29,30].

Next, we proceeded to screening the virtual library for potential anticancer peptides. Only 592 peptides were screened since dipeptides, tripeptides, peptides with more than 50 residues, and peptides with unnatural or modified amino acid residues could not be analyzed by the AntiCP 2.0 tool. Among the 592 peptides, only 123 (21%) were predicted as anticancer (Figure 1b). We carefully examined the publications reporting the 123 peptides and found that none of the peptides had been tested for anticancer activity experimentally. The 123 predicted anticancer peptides averaged 6 residues in length and 750 Da in mass (data not shown). Safety is an important consideration in the design or discovery of anticancer peptides. A functional anticancer peptide should not exhibit toxicity, elicit immune response, and induce the lysis of erythrocytes [31,32,33]. Our screening found that at least 50% of the 592 seed-derived antioxidant peptides were predicted to be safe (i.e., non-toxic, non-allergenic, and non-hemolytic) (Figure 1b). Among the 592 peptides screened, only 0.7% (4 peptides) were predicted to be toxic (Figure 1b). An in silico study also predicted that all 253 antioxidant peptides liberated from the flaxseed proteome were non-toxic [34]. This agrees with our observation of high abundance (99%) of non-toxic peptides in our antioxidant peptide virtual library (Figure 1b). In comparison with toxicity prediction, 47–48% of our antioxidant peptide virtual library comprised allergenic and hemolytic peptides. In an in silico study of 26 antimicrobial peptides of rapeseed, 54% were predicted as non-allergenic and 46% allergenic [35]. This relative distribution of allergenicity and non-allergenicity resembles that observed in our virtual screening. Among the 308 non-hemolytic peptides (Figure 1b), the greatest proportion (41%) originated from legumes, which included soybean and chickpea (data not shown). Fourteen soybean-derived multifunctional cationic peptides were shown to have no hemolytic effect on sheep red blood cells [36]. Meanwhile, two chickpea-derived antioxidant peptides also did not cause any hemolysis in bovine red blood cells [37]. These findings support our observation of legumes being a potential source of non-hemolytic peptides.

**Figure 2:**
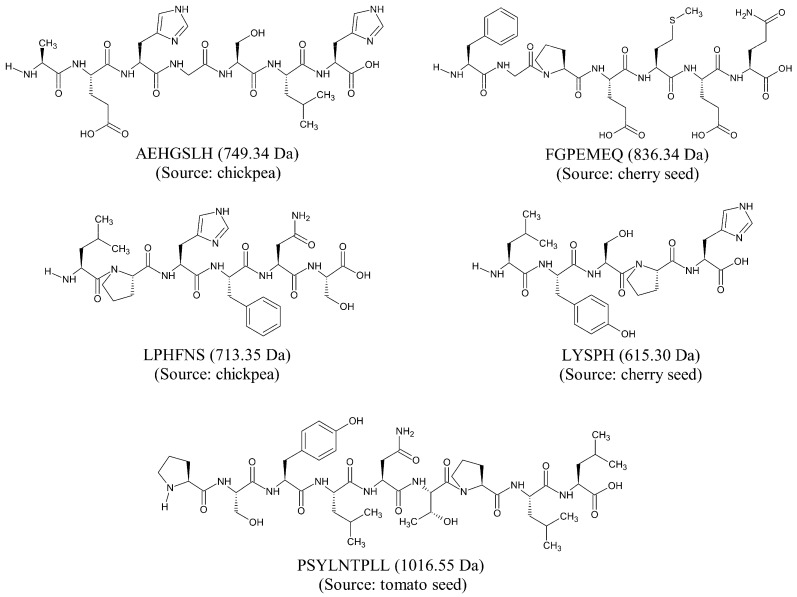
2D structures of five selected seed-derived antioxidant peptides predicted to be anticancer, non-toxic, non-allergenic, and non-hemolytic.

Based on our in silico screening, five seed-derived antioxidant peptides were predicted to be anticancer, non-toxic, non-allergenic, and non-hemolytic. The two-dimensional (2D) structures and molecular weight of the five peptides are shown in Figure 2. The five peptides, identified from chickpeas, cherry seeds, and tomato seeds, are 5–9 residues in length and 615–1016 Da in mass. The five peptides each contain at least one imidazole functional group or one aromatic ring among their amino acid side chains. Notably, LPHFNS and LYSPH each contain both an imidazole functional group and an aromatic ring in their structures. This is characteristic of many food-derived antioxidant peptides; imidazole groups and aromatic rings are associated with the ability of the peptides to scavenge free radicals by electron transfer/proton donation [38]. On the other hand, among the five peptides (Figure 2), FGPEMEQ has Phe (F) at the N-terminus, whereas Leu (L), His (H), and Phe (F) are present in four, three, and two of the peptides, respectively. The N-terminal preference for Phe and the abundance of Leu, His, and Phe are both characteristics of experimentally validated anticancer peptides [22].

**Figure 3:**
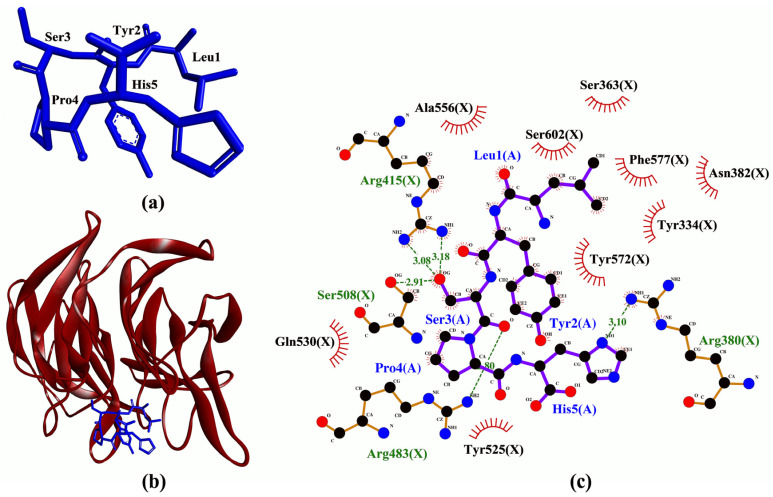
(**a**) Three-dimensional (3D) diagram of LYSPH; (**b**) 3D diagram of LYSPH-Keap1 docked model; (**c**) 2D LYSPH-Keap1 interaction diagram. In (**a**,**b**), LYSPH is displayed in a blue-stick style. In (**b**), Keap1 is displayed as red ribbon. In (**c**), green dashed lines and red spoked arcs represent hydrogen bonds and hydrophobic interactions, respectively. Residues of LYSPH are shown in purple bonds, whereas residues of Keap1 are shown in brown bonds and also represented by the red spoked arcs.

In this in silico study, to investigate whether the five predicted anticancer peptides (Figure 2) could modulate cellular targets of cancer treatments, we docked the five peptides on Keap1, MPO, XO, and p47^phox^. To the best of our knowledge, structure-based virtual screening of the five peptides on the four targets has not been reported. Molecular docking analysis found that LYSPH, a cherry seed peptide, had the strongest binding affinity to Keap1, whereas PSYLNTPLL, a tomato seed peptide, had the weakest (Table 1). LYSPH, LPHFNS, and AEHGSLH also had binding affinity values more negative than that of ETGE (−7.1 kcal/mol) (data not shown). ETGE is the key motif of the co-crystalized 16-mer Nrf2 peptide that is involved in Keap1-Nrf2 interaction [39]. Thus, LYSPH, LPHFNS, and AEHGSLH could form similarly stable or more stable binding to Keap1 when compared with Nrf2. Furthermore, all five peptides could bind to the key residues of Keap1 that are required for stable Keap1-Nrf2 complex formation, mostly accomplished via hydrogen bonds and hydrophobic interactions (Table 1). Two tripeptides (DKK and DDW) that could bind to the key residues of Keap1 have been shown experimentally to inhibit Keap1-Nrf2 interaction in vitro [40]. DKK, which possessed a stronger activity than DDW, was reported to bind to key residues Arg380 and Asn382 [40]. Similar to DKK, all five seed-derived peptides in Table 1 were predicted to bind to Arg380 and Asn382. Thus, our binding affinity and intermolecular interaction results suggest that LYSPH, LPHFNS, and AEHGSLH may serve as potential inhibitors of Keap1-Nrf2 interaction. Specifically, at the molecular level, LYSPH was predicted to bind with the same Keap1 residues as did ETGE, namely, Arg380, Arg415, Arg483, and Ser508 [39]. This observation, in addition to LYSPH having the most negative binding affinity to Keap1 among the five peptides, suggests that the peptide is the most promising for targeting Keap1-Nrf2 interaction. A graphical representation of a LYSPH-Keap1 docked model and the intermolecular interactions between LYSPH and Keap1 is shown in Figure 3.

**Figure 4:**
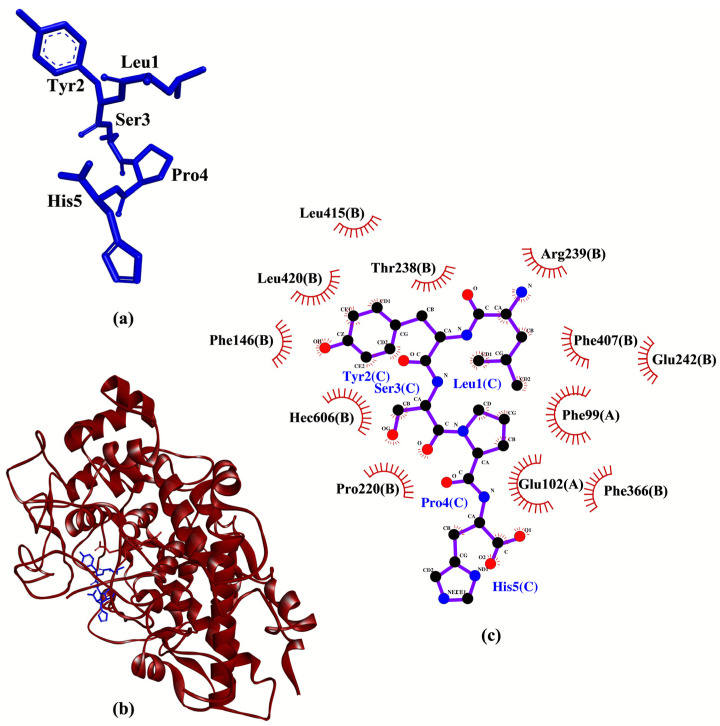
(**a**) 3D diagram of LYSPH; (**b**) 3D diagram of LYSPH-MPO docked model; (**c**) 2D LYSPH-MPO interaction diagram. In (**a**,**b**), LYSPH is displayed in a blue-stick style. In (**b**), MPO is displayed as red ribbon. In (**c**), red spoked arcs represent hydrophobic interactions. Residues of LYSPH are shown in purple bonds. Residues of MPO are represented by red spoked arcs.

LYSPH showed the strongest binding to MPO, whereas PSYLNTPLL the weakest (Table 2), similar to our observations when the five peptides were docked to Keap1 (Table 1). None of the peptides showed better binding affinity to MPO than did 7-benzyl-1H-[1–3]triazolo[4,5-b]pyridin-5-amine (7GD)(−7.1 kcal/mol) (data not shown), a co-crystalized inhibitor of MPO [41]. However, all five peptides could form hydrophobic interactions with one of the catalytic residues (Arg239) of MPO. Besides, all five peptides could interact with the heme group (Hec606) through hydrophobic interactions (Table 2); the heme group is a cofactor in the active site of MPO [42]. Based on the interactions with both catalytic residue Arg239 and the heme group of MPO, all the five peptides are potential MPO inhibitors. Supporting this possibility is the finding that two experimentally-validated anti-MPO peptides (TDY and FAPQY) could also bind to Arg239 and the heme group of MPO [43]. Analysis of intermolecular interactions revealed that LYSPH could form hydrophobic interactions with Phe99, Thr238, Arg239, Glu242, Phe366, Phe407, and Hec606 of MPO (Table 2), resembling to the binding pattern of 7GD [41]. Hence, LYSPH is the most promising MPO inhibitor among the five peptides as it showed the best binding affinity to MPO and could interact with MPO similarly as 7GD. A graphical representation of LYSPH-MPO docked model and the intermolecular interactions between LYSPH and MPO is depicted in Figure 4.

**Figure 5:**
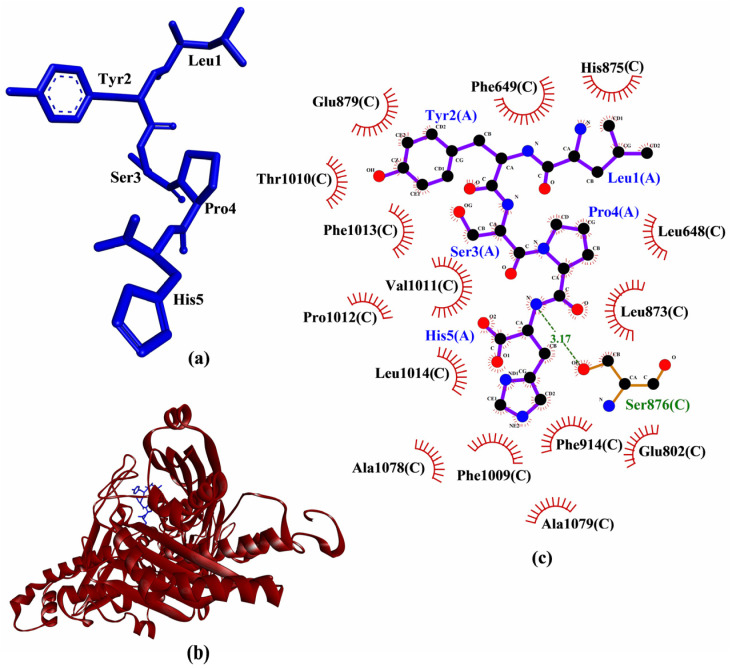
(**a**) 3D diagram of LYSPH; (**b**) 3D diagram of LYSPH-XO docked model; (**c**) 2D LYSPH-XO interaction diagram. In (**a**,**b**), LYSPH is displayed in a blue-stick style. In (**b**), XO is displayed as red ribbon. In (**c**), green dashed line and red spoked arcs represent hydrogen bond and hydrophobic interactions, respectively. Residues of LYSPH are shown in purple bonds, whereas residues of XO are shown in brown bonds and also represented by the red spoked arcs.

Comparison of binding affinities found LYSPH (−6.2 kcal/mol) to have the most stable binding to XO among the five peptides analyzed (Table 3). Nevertheless, all of the five peptides had less negative binding affinities to XO than quercetin (−8.2 kcal/mol) (data not shown), a co-crystalized inhibitor of XO [12]. This implies that none of the peptides could bind more stably to XO when compared with quercetin. On the other hand, analysis of intermolecular interactions showed that all five peptides could bind to at least nine of the XO residues known to bind to quercetin, mainly through hydrophobic interactions. Each of the peptides could also bind to at least one catalytic residue (Glu802 or Arg880) of XO [12] through hydrophobic interactions. FGPEMEQ and PSYLNTPLL could also hydrogen bond to Glu802 and Arg880, respectively. However, despite additional interactions with Glu802 and Arg880, FGPEMEQ-XO interaction was predicted to be slightly less favorable than LYSPH-XO interaction based on comparison of their binding affinity values. Meanwhile, PSYLNTPLL-XO interaction was likely non-favorable or non-spontaneous considering the positive value predicted for its binding affinity (Table 3). Previous studies found that experimentally-proven XO-inhibitory peptides, KGFP [45] and EEAK [46] could both bind to the catalytic residue Glu802. Thus, the aforementioned binding patterns of the five seed peptides to XO, particularly their binding to XO catalytic residues, suggest that the peptides are potential XO inhibitors. LYSPH could be the most promising XO inhibitor among the five peptides considering its strongest binding affinity and its binding to XO residues that known XO inhibitors bind to (Table 3). A graphical representation of a LYSPH-XO docked model and the intermolecular interactions between LYSPH and XO is shown in Figure 5.

In the molecular docking to p47^phox^, tomato seed-derived PSYLNTPLL had the best docking score, whereas the cherry seed-derived FGPEMEQ had the worst (Table 4). All peptides had docking score less negative than that of proline-rich peptide derived from p22^phox^ (−309.862) (data not shown). Thus, none of the five peptides could bind more stably to p47^phox^ than the p22^phox^-derived peptide. Nevertheless, the potential of the five peptides as inhibitors of p47^phox^-p22^phox^ interaction could not be completely ruled out solely based on this. Supporting this proposition is the observation that four peptides (RRSSIRNAHSIHQRSRKRLS, ISNSESGPRGVHFIFNKENF, RSRKRLSQDAYRRNSVRFLQQR, and AGGPPGGPQVNPIPVTDEVV) that were experimentally demonstrated to inhibit p47^phox^-p22^phox^ interaction [47,48] were also predicted to bind less strongly to p47^phox^ than was p22^phox^ (Table S4). In short, a peptide predicted to bind less strongly to p47^phox^ than p22^phox^ may still inhibit p47^phox^-p22^phox^ interaction.

**Figure 6:**
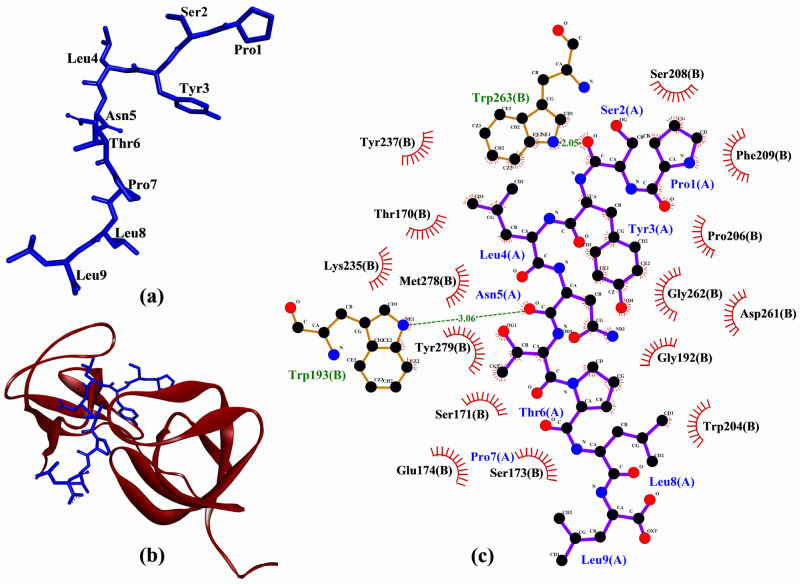
(**a**) 3D diagram of PSYLNTPLL; (**b**) 3D diagram of PSYLNTPLL-p47^phox^ docked model; (**c**) 2D PSYLNTPLL-p47^phox^ interaction diagram. In (**a**,**b**), PSYLNTPLL is displayed in a blue-stick style. In (**b**), p47^phox^ is displayed as red ribbon. In (**c**), green dashed lines and red spoked arcs represent hydrogen bonds and hydrophobic interactions, respectively. Residues of PSYLNTPLL are shown in purple bonds, whereas residues of p47^phox^ are shown in brown bonds and also represented by the red spoked arcs.

As shown in Table 4, each of the peptides could bind to at least six of the 17 p47^phox^ residues known to bind to the p22^phox^-derived peptide. However, only PSYLNTPLL, LYSPH, and FGPEMEQ could interact with Phe209, a key residue of p47^phox^ which accounts for high-affinity binding between p47^phox^ and p22^phox^ (Table 4). Furthermore, PSYLNTPLL could bind to p47^phox^ in a similar manner as the co-crystalized p22^phox^-derived peptide, by binding to Trp193, Trp204, Pro206, Phe209, Tyr237, Trp263, Met278, and Tyr279. Hence, PSYLNTPLL is the most promising among the five peptides to target p47^phox^-p22^phox^ interaction considering its docking score and pattern of binding to p47^phox^. A graphical representation of PSYLNTPLL-p47^phox^ docked model and the intermolecular interactions between PSYLNTPLL and p47^phox^ is shown in Figure 6.

Based on binding affinities and similarity of binding patterns to those of co-crystalized inhibitors/ligands and reported peptide-based inhibitors, our analyses found LYSPH and PSYLNTPLL to have the greatest potential as modulators of the four targets of cancer treatments that we investigated. Specifically, LYSPH may be a multi-target peptide which could bind to, thus inhibiting the activity of MPO and XO, as well as interrupting Keap1-Nrf2 complex formation. On the other hand, PSYLNTPLL is the most promising peptide that could bind to p47^phox^, thus precluding p47^phox^-p22^phox^ interaction and the subsequent activation of NADPH oxidase. Inhibition of the four targets could potentially dampen ROS overproduction which is associated with the initiation and/or progression of certain cancers [11,49,50,51,52]. To our knowledge, the Keap1-, MPO-, XO-, and p47^phox^-binding activity of the two peptides have not been previously reported. Considering the in silico evidence presented here, future investigations of the effectiveness of LYSPH and PSYLNTPLL in modulating the four targets, thus repressing ROS production and even cancer initiation and/or progression are warranted.

The five anticancer peptides predicted from our virtual library were also screened for cell-penetrating potential, blood-brain barrier penetrating potential, plasma half-life, and tolerance to in silico GI digestion, which can shed light on their potential as anticancer agents. Among the five peptides, LYSPH and PSYLNTPLL had the top two best plasma half-life (Table 5). The two peptides predictably had cell- and blood-brain barrier penetrating potential, although both were susceptible to GI digestion. The predicted cell-penetrating potential of the two peptides supports their potential in entering body cells and binding to the four intracellular targets: MPO, XO, Keap1, and p47^phox^, modulating the functions of the four proteins. The predicted ability of LYSPH and PSYLNTPLL to cross the blood-brain barrier may also facilitate their development as brain-tumor targeting peptides. Plasma half-life and tolerance to in silico GI digestion are related to the bioavailability of a peptide. Our results suggests that the two peptides were similar in their level of susceptibility to plasma peptidases, thus not differing much in their stability during systemic circulation. When compared with other natural anticancer peptides, such as KENPVLSLVNGMF identified from the giant barrel sponge *Xestospongia testudinaria* (half-life of 3.2 h in human serum in vitro) [53], the half-life of LYSPH and PSYLNTPLL was relatively short (about 14 min). Meanwhile, LYSPH and PSYLNTPLL were similarly susceptible to degradation by GI proteases. So, poor stability in blood and susceptibility to GI digestion is a key potential weakness of the two peptides, despite their ability to target MPO, XO, Keap1, and p47^phox^, as well as predictably having cell- and blood-brain barrier penetrating potential. The stability issue may limit the potential effectiveness of LYSPH and PSYLNTPLL as anticancer agents in the body, whether introduced into the body through oral or non-oral routes. To enhance the in vivo bioavailability of LYSPH and PSYLNTPLL, structural modifications that could improve their resistance to plasma and GI peptidases, such as cyclization of peptides [54] could be considered in future research. Moreover, the application of innovative technology such as mucoadhesive nanoparticles [55] may also be explored for oral delivery of the peptides with reduced risk of GI degradation and enhanced bioavailability.

Based on computational alanine scanning, Tyr played the most significant role in the binding and stabilizing of peptide-protein complexes for Keap1, MPO, XO, and p47^phox^. This can be observed from the drastically elevated ∆∆G values after the substitution of Tyr to Ala in both LYSPH and PSYLNTPLL (Table 6). This suggests that the hydrophobic interactions between Tyr and the residues of Keap1 (Tyr572), of XO (Glu879, Thr1010, Phe1013), of MPO (Phe99, Glu102, Phe146, Leu415, Leu420) and of p47^phox^ (Gly192, Asp261, Gly262, Met278) (Figure 3, Figure 4, Figure 5 and Figure 6) are critical to the formation of stable peptide-protein complexes. In line with our findings, Wu and co-workers [56] found that the only Tyr-containing peptide in their study had the highest XO inhibitory activity; Tyr in the peptide also interacted hydrophobically with Phe1013 of XO. On the other hand, Ala substitution of His in LYSPH also led to the second largest increase in ∆∆G by 14.2670 kJ/mol (Table 6) when the LYSPH-XO complex was analyzed. By contrast, Ala substitution of His in LYSPH led to only a minor increase in ∆∆G of the LYSPH-Keap1 and LYSPH-MPO complexes. A possible explanation is that the His residue of LYSPH could bind to more key residues in XO (Glu802, Phe914, Phe1009, and Leu1014) (Figure 5c). By contrast, the His residue of LYSPH interacted with only one key residue (Arg380) in Keap1 (Figure 3c) and with none in MPO (Figure 4c). Our analysis suggests that future research that considers re-designing LYSPH and PSYLNTPLL for enhanced interactions with Keap1, MPO, and p47^phox^ should avoid replacing or removing the Tyr residue. For stable binding to XO, the Tyr and His residues of LYSPH both should not be replaced or removed.

Molecular dynamics (MD) is a simulation technique which applied to derive the statements about the structural, dynamical, and thermodynamic properties of a molecular system [57]. The approach is able to observe minor conformational changes corresponds to the residue side chains which affect the binding site of a protein and ligand complementarity [57]. In the current study MD was applied to observe the dynamic level stability of each peptide ligand against the targeted proteins, as the peptides can functions either as the receptor inhibitors [58,59] or as the mediator such as the peptide mediated interactions in cell signaling [60].

**Figure 7:**
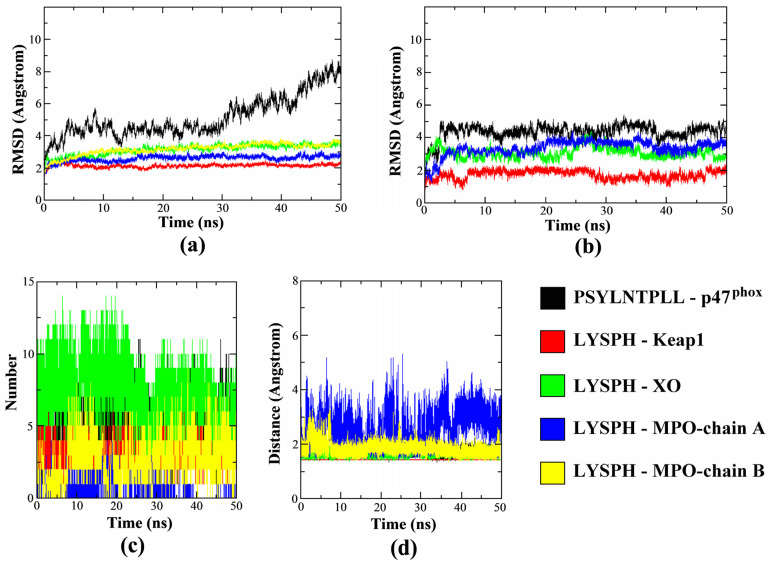
(**a**) all-atom RMSD value for proteins; (**b**) all-atom RMSD value for ligands; (**c**) number of intermolecular hydrogen bonds; and (**d**) minimum distance between the proteins and each peptide ligand for the 50 ns duration.

The MD simulations results in Figure 7a–d determines the protein-ligand complexes stability during the 50 ns duration. In Figure 7a, the all-atom averaged root mean square deviation (RMSD) value for protein target Keap1, XO and MPO (chain A and B) in the complex were shown to be low at 2.14 ± 0.11 Å, 3.15 ± 0.33 Å, 2.59 ± 0.18 Å and 3.17 ± 0.36Å, respectively. In comparison, Figure 7b shows the all-atom averaged RMSD value of ligand LYSPH docked on each Keap1, XO and MPO were 1.73 ± 0.26 Å, 3.07 ± 0.33 Å and 3.34 ± 0.47 Å, respectively. This shows that RMSD values of both the docked proteins and the ligands are below the allowed limit [61], confirming that the protein-ligand complexes are stable over time. The plotted RMSD graphs also shows that receptor p47^phox^ took longer time to reach complex stability compared to the other docked proteins with the averaged RMSD value of 5.14 ± 0.13 Å, while all-atom averaged RMSD for its ligand, PSYLNTPLL was 4.36 ± 0.45 Å. The high p47^phox^ RMSD value was contributed by the flexibility of the N- and also C-terminal residues of the protein which reached up to 6.00 Å due to the loop structure of both terminals, visible by the root mean square fluctuations RMSF plot (Figure S1). The ligand interacted residues, however, were not affected and gave relatively low fluctuations during the 50 ns duration. In addition, the RMSD of PSYLNTPLL was also similarly low with LYSPH docked on other protein target (Figure 7b) during the 50 ns duration.

The dynamic intermolecular hydrogen bonds formed between the docked peptide and receptor protein were summarized in Figure 7c. The figure shows that highest number of intermolecular hydrogen bonds formed was in between LYSPH-XO (ave: 7), followed by PSYLNTPLL-p47^phox^ (ave: 5) and LYSPH-Keap1 (ave: 3). MPO protein was consists of chain A and chain B domain, where chain A formed only one intermolecular hydrogen bond with the ligand in average while chain B has the average of three intermolecular hydrogen bonds formed within the 50 ns duration. The polar group of the XO hot-spot region, and LYSPH peptide both contributed to the higher number of hydrogen bonds formed making the complex more stable [62]. Higher surface of interactions between PSYLNTPLL-p47^phox^ due to the longer sequence of the peptide had stabilized its docking on the active site of p47^phox^ [63]. The intermolecular hydrogen bonds formed in the complex had also correlated with the distance formed between each ligand and protein, as summarized in Figure 7d. The result shows that all complexes were tightly packed with the average protein-ligand distance of 1.43 Å–1.82 Å, except for LYSPH and the chain A of MPO which varied from 1.43 Å up to 5.31 Å. This was contributed by the binding site of the ligand which located closer to the chain B of MPO. Overall, the duration 50 ns were shown to be sufficient to evaluate the stability of protein-ligand complex formation where most of the ligand tends to reach conformational stability after 3 ns. The RMSF and radius of gyration (Rg) plots of each complex are available in Figures S1 and S2.

## 3. Materials and Methods

### 3.1. Compilation of a Virtual Library of Seed-Derived Antioxidant Peptides

**Figure 8:**
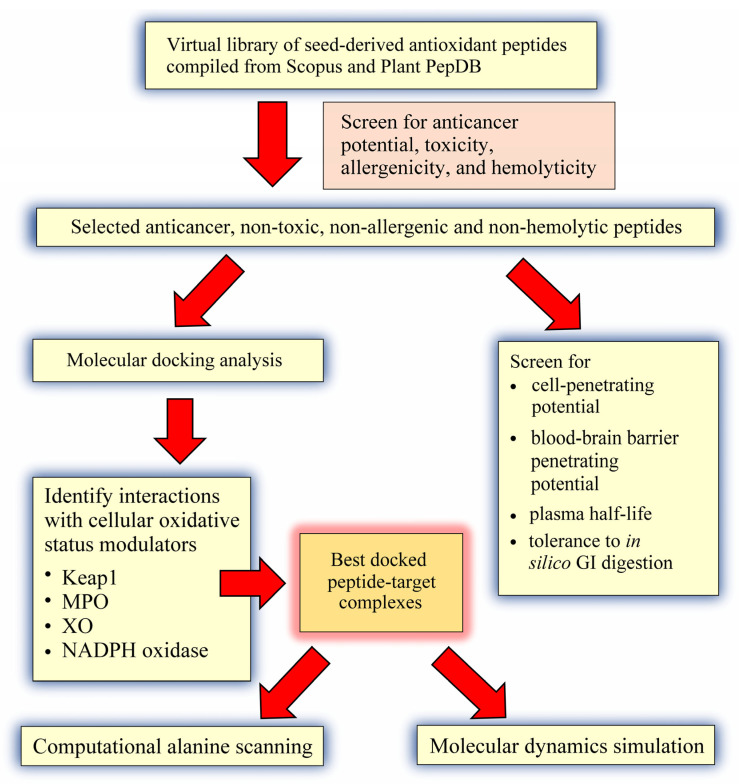
An overview of the computational approach used in this study.

Seed-derived antioxidant peptides were compiled from the publications in the Scopus database by using the search words listed in Table S2 (Accessed: 5–7 October 2021). A total of 63 publications were carefully examined to find antioxidant peptides identified from different seed sources. In addition, peptides were compiled from the PlantPepDB database (http://14.139.61.8/PlantPepDB/index.php) [21] by using “Simple Search”, searching “Antioxidant” and selecting “Peptide Activity” as search field (Accessed: 5–6 October 2021). Following the exclusion of redundant sequences, the resulting collection of seed-derived antioxidant peptide sequences was used in subsequent screening and molecular modelling analyses, as depicted in Figure 8.

### 3.2. Virtual Screening for Anticancer Potential, Toxicity, Allergenicity and Hemolyticity

Anticancer potential was predicted by using AntiCP 2.0 (https://webs.iiitd.edu.in/raghava/anticp2/index.html) [22] with the default SVM threshold of 0.45. Seed-derived antioxidant peptide sequences that were predicted as anticancer peptides by both Model 1 and Model 2 in the AntiCP 2.0 tool were noted. Toxicity was predicted by using ToxinPred (https://webs.iiitd.edu.in/raghava/toxinpred/index.html) [33] with the SVM threshold of 0.0, by using two methods: (i) SVM (Swiss-Prot) + Motif, and (ii) SVM (TrEMBL) + Motif. Only peptide sequences that were predicted as non-toxic by both of the aforementioned methods are regarded as non-toxic. Allergenicity was predicted by using AllerTOP v. 2.0 (https://www.ddg-pharmfac.net/AllerTOP/index.html) [32]. Hemolyticity was predicted by using HemoPI (https://webs.iiitd.edu.in/raghava/hemopi/index.php) [31] with the SVM + Motif (HemoPI-1) method. The aforementioned tools were accessed on 8–9 October 2021. Only seed-derived antioxidant peptides of 4–50 residues were screened as the peptides outside this range cannot be analyzed by AntiCP 2.0. The 2D structures of selected anticancer peptides were drawn by using the ACD/ChemSketch freeware (ACD/ChemSketch, version 2019.2.1, Advanced Chemistry Development, Inc., Toronto, ON, Canada, www.acdlabs.com, 2019). The molecular masses of peptides were calculated by using PepDraw (https://pepdraw.com/) (Accessed: 9 October 2021).

### 3.3. Molecular Docking Analysis

The 3D structures of peptides predicted to be anticancer, non-toxic, non-allergenic, and non-hemolytic were constructed by using PEP-FOLD 3 (https://bioserv.rpbs.univ-paris-diderot.fr/services/PEP-FOLD3/) [64,65,66] (Accessed: 9 October 2021). Two hundred simulations were run and the resulting models were sorted by the sOPEP method. The best output model of each peptide was downloaded and used in molecular docking.

The crystal structures of human Keap1 complexed with 16-mer peptide of Nrf2 (PDB ID: 2FLU) [39], human MPO complexed with 7GD (PDB ID: 6WYD) [41], bovine XO complexed with quercetin (PDB ID: 3NVY) [12], and p47^phox^ complexed with p22^phox^-derived proline-rich peptide (PDB ID: 1WLP) [14] were downloaded from the RCSB Protein Data Bank (https://www.rcsb.org/) [67] (Accessed: 9 October 2021). The separation of proteins and ligands from the crystals were performed by using BIOVIA Discovery Studio Visualizer (BIOVIA, Dassault Systèmes, BIOVIA Discovery Studio Visualizer, Version 20.1.0.192, San Diego: Dassault Systèmes, CA, USA, 2020).

For Keap1, MPO, and XO, the proteins were prepared as receptors in the PDBQT format after deleting water, adding polar hydrogen, and adding Kollman charges by using the AutoDock Tools 1.5.6 [25]. The prepared receptors were used for molecular docking on Webina 1.0.3 (https://durrantlab.pitt.edu/webina/) [24]. The co-crystalized ligands and the selected seed-derived peptides to be docked to the proteins were prepared as ligands and saved in the PDBQT format by using AutoDock Tools 1.5.6. Redocking of the co-crystalized ligands to Keap1, MPO and XO was performed using Webina 1.0.3 and RMSD was predicted by using LigRMSD v1.0 (https://ligrmsd.appsbio.utalca.cl/) [68]. For Keap1, redocking was performed by using tetrapeptide ETGE, the key motif of the 16-mer peptide of Nrf2, as recommended previously [40]. The coordinates of box center and box size used in molecular docking on Webina 1.0.3 were tabulated in Table S3. Molecular docking between p47^phox^ and the peptide ligands was performed by using the HPEPDOCK Server (http://huanglab.phys.hust.edu.cn/hpepdock/) [69,70,71,72,73]. Redocking of the co-crystalized p22^phox^-derived proline-rich peptide (GPLGSKQPPSNPPPRPPAEARKKPS) to p47^phox^ was also performed on HPEPDOCK and RMSD was predicted by using LigRMSD v1.0. Webina 1.0.3 and HPEPDOCK were accessed between 9 and 12 October 2021. Intermolecular interactions between proteins and peptides in selected docked models were analyzed and 2D interaction diagrams were generated by using LigPlot+ v.2.2.4 [74,75].

### 3.4. Prediction of Cell-Penetrating Potential, Blood-Brain Barrier Penetrating Potential, Plasma Half-Life, and Tolerance to In Silico GI Digestion

Cell-penetrating potential was predicted by using MLCPP (http://www.thegleelab.org/MLCPP/MLCPP.html) [23]. Blood-brain barrier penetrating potential was predicted by using B3Pred (https://webs.iiitd.edu.in/raghava/b3pred/index.html) [76] with Random- Forest (RF)-based prediction model and RF probability threshold of 0.1. Plasma half-life was predicted by using PlifePred (https://webs.iiitd.edu.in/raghava/plifepred/index.php) [77]. Tolerance to in silico GI digestion was predicted using the “enzyme(s) action” tool on BIOPEP-UWM (http://www.uwm.edu.pl/biochemia/index.php/en/biopep) [78] as previously reported [29]. The aforementioned tools were accessed on 18 October 2021.

### 3.5. Computational Alanine Scanning Mutagenesis

To assess the energetic contribution of individual residues in the selected seed-derived antioxidant peptides in conferring stability of binding to Keap1, MPO, XO, and p47^phox^, computational alanine scanning was performed by using BUDE Alanine Scan (https://pragmaticproteindesign.bio.ed.ac.uk/balas/) [79,80] (Accessed: 18–19 October 2021) as previously reported [27].

### 3.6. Molecular Dynamics Simulation

For a comprehensive analysis of the biomolecular dynamics, molecular dynamics (MD) simulation has evolved as the most powerful technique [26]. The detailed MD simulations of the complexes were conducted in GROMACS 2020 using the GROMOS96 54a7 force field [81]. The 54a7 force field was shown to improve the stability of α-helical structures in proteins and widely used in peptide simulations [82]. Molecular dynamics simulation was performed on each peptide ligand and protein complex of LYSPH-Keap1, LYSPH-MPO, LYSPH-XO, and PSYLNTPLL-p47^phox^ for 50 ns duration. In the MD, each complex was solvated in a cubic box with the distance of 1.2 nm between the complex and each side of the solvated box [83]. Sodium and chloride ions were added to neutralize the total charge of the system. The complex then was energy-minimized using the steepest descent algorithm [84]. The simulation condition was set at the room temperature (300 K) and the atmospheric pressure (1 bar) to closely mimic the general experiment conditions. The NVT thermal equilibration was carried out with a constrained structure and a velocity rescale thermostat specific to GROMACS, followed by NPT pressure equilibration was applied with the same velocity-rescale temperature coupling in addition to the Parrinello−Rahman pressure coupling [85]. The fully temperature and pressure equilibrated system was then used as the initial configuration for the MD production dynamic analysis. All simulations were conducted using a 2 fs time step [86]. The results were then analyzed using GROMACS functions such as RMSD and RMSF, while the formation of hydrogen bonds between each peptide and target proteins were analyzed using GROMACS “gmx_hbond” functions. Additionally, the distance between each protein and its ligand peptide was measured using the “gmx_pairdist” function.

## 4. Conclusions

Our computational study narrowed down the 677 peptides in the virtual library to five candidates predicted to have anticancer potential, in addition to non-toxicity, non-allergenicity and non-hemolyticity. Structure-based virtual screening found that LYSPH was the most promising peptide in targeting MPO, XO, and Keap1. On the other hand, PSYLNTPLL was the candidate that interacted most stably with p47^phox^. LYSPH and PSYLNTPLL were predicted to have cell- and blood-brain barrier penetrating potential. Taken together, LYSPH and PSYLNTPLL are two potential candidates of anticancer peptides that deserve more in-depth explorations, particularly wet-lab experimental validations, in future.

## Figures and Tables

**Table 1:** Intermolecular interactions between seed-derived antioxidant peptides and Keap1.

Peptide	Binding Affinity (kcal/mol)	Interaction with Keap1 ^a^		
		Hydrogen Bond	Hydrophobic Interaction	Salt Bridge
LYSPH	−7.6	**Arg380** **, Arg415(2), Arg483, Ser508**	**Tyr334**, **Ser363**, **Arg380**, **Asn382**, **Arg415**, **Ser508**, **Tyr525**, **Gln530**, Ala556, **Tyr572**, **Phe577**, **Ser602**	-
LPHFNS	−7.4	**Ser363**, **Arg380**, Asn414, **Arg415(2)**, **Gln530**	**Tyr334**, **Ser363**, **Arg380**, **Asn382**, Asn414, **Arg415**, **Arg483**, **Tyr525**, **Gln530**, **Ser555**, Ala556, **Tyr572**, **Phe577**, **Ser602**	-
AEHGSLH	−7.2	**Tyr334(2)**, **Arg380**, **Asn382(2)**, **Arg415**	**Tyr334**, Arg336, **Ser363**, **Arg380**, **Asn382**, Ser383, Pro384, **Arg415**, Ile461, **Arg483**, **Ser508**, **Tyr525**, **Gln530**, Ala556, **Tyr572**, **Ser602**	-
FGPEMEQ	−7.0	**Ser363**, **Arg380(2)**, **Asn382(2)**, **Asn387**, Asn414	**Tyr334**, **Ser363**, **Arg380**, **Asn382**, **Asn387**, Asp389, **Arg415**, Gly433, Ile461, **Ser555**, Ala556, **Tyr572**, **Phe577**	**Arg380(2)**
PSYLNTPLL	−6.4	**Arg380(2)**, **Asn382**, **Arg415**, **Arg483**, **Ser555**, **Tyr572**	**Tyr334**, **Ser363**, Gly364, **Arg380**, **Asn382**, **Arg415**, **Arg483**, **Tyr525**, **Gln530**, **Ser555**, Ala556, **Tyr572**, **Phe577**	-

^a^ Number in brackets indicates the number of hydrogen bonds or salt bridges formed with the same residue of Keap1. Keap1 residues that were reported to bind to ETGE (the key motif of Nrf2 peptide) [39] are marked in boldface type. Residues in the Keap1 binding pocket that were reported to contribute to stability of the Keap1:Nrf2 complex as evidenced by mutagenesis studies [39] are underlined.

**Table 2:** Intermolecular interactions between seed-derived antioxidant peptides and MPO.

Peptide	Binding Affinity (kcal/mol)	Interaction with MPO	
		Hydrogen Bond	Hydrophobic Interaction
LYSPH	−7.1	-	**Phe99**, Glu102, Phe146, Pro220, **Thr238**, **Arg239**, **Glu242**, **Phe366**, Phe407, Leu415, Leu420, Hec606
LPHFNS	−6.9	-	**Phe99**, Glu102, Glu116, Phe147, Pro220, **Thr238**, **Arg239**, **Phe366**, Phe407, Met411, Hec606
FGPEMEQ	−6.8	Glu102	**Phe99**, Thr100, Glu102, Glu116, Pro145, Phe147, Leu216, Pro220, **Thr238**, **Arg239**, **Glu242**, **Phe366**, Phe407, Met411, Leu415, Arg424, Hec606
AEHGSLH	−6.3	-	**Phe99**, Thr100, Glu102, Glu116, Pro145, Phe146, Phe147, **Thr238**, **Arg239**, **Glu242**, **Phe366**, Phe407, Val410, Met411, Leu415, Arg424, Hec606
PSYLNTPLL	−3.0	Thr100	**Phe99**, Glu102, Glu116, Pro145, Phe147, Leu216, Pro220, **Thr238**, **Arg239**, **Phe366**, Phe407, Val410, Met411, Arg412, Leu415, Hec606

MPO residues that were observed to interact with 7GD (co-crystalized inhibitor) based on LigPlot+ analysis of the crystal (PDB ID: 6WYD) are marked in boldface type. MPO residues that were reported to be involved in catalysis [44] are underlined.

**Table 3:** Intermolecular interactions between seed-derived antioxidant peptides and XO.

Peptide	Binding Affinity (kcal/mol)	Interaction with XO			
		Hydrogen Bond ^a^	Hydrophobic Interaction	Salt Bridge	External Bond
LYSPH	−6.2	Ser876	**Leu648**, Phe649, **Glu802**, **Leu873**, His875, Ser876, Glu879, **Phe914**, **Phe1009**, **Thr1010**, **Val1011**, Pro1012, **Phe1013**, **Leu1014**, Ala1078, Ala1079	-	-
FGPEMEQ	−5.9	**Glu802**, Ser876(2), Ala1079	**Leu648**, Phe649, Gln767, Phe798, Gly799, **Glu802**, Thr803, **Leu873**, His875, Ser876, Glu879, **Arg880**, Ala910, Phe911, Arg912, **Phe914**, **Phe1009**, **Thr1010**, **Val1011**, Pro1012, **Leu1014**, Pro1076, Ala1078, Ala1079, Ser1080, Glu1261	His875	-
LPHFNS	−4.7	His875, Ser876	**Leu648**, Phe649, **Glu802**, **Leu873**, His875, Ser876, Glu879, **Arg880**, **Phe914**, **Phe1009**, **Thr1010**, **Val1011**, Pro1012, **Phe1013**, **Leu1014**, Ala1078, Ala1079, Glu1261	-	-
AEHGSLH	−3.4	Glu879	**Leu648**, Phe649, Leu712, **Glu802**, **Leu873**, His875, Ser876, Glu879, **Phe914**, **Phe1009**, **Thr1010**, **Val1011**, Pro1012, **Phe1013**, **Leu1014**, Pro1076, Tyr1140, Phe1142	His875	-
PSYLNTPLL	3.0	Asn768, Asp872, Ser876(2), **Arg880**, **Thr1010(2)**	**Leu648**, Phe649, Leu712, Asn768, **Glu802**, Thr803, Arg871, Asp872, **Leu873**, Ser874, His875, Ser876, Glu879, **Arg880**, **Phe914**, Ser1008, **Phe1009**, **Thr1010**, **Val1011**, Pro1012, **Phe1013**, **Leu1014**, Pro1076, Ala1079, Tyr1140, Phe1142, Glu1261	-	Ala1079

^a^ Number in brackets indicates the number of hydrogen bonds formed with the same residue of XO. XO residues that were reported to bind to quercetin (co-crystalized inhibitor in the crystal PDB ID 3NVY) [12] are marked in boldface type. XO residues that were reported to be involved in catalysis [12] are underlined.

**Table 4:** Intermolecular interactions between seed-derived antioxidant peptides and p47^phox^.

Peptide	Docking Score	Interaction with p47^phox^	
		Hydrogen Bond	Hydrophobic Interaction
PSYLNTPLL	−216.493	**Trp193**, **Trp263**	Thr170, Ser171, Ser173, Glu174, Gly192, **Trp193**, **Trp204**, **Pro206**, Ser208, **Phe209**, Lys235, **Tyr237**, Asp261, Gly262, **Trp263**, **Met278**, **Tyr279**
LPHFNS	−195.377	**Tyr279**	**Tyr167**, Thr170, Ser191, **Trp193**, **Pro206**, **Tyr237**, **Asp243**, **Glu244**, Asp261, Gly262, **Trp263**, **Tyr274**, **Pro276**, **Tyr279**
LYSPH	−185.715	Thr170, Ser208	**Tyr167**, Thr170, Ser171, Ser173, Glu174, **Trp193**, **Trp204**, **Pro206**, Ser208, **Phe209**, **Trp263**, **Met278**
AEHGSLH	−181.729	**Trp263**	**Tyr167**, Thr170, Ser171, Glu174, **Trp193**, Glu241, **Asp243**, **Trp263**, **Tyr274**, **Pro276**, **Met278**
FGPEMEQ	−175.680	Thr170, **Trp193**	Thr170, Ser173, Glu174, Gly192, **Trp193**, **Trp204**, **Pro206**, **Phe209**, Asp261, **Met278**, **Tyr279**

p47^phox^ residues that were reported to bind to the ligand p22^phox^-derived proline-rich peptide in the crystal (PDB ID: 1WLP) [14] are marked in boldface type. Key residues that were reported for high-affinity binding between p47^phox^ and p22^phox^ as evidenced by mutagenesis studies [14] are underlined.

**Table 5:** The predictions of the cell-penetrating potential, blood-brain barrier penetrating potential, plasma half-life, and tolerance to in silico GI digestion of the five selected seed-derived antioxidant peptides.

Peptide	Cell-Penetrating Potential	Blood-Brain Barrier Penetrating Potential	Plasma Half-Life (Seconds)	Tolerance to In Silico GI Digestion
AEHGSLH	No	No	828.91	No
FGPEMEQ	No	Yes	796.21	No
LPHFNS	Yes	Yes	823.51	No
LYSPH	Yes	Yes	832.41	No
PSYLNTPLL	Yes	Yes	833.41	No

**Table 6:** Changes in the binding free energies (**∆∆**G) of the LYSPH- and PSYLNTPLL-protein complexes as revealed by the computational alanine scanning of the peptide residues.

Peptide	Residue	∆∆G (kJ/mol)			
		Keap1	MPO	XO	p47^phox^
LYSPH	Leu	8.4596	7.7764	6.3704	-
	Tyr	14.3232	23.0458	16.8951	-
	Ser	2.5540	−0.1909	−0.1396	-
	Pro	5.0370	5.2764	6.3965	-
	His	0.6399	3.1014	14.2670	-
PSYLNTPLL	Pro	-	-	-	0.0856
	Ser	-	-	-	0.5437
	Tyr	-	-	-	27.1615
	Leu	-	-	-	−0.4367
	Asn	-	-	-	0.7800
	Thr	-	-	-	1.1903
	Pro	-	-	-	2.9660
	Leu	-	-	-	2.7811
	Leu	-	-	-	0.4836

## Data Availability

The data presented in this study are available on request from the corresponding author.

## Supplementary Materials

Table S1: Seed-derived antioxidant peptides compiled from Scopus and PlantPepDB databases; Table S2: Search words used in Scopus to compile seed-derived antioxidant peptides; Table S3: Coordinates of box center and box size for different targets in molecular docking, and RMSD values; Table S4: Docking scores for peptides that were experimentally demonstrated to inhibit p47phox-p22^phox^ interaction and NADPH oxidase, in comparison with p22^phox^; Figure S1: RMSF plots for (**a**) PSYLNTPLL-p47phox, (**b**) LYSPH-Keap1, (**c**) LYSPH-XO, and (**d**) LYSPH-MPO; Figure S2: Gyration (Rg) plots of each complex.
