# Supplementary material for: Computational Screening for the Anticancer Potential of Seed-Derived Antioxidant Peptides: A Cheminformatic Approach
Source: Molecules. 2021 Dec 6;26(23):7396. doi: 10.3390/molecules26237396 (PMC8659047; doi:10.3390/molecules26237396)
Supplement: Supplementary file 1 [file molecules-26-07396-s001.zip › molecules-1493034-supplementary.pdf]

Table S1: Seed-derived antioxidant peptides compiled from Scopus and PlantPepDB databases

No.	Peptide	Source	Reported antioxidant activity	Reference
1	AW	Faba bean (*Vicia faba*, L.)	• ABTS radical scavenging activity<br>• DPPH radical scavenging activity<br>• Ferrous ion-chelating activity	(Samaei et al., 2021)
2	CA	Walnut (*Juglans Sigillata* Dode)	It is identified from D2 and D3. These fractions have high ORAC values in this study.	(Gu et al., 2015)
3	CR	Walnut (*Juglans Sigillata* Dode)	It is identified from D2 and D3. These fractions have high ORAC values in this study.	(Gu et al., 2015)
4	EL	Rice (*Oriza sativa L.* variety Japonica)	Antioxidant	(Daliri et al., 2020)
5	EM	Walnut (*Juglans Sigillata* Dode)	It is identified from D2 and D3. These fractions have high ORAC values in this study.	(Gu et al., 2015)
6	EW	Hazelnut (*Corylus heterophylla* Fisch)	• Increase viability of HUVEC cells that are treated with Angiotensin II<br>• Increase activity of CAT, T-SOD and GSH-Px<br>• Decrease content of LDH, MDA and ROS	(Fang et al., 2019)
7	FG	*Moringa oleifera* seed	• DPPH radical scavenging activity<br>• ABTS radical scavenging activity	(Liang et al., 2020)
8	FL	Wheat bran	It is identified from from <1 kDa protein hydrolysate, which has the highest ORAC values in this study.	(Zou et al., 2020)
9	FY	Perilla seed (*Perilla frutescens* L. Britton)	• ABTS radical scavenging activity<br>• Hydroxyl radical scavenging activity<br>• Superoxide anion radical scavenging activity<br>• Higher ORAC values than GSH<br>• Show linoleic acid peroxidation inhibition activity<br>• Inhibit lipid peroxidation in rat liver<br>• High concentration of FY induce apoptosis in HepG-2 cells (act as antioxidant and inhibit cancer proliferation)<br>• Increase cell ratios of HepG-2 cells that are exposed to H2O2	(Yang et al., 2018)
10	GY	*Moringa oleifera* seed	• DPPH radical scavenging activity<br>• ABTS radical scavenging activity	(Liang et al., 2020)
11	HC	Mung bean	• DPPH radical scavenging activity<br>• Hydroxyl radical scavenging activity<br>• Superoxide radical scavenging activity<br>• Ferric reducing antioxidant power<br>• Metal chelating activity	(Sonklin et al., 2021)
12	HH	Prince-of-Wales feather (*Amaranthus hypochondriacus*)	Antioxidant	(Silva-Sánchez et al., 2008)
		Soybean (*Glycine max*)		(Chen et al., 1996)
13	HL	Prince-of-Wales feather (*Amaranthus hypochondriacus*)	Antioxidant	(Silva-Sánchez et al., 2008)
		Soybean		(Chen et al., 1996)

		(*Glycine max*)		
14	IN	*Moringa oleifera* seed	• DPPH radical scavenging activity<br>• ABTS radical scavenging activity	(Liang et al., 2020)
15	IW	Faba bean (*Vicia faba*, L.)	• ABTS radical scavenging activity<br>• DPPH radical scavenging activity<br>• Ferrous ion-chelating activity	(Samaei et al., 2021)
16	IY	Soybean (*Glycine max*)	Antioxidant	(García et al., 2013)
		Prince-of-Wales feather (*Amaranthus hypochondriacus*)		(Silva-Sánchez et al., 2008)
		Moringa oleifera seed	• DPPH radical scavenging activity<br>• ABTS radical scavenging activity	(Liang et al., 2020)
17	LH	Prince-of-Wales feather (*Amaranthus hypochondriacus*)	Antioxidant	(Silva-Sánchez et al., 2008)
		Soybean (*Glycine max*)		(Chen et al., 1996)
18	LW	Faba bean (*Vicia faba*, L.)	• ABTS radical scavenging activity<br>• DPPH radical scavenging activity<br>• Ferrous ion-chelating activity	(Samaei et al., 2021)
19	LY	Soybean (*Glycine max*)	Antioxidant	(Beermann et al., 2009)
		Prince-of-Wales feather (*Amaranthus hypochondriacus*)		(Silva-Sánchez et al., 2008)
		Moringa oleifera seed	• DPPH radical scavenging activity<br>• ABTS radical scavenging activity	(Liang et al., 2020)
20	NL	Wheat bran	It is identified from from <1 kDa protein hydrolysate, which has the highest ORAC values in this study.	(Zou et al., 2020)
21	NW	Walnut (*Juglans Sigillata* Dode)	It is identified from D2 and D3. These fractions have high ORAC values in this study.	(Gu et al., 2015)
22	PW	Buckwheat (*Fagopyrum esculentum*)	Antioxidant	(Ma et al., 2010)
23	QL	Wheat bran	It is identified from from <1 kDa protein hydrolysate, which has the highest ORAC values in this study.	(Zou et al., 2020)
24	QY	*Moringa oleifera* seed	• DPPH radical scavenging activity<br>• ABTS radical scavenging activity<br>• Significantly increase cell viabilities of H_2O_2 treated Chang liver cell<br>• Significantly decrease contents of aspartate aminotransferase (ALT), alanine aminotransferase (AST), MDA<br>• Increase activities of SOD and CAT<br>• Reduce intracellular ROS level	(Liang et al., 2020)
25	SF	*Moringa oleifera* seed	• DPPH radical scavenging activity<br>• ABTS radical scavenging activity<br>• Significantly increase cell viabilities of H_2O_2 treated Chang liver cell<br>• Significantly decrease contents of aspartate aminotransferase (ALT),	(Liang et al., 2020)

			alanine aminotransferase (AST), MDA<br>• Increase activities of SOD and CAT<br>• Reduce intracellular ROS level	
26	SP	*Moringa oleifera* seed	• DPPH radical scavenging activity<br>• ABTS radical scavenging activity	(Liang et al., 2020)
27	VY	Faba bean (*Vicia faba*, L.)	• ABTS radical scavenging activity<br>• DPPH radical scavenging activity<br>• Ferrous ion-chelating activity	(Samaei et al., 2021)
28	WF	Purple wheat bran (*Triticum aestivum* L.)	• Superoxide anion radical scavenging activity<br>• Trolox equivalent antioxidant capacity	(Zhao, Zhao and Lu, 2020)
29	YA	Walnut (*Juglans Sigillata* Dode)	It is identified from D2 and D3. These fractions have high ORAC values in this study.	(Gu et al., 2015)
30	YG	Walnut (*Juglans Sigillata* Dode)	It is identified from D2 and D3. These fractions have high ORAC values in this study.	(Gu et al., 2015)
31	YK	Walnut (*Juglans Sigillata* Dode)	It is identified from D2 and D3. These fractions have high ORAC values in this study.	(Gu et al., 2015)
32	YL	Korean perilla (*Perilla frutescens*)	• ABTS radical scavenging activity<br>• Hydroxyl radical scavenging activity<br>• Superoxide anion radical scavenging activity<br>• Higher ORAC values than GSH<br>• Show linoleic acid peroxidation inhibition activity<br>• Inhibit lipid peroxidation in rat liver<br>• Increase cell ratios of HepG-2 cells that are exposed to H_2O_2	(Yang et al., 2018)
		Prince-of-Wales feather (*Amaranthus hypochondriacus*)	Antioxidant	(Silva-Sánchez et al., 2008)
33	YS	Walnut (*Juglans Sigillata* Dode)	It is identified from D2 and D3. These fractions have high ORAC values in this study.	(Gu et al., 2015)
34	YT	Walnut (*Juglans Sigillata* Dode)	It is identified from D2 and D3. These fractions have high ORAC values in this study.	(Gu et al., 2015)
35	YV	Faba bean (*Vicia faba*, L.)	• ABTS radical scavenging activity<br>• DPPH radical scavenging activity<br>• Ferrous ion-chelating activity	(Samaei et al., 2021)
36	ADF	Soybean (*Glycine max*)	Antioxidant	(Yokomizo, Takenaka and Takenaka, 2002)
37	AHH	Chickpea seeds	• Reducing power activity<br>• DPPH radical scavenging activity<br>• Inhibit peroxyl radical-induced DCFH oxidation to DCF in Caco-2 cells	(Torres-Fuentes et al., 2015)
38	AYL	Corn gluten	• DPPH radical scavenging activity<br>• Hydroxyl radical scavenging activity<br>• Reducing ability	(Ren et al., 2018)
39	CGN	Mung bean	• DPPH radical scavenging activity<br>• Hydroxyl radical scavenging activity	(Sonklin et al., 2021)

			• Superoxide radical scavenging activity<br>• Ferric reducing antioxidant power<br>• Metal chelating activity	
40	CHC	Walnut (*Juglans Sigillata* Dode)	It is identified from D2 and D3. These fractions have high ORAC values in this study.	(Gu et al., 2015)
41	CTN	Mung bean	• DPPH radical scavenging activity<br>• Hydroxyl radical scavenging activity<br>• Superoxide radical scavenging activity<br>• Ferric reducing antioxidant power<br>• Metal chelating activity	(Sonklin et al., 2021)
42	ERF	Mung bean	• DPPH radical scavenging activity except<br>• Hydroxyl radical scavenging activity except<br>• Superoxide radical scavenging activity<br>• Ferric reducing antioxidant power<br>• Metal chelating activity	(Sonklin et al., 2021)
43	EYW	Mung bean	• DPPH radical scavenging activity<br>• Superoxide radical scavenging activity<br>• Ferric reducing antioxidant power<br>• Metal chelating activity	(Sonklin et al., 2021)
44	GHC	Walnut (*Juglans Sigillata* Dode)	It is identified from D2 and D3. These fractions have high ORAC values in this study.	(Gu et al., 2015)
45	GSQ	Chinese leek seed (*Allium tuberosum* Rottler)	• DPPH radical scavenging activity<br>• ABTS radical scavenging activity<br>• Superoxide radical scavenging activity<br>• Reducing power<br>• Inhibition of linoleic acid peroxidation<br>• Increase cell survival rate of LO_2 cells which was treated with H_2O_2	(Hong et al., 2014)
46	GYY	Prince-of-Wales feather (*Amaranthus hypochondriacus*)	Antioxidant	(Silva-Sánchez et al., 2008)
		Soybean (*Glycine max*)		(Yokomizo, Takenaka and Takenaka, 2002)
47	HAL	Wheat bran	It is identified from from <1 kDa protein hydrolysate, which has the highest ORAC values in this study.	(Zou et al., 2020)
48	HHP	Soybean (*Glycine max*)	Antioxidant	(Chen et al., 1996)
49	HLH	Soybean (*Glycine max*)	Antioxidant	(Chen et al., 1996)
50	HPH	Soybean (*Glycine max*)	Antioxidant	(Chen et al., 1996)
51	LAF	Mung bean	• Hydroxyl radical scavenging activity<br>• Superoxide radical scavenging activity<br>• Ferric reducing antioxidant power<br>• Metal chelating activity	(Sonklin et al., 2021)
52	LAN	Mung bean	• Hydroxyl radical scavenging activity except	(Sonklin et al., 2021)

			• Superoxide radical scavenging activity<br>• Ferric reducing antioxidant power<br>• Metal chelating activity	
53	LHH	Prince-of-Wales feather (*Amaranthus hypochondriacus*)	Antioxidant	(Silva-Sánchez et al., 2008)
		Soybean (*Glycine max*)		(Chen et al., 1996)
54	LPC	Walnut (*Juglans Sigillata* Dode)	It is identified from D2 and D3. These fractions have high ORAC values in this study.	(Gu et al., 2015)
55	LPH	Chickpea seeds	• Reducing power activity<br>• DPPH radical scavenging activity<br>• Inhibit peroxyl radical-induced DCFH oxidation to DCF in Caco-2 cells	(Torres-Fuentes et al., 2015)
56	LPL	Hemp seed (*Cannabis sativa* L.)	• DPPH radical scavenging activity<br>• Metal chelation activity	(Girgih et al., 2014)
57	LQL	Hemp seed (*Cannabis sativa* L.)	• DPPH radical scavenging activity<br>• Metal chelation activity	(Girgih et al., 2014)
58	MEN	Corn germ	It is identified from F3 fraction of CGMH-5, which has high<br>• DPPH radical scavenging activity<br>• ABTS radical scavenging activity<br>• ORAC values<br>• effectiveness against peroxyl radicals<br>• reduction activity of ROS generation in cell which was treated with H_2O_2<br>• hydroxyl radical scavenging activity	(S, Zhang et al., 2019)
59	MNN	Corn germ	It is identified from F3 fraction of CGMH-5, which has high<br>• DPPH radical scavenging activity<br>• ABTS radical scavenging activity<br>• ORAC values<br>• effectiveness against peroxyl radicals<br>• reduction activity of ROS generation in cell which was treated with H_2O_2<br>• hydroxyl radical scavenging activity	(S, Zhang et al., 2019)
60	PFE	*Moringa oleifera* seed	• DPPH radical scavenging activity<br>• ABTS radical scavenging activity	(Liang et al., 2020)
61	PHH	Soybean (*Glycine max*)	Antioxidant	(Chen et al., 1996)
62	PYY	Soybean (*Glycine max*)	Antioxidant	(Chen et al., 1996)
63	QAC	Purple wheat bran (*Triticum aestivum* L.)	• Superoxide anion radical scavenging activity<br>• Trolox equivalent antioxidant capacity	(Zhao, Zhao and Lu, 2020)
64	RNF	Purple wheat bran (*Triticum aestivum* L.)	• Superoxide anion radical scavenging activity	(Zhao, Zhao and Lu, 2020)

			• Trolox equivalent antioxidant capacity	
65	RPR	Chickpea seeds	• Reducing power activity<br>• DPPH radical scavenging activity<br>• Inhibit peroxyl radical-induced DCFH oxidation to DCF in Caco-2 cells	(Torres-Fuentes et al., 2015)
66	SDF	Soybean (*Glycine max*)	Antioxidant	(Yokomizo, Takenaka and Takenaka, 2002)
67	SQK	Walnut (*Juglans Sigillata* Dode)	It is identified from D2 and D3. These fractions have high ORAC values in this study.	(Gu et al., 2015)
68	SSC	Purple wheat bran (*Triticum aestivum* L.)	• Superoxide anion radical scavenging activity<br>• Trolox equivalent antioxidant capacity	(Zhao, Zhao and Lu, 2020)
69	VPW	Buckwheat (*Fagopyrum esculentum*)	Antioxidant	(Ma et al., 2010)
70	WAF	Palm Kernel cake	• DPPH radical scavenging activity<br>• Metal chelating activity	(Zarei et al., 2014)
71	WPL	Buckwheat (*Fagopyrum esculentum*)	Antioxidant	(Ma et al., 2010)
72	WSY	Hemp seed (*Cannabis sativa* L.)	• DPPH radical scavenging activity<br>• Metal chelation activity	(Girgih et al., 2014)
73	WYT	Hemp seed (*Cannabis sativa* L.)	• DPPH radical scavenging activity<br>• Metal chelation activity	(Girgih et al., 2014)
74	YFE	*Moringa oleifera* seed	• DPPH radical scavenging activity<br>• ABTS radical scavenging activity	(Liang et al., 2020)
75	YGS	Peanut meal (*Arachis hypogaea* L.)	• Higher ORAC value than GSH<br>• Inhibit oxidation of linoleic acid<br>• Enhance cell viability of PC12 cells that are treated with H_2O_2	(Zheng et al., 2012)
76	YHH	Soybean (*Glycine max*)	Antioxidant	(Chen et al., 1996)
77	YNI	Hemp seed (*Cannabis sativa* L.)	• DPPH radical scavenging activity<br>• Metal chelation activity	(Girgih et al., 2014)
78	YNL	Hemp seed (*Cannabis sativa* L.)	• DPPH radical scavenging activity<br>• Metal chelation activity	(Girgih et al., 2014)
79	YPQ	Corn gluten	• DPPH radical scavenging activity<br>• Hydroxyl radical scavenging activity<br>• Reducing ability	(Ren et al., 2018)
80	YSL	Rice (*Oryza sativa*)	Antioxidant	(Wang et al., 2017)
81	YTR	*Moringa oleifera* seed	• DPPH radical scavenging activity<br>• ABTS radical scavenging activity	(Liang et al., 2020)
82	AAVL	Wheat bran	It is identified from from <1 kDa protein hydrolysate, which has the highest ORAC values in this study.	(Zou et al., 2020)
83	ADGF	Hazelnut (*Corylus heterophylla* Fisch)	• Increase viability of HUVEC cells that are treated with Angiotensin II<br>• Increase activity of CAT, T-SOD and GSH-Px<br>• Decrease content of LDH, MDA and ROS	(Fang et al., 2019)
			• ABTS scavenging activity<br>• DPPH scavenging activity	(Liu et al., 2018)

84	AGGF	Hazelnut (*Corylus heterophylla* Fisch)	• Increase viability of HUVEC cells that are treated with Angiotensin II	(Fang et al., 2019)
			• ABTS radical scavenging activity<br>• DPPH radical scavenging activity	(Liu et al., 2018)
85	AWFS	Palm Kernel cake	• DPPH radical scavenging activity<br>• Metal chelating activity	(Zarei et al., 2014)
86	CSGD	Mung bean	• DPPH radical scavenging activity<br>• Hydroxyl radical scavenging activity<br>• Superoxide radical scavenging activity<br>• Ferric reducing antioxidant power<br>• Metal chelating activity	(Sonklin et al., 2021)
87	DHHQ	Rice (*Oryza sativa*)	Antioxidant	(Wei et al., 2007)
88	EAAY	Feng Dan seed (*Paeonia ostia*)	• ABTS radical scavenging activity<br>• Hydroxyl radical scavenging activity	(Wang et al., 2019)
89	EFIQ	Hemp seed (*Cannabis sativa* L.)	• DPPH radical scavenging activity<br>• Metal chelation activity	(Girgih et al., 2014)
90	EFLQ	Hemp seed (*Cannabis sativa* L.)	• DPPH radical scavenging activity<br>• Metal chelation activity	(Girgih et al., 2014)
91	EFQI	Hemp seed (*Cannabis sativa* L.)	• DPPH radical scavenging activity<br>• Metal chelation activity	(Girgih et al., 2014)
92	EFQL	Hemp seed (*Cannabis sativa* L.)	• DPPH radical scavenging activity<br>• Metal chelation activity	(Girgih et al., 2014)
93	ETTL	Hazelnut (*Corylus heterophylla* Fisch)	Increase viability of HUVEC cells that are treated with Angiotensin II	(Fang et al., 2019)
			• ABTS scavenging activity<br>• DPPH scavenging activity	(Liu et al., 2018)
94	FEQI	Hemp seed (*Cannabis sativa* L.)	• DPPH radical scavenging activity<br>• Metal chelation activity	(Girgih et al., 2014)
95	FEQL	Hemp seed (*Cannabis sativa* L.)	• DPPH radical scavenging activity<br>• Metal chelation activity	(Girgih et al., 2014)
96	FEYL	Peach seed (*Prunus persica* (L.) *Batsch*)	It is identified from the whole Thermolysin extract, and this extract has<br>• ABTS radical scavenging activity<br>• hydroxyl radical scavenging activity<br>• lipid peroxidation inhibition activity<br>• ferric reducing antioxidant power	(Vásquez-Villanueva, Marina and García, 2016)
97	FLPF	Emmer (*Triticum dicoccum*)	Antioxidant	(Babini et al., 2017)
98	FLQL	Mung bean	Ferric reducing antioxidant power	(Sonklin et al., 2021)
99	FSAP	Feng Dan seed (*Paeonia ostia*)	• ABTS radical scavenging activity<br>• Hydroxyl radical scavenging activity	(Wang et al., 2019)
100	FVPH	Chickpea (*Cicer arietinum*)	Antioxidant	(Torres-Fuentes et al., 2015)
101	GGIF	Palm Kernel cake	• DPPH radical scavenging activity<br>• Metal chelating activity	(Zarei et al., 2014)
102	GIFE	Palm Kernel cake	• DPPH radical scavenging activity<br>• Metal chelating activity	(Zarei et al., 2014)

103	HGDE	Chickpea seeds	• Reducing power activity<br>• DPPH radical scavenging activity<br>• Inhibit peroxyl radical-induced DCFH oxidation to DCF in Caco-2 cells	(Torres-Fuentes et al., 2015)
104	HHLP	Soybean (*Glycine max*)	Antioxidant	(Chen et al., 1996)
105	HHPL	Soybean (*Glycine max*)	Antioxidant	(Chen et al., 1996)
106	HLHP	Soybean (*Glycine max*)	Antioxidant	(Chen et al., 1996)
107	HLLP	Peach seed (*Prunus persica* (L.) *Batsch*)	It is identified from the whole Thermolysin extract, and this extract has<br>• ABTS radical scavenging activity<br>• hydroxyl radical scavenging activity<br>• lipid peroxidation inhibition activity<br>• ferric reducing antioxidant power	(Vásquez-Villanueva, Marina and García, 2016)
108	HLPH	Soybean (*Glycine max*)	Antioxidant	(Chen et al., 1996)
109	HPHL	Soybean (*Glycine max*)	Antioxidant	(Chen et al., 1996)
110	HPLH	Soybean (*Glycine max*)	Antioxidant	(Chen et al., 1996)
111	KPKL	Kidney bean (*Phaseolus vulgaris*)	Antioxidant	(Mojica and de Mejía, 2015)
112	LHPH	Soybean (*Glycine max*)	Antioxidant	(Chen et al., 1996)
113	LLAH	Kidney bean (*Phaseolus vulgaris*)	Antioxidant	(Mojica and de Mejía, 2015)
114	LLES	Cherry (*Prunus cerasus*)	Antioxidant	(García et al., 2015)
115	LLHH	Soybean (*Glycine max*)	Antioxidant	(Chen et al., 1996)
116	LLMQ	Cherry (*Prunus cerasus*)	Antioxidant	(García et al., 2015)
117	LLPH	Soybean (*Glycine max*)	Antioxidant	(Chen et al., 1996)
		Chickpea seeds	• Reducing power activity<br>• DPPH radical scavenging activity<br>• Inhibit peroxyl radical-induced DCFH oxidation to DCF in Caco-2 cells	(Torres-Fuentes et al., 2015)
118	LLTC	Wheat (*Triticum aestivum*)	Antioxidant	(Babini et al., 2017)
119	LPHH	Soybean (*Glycine max*)	Antioxidant	(Chen et al., 1996)
120	LPYY	Soybean (*Glycine max*)	Antioxidant	(Chen et al., 1996)
121	LQAE	Cherry (*Prunus cerasus*)	Antioxidant	(García et al., 2015)
122	LTAH	Peach seed (*Prunus persica* (L.) *Batsch*)	It is identified from the whole Thermolysin extract, and this extract has<br>• ABTS radical scavenging activity<br>• hydroxyl radical scavenging activity	(Vásquez-Villanueva, Marina and García, 2016)

			• lipid peroxidation inhibition activity<br>• ferric reducing antioxidant power	
123	MFIF	Wheat (*Triticum aestivum*)	Antioxidant	(Babini et al., 2017)
124	MFLF	Emmer (*Triticum dicoccum*)	DPPH radical scavenging activity	(Babini et al., 2017)
125	MGGN	Corn germ	It is identified from F3 fraction of CGMH-5, which has high<br>• DPPH radical scavenging activity<br>• ABTS radical scavenging activity<br>• ORAC values<br>• effectiveness against peroxyl radicals<br>• reduction activity of ROS generation in cell which was treated with H_2O_2<br>• hydroxyl radical scavenging activity	(S, Zhang et al., 2019)
126	MMGW	Mung bean	• DPPH radical scavenging activity<br>• Hydroxyl radical scavenging activity<br>• Superoxide radical scavenging activity<br>• Ferric reducing antioxidant power<br>• Metal chelating activity	(Sonklin et al., 2021)
127	PLHH	Soybean (*Glycine max*)	Antioxidant	(Chen et al., 1996)
128	QFAW	Mung bean	• DPPH radical scavenging activity<br>• Ferric reducing antioxidant power<br>• Metal chelating activity	(Sonklin et al., 2021)
129	RQPH	Chickpea seeds	• Reducing power activity<br>• DPPH radical scavenging activity<br>• Inhibit peroxyl radical-induced DCFH oxidation to DCF in Caco-2 cells	(Torres-Fuentes et al., 2015)
130	SGAF	Hazelnut (*Corylus heterophylla* Fisch)	Increase viability of HUVEC cells that are treated with Angiotensin II	(Fang et al., 2019)
			• ABTS scavenging activity<br>• DPPH scavenging activity	(Liu et al., 2018)
131	SVYT	Hemp seed (*Cannabis sativa* L.)	• DPPH radical scavenging activity<br>• Metal chelation activity	(Girgih et al., 2014)
132	TKGQ	Chinese chestnut (*Castanea mollissima* Blume)	ABTS radical scavenging activity	(Feng et al., 2018)
133	TTYY	Soybean (*Glycine max*)	Antioxidant	(Beermann et al., 2009)
134	VFPW	Buckwheat (*Fagopyrum esculentum*)	Antioxidant	(Ma et al., 2010)
135	VKMC	Emmer (*Triticum dicoccum*)	DPPH radical scavenging activity	(Babini et al., 2017)
136	VLTQ	Peach seed (*Prunus persica* (L.) *Batsch*)	It is identified from the whole Thermolysin extract, and this extract has<br>• ABTS radical scavenging activity	(Vásquez-Villanueva, Marina and García, 2016)

			• hydroxyl radical scavenging activity<br>• lipid peroxidation inhibition activity<br>• ferric reducing antioxidant power	
137	VLYL	Peach seed (*Prunus persica* (L.) *Batsch*)	It is identified from the whole Thermolysin extract, and this extract has<br>• ABTS radical scavenging activity<br>• hydroxyl radical scavenging activity<br>• lipid peroxidation inhibition activity<br>• ferric reducing antioxidant power	(Vásquez-Villanueva, Marina and García, 2016)
138	VLYN	Peach seed (*Prunus persica* (L.) *Batsch*)	It is identified from the whole Thermolysin extract, and this extract has<br>• ABTS radical scavenging activity<br>• hydroxyl radical scavenging activity<br>• lipid peroxidation inhibition activity<br>• ferric reducing antioxidant power	(Vásquez-Villanueva, Marina and García, 2016)
139	VSYT	Hemp seed (*Cannabis sativa* L.)	• DPPH radical scavenging activity<br>• Metal chelation activity	(Girgih et al., 2014)
140	VVNE	Cherry (*Prunus cerasus*)	Antioxidant	(García et al., 2015)
141	VYTE	Chinese chestnut (*Castanea mollissima* Blume)	ABTS radical scavenging activity	(Feng et al., 2018)
142	WAFS	Palm Kernel cake	• DPPH radical scavenging activity<br>• Metal chelating activity	(Zarei et al., 2014)
143	WVYY	Hemp seed (*Cannabis sativa*)	Antioxidant	(Girgih et al., 2014; Girgih, He and Aluko, 2014)
144	YSVH	Walnut (*Juglans Sigillata* Dode)	It is identified from D2 and D3. These fractions have high ORAC values in this study.	(Gu et al., 2015)
145	YVGD	*Ginkgo biloba* seeds	• DPPH radical scavenging activity<br>• Hydroxyl radical scavenging activity<br>• Superoxide radical scavenging activity	(Wu et al., 2013)
146	Ac-QWFCT	Pine nut meal (*P. koraiensis*)	• FRAP<br>• DPPH radical scavenging activity<br>• ABTS radical scavenging activity<br>• Reduce dichlorofluorescein (DCF) formation	(Yang et al., 2017)
147	AFGPE	Cherry (*Prunus cerasus*)	Antioxidant	(García et al., 2015)
148	AIVIL	Mung bean	• DPPH radical scavenging activity<br>• Hydroxyl radical scavenging activity<br>• Superoxide radical scavenging activity<br>• Metal chelating activity<br>• Ferric reducing antioxidant power	(Chunkao et al., 2020)

149	AKTVF	Wheat bran	It is identified from from <1 kDa protein hydrolysate, which has the highest ORAC values in this study.	(Zou et al., 2020)
150	AWDPE	Hazelnut (*Corylus heterophylla* Fisch)	• ABTS scavenging activity<br>• DPPH scavenging activity	(Liu et al., 2018)
151	CAAIC	Emmer (*Triticum dicoccum*)	DPPH radical scavenging activity	(Babini et al., 2017)
152	DDLPR	Olive seeds (*Olea europaea*)	Antioxidant	(Esteve, Marina and García, 2015)
153	DEVPR	Cherry (*Prunus cerasus*)	Antioxidant	(García et al., 2015)
154	DWDPK	Hazelnut (*Corylus heterophylla* Fisch)	• ABTS radical scavenging activity<br>• DPPH radical scavenging activity	(Liu et al., 2018)
155	FGSLH	Chickpea seeds	• Reducing power activity<br>• DPPH radical scavenging activity<br>• Inhibit peroxyl radical-induced DCFH oxidation to DCF in Caco-2 cells	(Torres-Fuentes et al., 2015)
156	FVLGL	Cherry (*Prunus cerasus*)	Antioxidant	(García et al., 2015)
157	FVLPH	Cottonseed	• DPPH radical scavenging activity<br>• ABTS radical scavenging activity<br>• hydroxyl radical scavenging activity<br>• Fe^{2+} chelating activities	(Wang et al., 2021)
158	HADAD	Mung bean	• DPPH radical scavenging activity<br>• Hydroxyl radical scavenging activity<br>• Superoxide radical scavenging activity<br>• Metal chelating activity<br>• Ferric reducing antioxidant power	(Chunkao et al., 2020)
159	HHPLL	Soybean (*Glycine max*)	Antioxidant	(Chen et al., 1996)
160	HKNAM	Chickpea seeds	• Reducing power activity<br>• DPPH radical scavenging activity<br>• Inhibit peroxyl radical-induced DCFH oxidation to DCF in Caco-2 cells	(Torres-Fuentes et al., 2015)
161	IEEAF	Hemp seed (*Cannabis sativa* L.)	• DPPH radical scavenging activity<br>• Metal chelation activity	(Girgih et al., 2014)
162	IFVPH	Cottonseed	• DPPH radical scavenging activity<br>• ABTS radical scavenging activity<br>• Hydroxyl radical scavenging activity<br>• Fe^{2+} chelating activities	(Wang et al., 2021)
163	IPAGM	Hemp seed (*Cannabis sativa* L.)	• DPPH radical scavenging activity<br>• Metal chelation activity	(Girgih et al., 2014)
164	IPAGV	Hemp seed (*Cannabis sativa* L.)	• DPPH radical scavenging activity<br>• Metal chelation activity	(Girgih et al., 2014)
165	IPSQV	Soybean (*Glycine max*)	• ABTS radical scavenging activity<br>• Peroxyl radical scavenging capacity<br>• Increased No_2^- production<br>• Decrease in TBARS levels (lipid peroxidation)	(Amigo-Benavent et al., 2014)

			• Increase in H-Caco-2 cells of MnSOD and CAT activity	
166	KDHCH	Pine nut	• DPPH radical scavenging activity<br>• ABTS radical scavenging activity<br>• Improve ROS scavenging or inhibiting ability<br>• Increase CAT and SOD activity	(Ma et al., 2019)
167	KFFPS	Cottonseed	• DPPH radical scavenging activity<br>• ABTS radical scavenging activity<br>• hydroxyl radical scavenging activity<br>• Fe^{2+} chelating activities	(Wang et al., 2021)
168	KGAIG	Soybean (*Glycine max*)	Antioxidant	(Puchalska, Concepción García and Luisa Marina, 2014)
169	KGALG	Soybean (*Glycine max*)	Antioxidant	(Puchalska, Concepción García and Luisa Marina, 2014)
170	KGIFG	Soybean (*Glycine max*)	Antioxidant	(Puchalska, Concepción García and Luisa Marina, 2014)
171	KGLFG	Soybean (*Glycine max*)	Antioxidant	(Puchalska, Concepción García and Luisa Marina, 2014)
172	KHFLA	Soybean (*Glycine max*)	Antioxidant	(Puchalska, Concepción García and Luisa Marina, 2014)
173	KKVVC	Emmer (*Triticum dicoccum*)	DPPH radical scavenging activity	(Babini et al., 2017)
174	KNILE	Soybean (*Glycine max*)	Antioxidant	(Puchalska, Concepción García and Luisa Marina, 2014)
175	KSALH	Emmer (*Triticum dicoccum*)	DPPH radical scavenging activity	(Babini et al., 2017)
176	KWFCT	Pine nut meal (*P. koraiensis*)	• FRAP<br>• DPPH radical scavenging activity<br>• ABTS radical scavenging activity<br>• Reduce dichlorofluorescein (DCF) formation	(Yang et al., 2017)
177	LALPA	Hemp seed (*Cannabis sativa* L.)	• DPPH radical scavenging activity<br>• Metal chelation activity	(Girgih et al., 2014)
178	LEEAF	Hemp seed (*Cannabis sativa* L.)	• DPPH radical scavenging activity<br>• Metal chelation activity	(Girgih et al., 2014)
179	LFSPR	Cherry (*Prunus cerasus*)	Antioxidant	(García et al., 2015)
180	LHLPS	Peach seed (*Prunus persica* (L.) *Batsch*)	It is identified from the whole Thermolysin extract, and this extract has<br>• ABTS radical scavenging activity<br>• hydroxyl radical scavenging activity<br>• lipid peroxidation inhibition activity	(Vásquez-Villanueva, Marina and García, 2016)

			• ferric reducing antioxidant power	
181	LLAQA	Cherry (*Prunus cerasus*)	Antioxidant	(García et al., 2015)
182	LLDQE	Peach seed (*Prunus persica* (L.) *Batsch*)	It is identified from the whole Thermolysin extract, and this extract has<br>• ABTS radical scavenging activity<br>• hydroxyl radical scavenging activity<br>• lipid peroxidation inhibition activity<br>• ferric reducing antioxidant power	(Vásquez-Villanueva, Marina and García, 2016)
183	LLGIL	Mung bean	• DPPH radical scavenging activity<br>• Hydroxyl radical scavenging activity<br>• Superoxide radical scavenging activity<br>• Metal chelating activity	(Chunkao et al., 2020)
184	LLLGI	Mung bean	• Hydroxyl radical scavenging activity<br>• Superoxide radical scavenging activity<br>• Metal chelating activity<br>• Ferric reducing antioxidant power	(Chunkao et al., 2020)
185	LLLLG	Mung bean	• DPPH radical scavenging activity<br>• Hydroxyl radical scavenging activity<br>• Superoxide radical scavenging activity<br>• Metal chelating activity	(Chunkao et al., 2020)
186	LLNDE	Cherry (*Prunus cerasus*)	Antioxidant	(García et al., 2015)
187	LLPHH	Soybean (*Glycine max*)	Antioxidant	(Chen, Muramoto and Yamauchi, 1995)
188	LPAGV	Hemp seed (*Cannabis sativa* L.)	• DPPH radical scavenging activity<br>• Metal chelation activity	(Girgih et al., 2014)
189	LPHFN	Chickpea seeds	• Reducing power activity<br>• DPPH radical scavenging activity<br>• Inhibit peroxyl radical-induced DCFH oxidation to DCF in Caco-2 cells	(Torres-Fuentes et al., 2015)
190	LYSPH	Cherry (*Prunus cerasus*)	Antioxidant	(García et al., 2015)
191	LYTPH	Cherry (*Prunus cerasus*)	Antioxidant	(García et al., 2015)
192	MDGAP	Olive seeds (*Olea europaea*)	Antioxidant	(Esteve, Marina and García, 2015)
193	MGSPT	Olive seeds (*Olea europaea*)	Antioxidant	(Esteve, Marina and García, 2015)
194	MMLQK	Chinese chestnut (*Castanea mollissima* Blume)	ABTS radical scavenging activity	(Feng et al., 2018)
195	NFHPQ	Rice (*Oryza sativa*)	Antioxidant	(Yan et al., 2015)
196	NGRFY	Cottonseed	• DPPH radical scavenging activity<br>• ABTS radical scavenging activity	(Wang et al., 2021)

			• hydroxyl radical scavenging activity<br>• Fe^{2+} chelating activities	
197	NLPLL	Cherry (*Prunus cerasus*)	Antioxidant	(García et al., 2015)
198	NQLDQ	Soybean (*Glycine max*)	Antioxidant	(Puchalska, Concepción García and Luisa Marina, 2014)
199	NRYHE	Chickpea (*Cicer arietinum* L.)	• DPPH radical scavenging activity<br>• Hydroxyl radical scavenging activity<br>• Superoxide radical scavenging activity<br>• Cu^{2+} and Fe^{2+} chelating activity<br>• Inhibit oxidation of linoleic acid	(Zhang et al., 2011)
200	PAIDL	Mung bean	• DPPH radical scavenging activity<br>• Hydroxyl radical scavenging activity<br>• Superoxide radical scavenging activity<br>• Metal chelating activity<br>• Ferric reducing antioxidant power	(Chunkao et al., 2020)
201	PMPVR	Semen cassiae (*Cassia obtusifolia* L.)	ABTS radical scavenging activity	(Chai et al., 2019)
202	PSIPA	Hemp seed (*Cannabis sativa* L.)	• DPPH radical scavenging activity<br>• Metal chelation activity	(Girgih et al., 2014)
203	PSLPA	Hemp seed (*Cannabis sativa*)	Antioxidant	(Girgih et al., 2014)
204	QERHQ	Chickpea seeds	• Reducing power activity<br>• DPPH radical scavenging activity<br>• Inhibit peroxyl radical-induced DCFH oxidation to DCF in Caco-2 cells	(Torres-Fuentes et al., 2015)
205	QFAAD	Mung bean	• Hydroxyl radical scavenging activity<br>• Superoxide radical scavenging activity<br>• Ferric reducing antioxidant power<br>• Metal chelating activity	(Sonklin et al., 2021)
206	QLPLL	Cherry (*Prunus cerasus*)	Antioxidant	(García et al., 2015)
207	QREKK	Chickpea seeds	• Reducing power activity<br>• DPPH radical scavenging activity<br>• Inhibit peroxyl radical-induced DCFH oxidation to DCF in Caco-2 cells	(Torres-Fuentes et al., 2015)
208	QTPRY	Cottonseed	• DPPH radical scavenging activity<br>• ABTS radical scavenging activity<br>• hydroxyl radical scavenging activity<br>• Fe^{2+} chelating activities	(Wang et al., 2021)
209	QWDRQ	Cottonseed	• DPPH radical scavenging activity<br>• ABTS radical scavenging activity<br>• hydroxyl radical scavenging activity<br>• Fe^{2+} chelating activities	(Wang et al., 2021)
210	RDSHQ	Chickpea seeds	• Reducing power activity<br>• DPPH radical scavenging activity	(Torres-Fuentes et al., 2015)

			• Inhibit peroxyl radical-induced DCFH oxidation to DCF in Caco-2 cells	
211	REPKW	Cottonseed	• DPPH radical scavenging activity<br>• ABTS radical scavenging activity<br>• hydroxyl radical scavenging activity<br>• Fe^{2+} chelating activities	(Wang et al., 2021)
212	RPSYT	Soybean (*Glycine max*)	Antioxidant	(Puchalska, Concepción García and Luisa Marina, 2014)
213	RWAEK	Kidney bean (*Phaseolus vulgaris*)	Antioxidant	(Mojica, Chen and Mejía, 2015)
214	SHNPF	Cottonseed	• DPPH radical scavenging activity<br>• ABTS radical scavenging activity<br>• hydroxyl radical scavenging activity<br>• Fe^{2+} chelating activities	(Wang et al., 2021)
215	TELAL	Cherry (*Prunus cerasus*)	Antioxidant	(García et al., 2015)
216	TPAIS	Chinese chestnut (*Castanea mollissima* Blume)	ABTS radical scavenging activity	(Feng et al., 2018)
217	TPLTR	Wheat bran	It is identified from from <1 kDa protein hydrolysate, which has the highest ORAC values in this study.	(Zou et al., 2020)
218	TSSLP	Pea	• DPPH radical scavenging activity<br>• Hydroxyl radical scavenging activity<br>• Reducing power activity<br>• ABTS radical scavenging activity	(Qin et al., 2020)
219	VFAAL	Chinese cherry seed (*Prunus pseudocerasus* Lindl.)	• Hydroxyl radical scavenging activity<br>• DPPH radical scavenging activity<br>• Superoxide anion radical scavenging activity<br>• ABTS radical cation scavenging activities	(Guo et al., 2015)
220	VPHYN	Chickpea seeds	• Reducing power activity<br>• DPPH radical scavenging activity<br>• Inhibit peroxyl radical-induced DCFH oxidation to DCF in Caco-2 cells	(Torres-Fuentes et al., 2015)
221	VVKCG	Emmer (*Triticum dicoccum*)	DPPH radical scavenging activity	(Babini et al., 2017)
222	YALKG	Chickpea seeds	• Reducing power activity<br>• DPPH radical scavenging activity<br>• Inhibit peroxyl radical-induced DCFH oxidation to DCF in Caco-2 cells	(Torres-Fuentes et al., 2015)
223	YLLLK	Palm Kernel cake	• DPPH radical scavenging activity<br>• Metal chelating activity	(Zarei et al., 2014)
224	YNLRQ	Soybean (*Glycine max*)	Antioxidant	(Puchalska, Concepción García and Luisa Marina, 2014)
225	YRILE	Soybean (*Glycine max*)	• $ABTS^{\cdot+}$ scavenging activity<br>• Peroxyl radical scavenging capacity<br>• Increased No_2^- production	(Amigo-Benavent et al., 2014)

			• Decrease in TBARS levels (lipid peroxidation)<br>• Increase in H-Caco-2 cells of MnSOD and CAT activity	
226	AIILHQ	Wheat (*Triticum aestivum*)	Antioxidant	(Babini et al., 2017)
227	ALPDEV	Peach seed (*Prunus persica* (L.) *Batsch*)	It is identified from the whole Thermolysin extract, and this extract has<br>• ABTS radical scavenging activity<br>• hydroxyl radical scavenging activity<br>• lipid peroxidation inhibition activity<br>• ferric reducing antioxidant power	(Vásquez-Villanueva, Marina and García, 2016)
228	AMFVPH	Pea	• Superoxide anion and hydroxyl radicals scavenging activity<br>• ABTS radical scavenging activity.<br>• DPPH radical scavenging activity<br>• Lipid peroxidation inhibition	(Babini et al., 2017)
229	ANSLLN	Soybean (*Glycine max*)	Antioxidant	(Puchalska, Concepción García and Luisa Marina, 2014)
230	AQIPQQ	Wheat (*Triticum aestivum*)	Antioxidant	(Suetsuna and Chen, 2002)
231	DVFNPR	Cottonseed	• DPPH radical scavenging activity<br>• ABTS radical scavenging activity<br>• hydroxyl radical scavenging activity<br>• Fe^{2+} chelating activities	(Wang et al., 2021)
232	EDVDFR	Fennel seed (*Foeniculum vulgare*)	No ABTS radical scavenging activity but it can inhibit albumin protein denaturation (which have the possible applications in health-promoting supplement to alleviate aging-related diseases).	(Mohana Dass, Chai and Wong, 2019)
233	EEGGSV	Soybean (*Glycine max*)	Antioxidant	(Puchalska, Concepción García and Luisa Marina, 2014)
234	EGEEEE	Chickpea seeds	• Reducing power activity<br>• DPPH radical scavenging activity<br>• Inhibit peroxyl radical-induced DCFH oxidation to DCF in Caco-2 cells	(Torres-Fuentes et al., 2015)
235	FDGEVK	Olive seeds (*Olea europaea*)	Antioxidant	(Esteve, Marina and García, 2015)
236	FETLPF	Semen cassiae (*Cassia obtusifolia* L.)	ABTS radical scavenging activity	(Chai et al., 2019)
237	FLQPHQ	Wheat (*Triticum aestivum*)	DPPH radical scavenging activity	(Babini et al., 2017)
238	FPELLI	Chinese cherry seed (*Prunus pseudocerasus* Lindl.)	• Hydroxyl radical scavenging activity<br>• DPPH radical scavenging activity<br>• Superoxide anion radical scavenging activity	(Guo et al., 2015)

			• ABTS radical cation scavenging activities	
239	FVPTQQ	Peach seed (*Prunus persica* (L.) *Batsch*)	It is identified from the whole Thermolysin extract, and this extract has<br>• ABTS radical scavenging activity<br>• hydroxyl radical scavenging activity<br>• lipid peroxidation inhibition activity<br>• ferric reducing antioxidant power	(Vásquez-Villanueva, Marina and García, 2016)
240	GKGIFG	Soybean (*Glycine max*)	Antioxidant	(Puchalska, Concepción García and Luisa Marina, 2014)
241	HQMPKP	Pea	• DPPH radical scavenging activity<br>• Hydroxyl radical scavenging activity<br>• Reducing power activity<br>• ABTS radical scavenging activity	(Qin et al., 2020)
242	IKATSN	Chickpea seeds	• Reducing power activity<br>• DPPH radical scavenging activity<br>• Inhibit peroxyl radical-induced DCFH oxidation to DCF in Caco-2 cells	(Torres-Fuentes et al., 2015)
243	KELEEK	Watermelon seed (*Citrullus lanatus*)	• ABTS radical scavenging activity<br>• DPPH radical scavenging activity<br>• High ORAC value<br>• Increase cell viability of H_2O_2-treated HepG2 cell	(Wen et al., 2020)
244	KGVITQ	Soybean (*Glycine max*)	Antioxidant	(Puchalska, Concepción García and Luisa Marina, 2014)
245	KIIIYN	Coconut cake albumin	• Hydroxyl radical scavenging activity<br>• Iron (II) ion chelating activity<br>• Superoxide radical scavenging activity.<br>• Protect EA.hy926 cells against H_2O_2 induced damage	(Zheng, Li and Li, 2019)
246	KILIYG	Coconut cake albumin	• Hydroxyl radical scavenging activity<br>• Iron (II) ion chelating activity<br>• Superoxide radical scavenging activity.<br>• Protect EA.hy926 cells against H_2O_2 induced damage	(Zheng, Li and Li, 2019)
247	KMRDNL	Semen cassiae (*Cassia obtusifolia* L.)	ABTS radical scavenging activity	(Chai et al., 2019)
248	KSKVII	Emmer (*Triticum dicoccum*)	DPPH radical scavenging activity	(Babini et al., 2017)
249	LGTFQN	Foxtail millet (*Setaria italica*)	Antioxidant	(Amadou et al., 2013)
250	LHALLL	Foxtail millet (*Setaria italica*)	Antioxidant	(Amadou et al., 2013)
251	LLPHHH	Soybean (*Glycine max*)	Antioxidant	(Chen et al., 1996)
252	LPHFNS	Chickpea seeds	• Reducing power activity<br>• DPPH radical scavenging activity	(Torres-Fuentes et al., 2015)

			• Inhibit peroxyl radical-induced DCFH oxidation to DCF in Caco-2 cells	
253	LRTLPM	Barley (*Hordeum vulgare*)	Antioxidant	(Xia et al., 2012)
254	LTPTSN	Cherry (*Prunus cerasus*)	Antioxidant	(Esteve, Marina and García, 2015)
		Olive seeds (*Olea europaea*)		(García et al., 2015)
255	LTTLDS	Oil palm kernel (*Elaeis guineensis Jacq.*)	• Antioxidant capacity (AC) of ABTS radical scavenging and β-carotene-linoleate bleaching (BCB) increases with increased protein content<br>• AC of Ferric Reducing Antioxidant Power decreases with increase protein content	(Chang et al., 2015)
256	LYTPHW	Peach seed (*Prunus persica* (L.) *Batsch*)	It is identified from the whole Thermolysin extract, and this extract has<br>• ABTS radical scavenging activity<br>• hydroxyl radical scavenging activity<br>• lipid peroxidation inhibition activity<br>• ferric reducing antioxidant power	(Vásquez-Villanueva, Marina and García, 2016)
257	MAKKSA	Emmer (*Triticum dicoccum*)	DPPH radical scavenging activity	(Babini et al., 2017)
258	NLGDPS	Cherry (*Prunus cerasus*)	Antioxidant	(García et al., 2015)
259	NLGNPE	Cherry (*Prunus cerasus*)	Antioxidant	(García et al., 2015)
260	PCPVAA	Wheat (*Triticum aestivum*)	DPPH scavenging activity	(Babini et al., 2017)
261	PNHPEL	Chickpea seeds	• Reducing power activity<br>• DPPH radical scavenging activity<br>• Inhibit peroxyl radical-induced DCFH oxidation to DCF in Caco-2 cells	(Torres-Fuentes et al., 2015)
262	PPHMLP	Pinto beans (*Phaseolus vulgaris* cv. Pinto)	It is identified from <3 peptide fraction, which has<br>• ABTS radical scavenging activity<br>• FRAP	(Ngoh and Gan, 2016)
263	PPKIYP	Pea	• DPPH radical scavenging activity<br>• Hydroxyl radical scavenging activity<br>• Reducing power activity<br>• ABTS radical scavenging activity	(Qin et al., 2020)
264	PPMHLP	Pinto beans (*Phaseolus vulgaris* cv. Pinto)	It is identified from <3 peptide fraction, which has<br>• ABTS radical scavenging activity<br>• FRAP	(Ngoh and Gan, 2016)
265	PVETVR	Feng Dan seed (*Paeonia ostia*)	• ABTS radical scavenging activity<br>• Hydroxyl radical scavenging activity	(Wang et al., 2019)

266	QEPLLR	Feng Dan seed (*Paeonia ostia*)	• ABTS radical scavenging activity<br>• Hydroxyl radical scavenging activity	(Wang et al., 2019)
267	QNGRFY	Cottonseed	• DPPH radical scavenging activity<br>• ABTS radical scavenging activity<br>• hydroxyl radical scavenging activity<br>• Fe^{2+} chelating activities	(Wang et al., 2021)
268	QQPQPW	Corn gluten	• DPPH radical scavenging activity<br>• Hydroxyl radical scavenging activity<br>• ABTS radical scavenging activity	(Wang et al., 2014)
269	QRNNPY	Cottonseed	• DPPH radical scavenging activity<br>• ABTS radical scavenging activity<br>• hydroxyl radical scavenging activity<br>• Fe^{2+} chelating activities	(Wang et al., 2021)
270	RDPEER	Watermelon seed (*Citrullus lanatus*)	• ABTS radical scavenging activity<br>• DPPH radical scavenging activity<br>• High ORAC value<br>• Increase cell viability of H_2O_2-treated HepG2 cell<br>• Reduce ROS production in dose-dependent way<br>• Increase activity of SOD, GSH-Px, and CAT<br>• Decrease malondialdehyde (MDA) activity	(Wen et al., 2020)
271	RDPIYS	Soybean (*Glycine max*)	Antioxidant	(Puchalska, Concepción García and Luisa Marina, 2014)
272	RNENEQ	Chickpea seeds	• Reducing power activity<br>• DPPH radical scavenging activity<br>• Inhibit peroxyl radical-induced DCFH oxidation to DCF in Caco-2 cells	(Torres-Fuentes et al., 2015)
273	RNPIYS	Soybean (*Glycine max*)	Antioxidant	(Puchalska, Concepción García and Luisa Marina, 2014)
274	SGYYMH	Foxtail millet (*Setaria italica*)	Antioxidant	(Amadou et al., 2013)
275	SPYCYG	Kidney bean (*Phaseolus vulgaris*)	Antioxidant	(Mojica, Chen and Mejía, 2015)
276	SSHWSS	Cottonseed	• DPPH radical scavenging activity<br>• ABTS radical scavenging activity<br>• hydroxyl radical scavenging activity<br>• Fe^{2+} chelating activities	(Wang et al., 2021)
277	SVNVPL	Barley (*Hordeum vulgare*)	Antioxidant	(Xia et al., 2012)
278	TGRGAP	Pea	• DPPH radical scavenging activity<br>• Hydroxyl radical scavenging activity<br>• Reducing power activity<br>• ABTS radical scavenging activity	(Qin et al., 2020)
279	VEGGLS	Soybean (*Glycine max*)	Antioxidant	(Puchalska, Concepción García and Luisa Marina, 2014)
280	VGPWQK	Jackfruit seed	ABTS radical scavenging activity	(Chai et al., 2021)

		(Artocarpus heterophyllus)		
281	VLLHLK	Emmer (*Triticum dicoccum*)	Antioxidant	(Babini et al., 2017)
282	VPLSPT	Olive seeds (*Olea europaea*)	Antioxidant	(Esteve, Marina and García, 2015)
283	VPVTST	Oil palm kernel (*Elaeis guineensis Jacq.*)	• Antioxidant capacity (AC) of ABTS radical scavenging and β-carotene-linoleate bleaching (BCB) increases with increased protein content<br>• AC of Ferric Reducing Antioxidant Power decreases with increase protein content	(Chang et al., 2015)
284	VSAFLA	Chinese chestnut (*Castanea mollissima* Blume)	ABTS radical scavenging activity	(Feng et al., 2018)
285	VSPHLP	Cottonseed	• DPPH radical scavenging activity<br>• ABTS radical scavenging activity<br>• hydroxyl radical scavenging activity<br>• Fe^{2+} chelating activities	(Wang et al., 2021)
286	VVVVPH	Olive seeds (*Olea europaea*)	Antioxidant	(Esteve, Marina and García, 2015)
287	WDTRGQ	Cottonseed	• DPPH radical scavenging activity<br>• ABTS radical scavenging activity<br>• Hydroxyl radical scavenging activity<br>• Fe^{2+} chelating activities	(Wang et al., 2021)
288	WNMNAH	Cottonseed	• DPPH radical scavenging activity<br>• ABTS radical scavenging activity<br>• Hydroxyl radical scavenging activity<br>• Fe^{2+} chelating activities	(Wang et al., 2021)
289	YRILEF	Soybean (*Glycine max*)	• ABTS radical scavenging activity<br>• Peroxyl radical scavenging capacity<br>• Increased No_2^- production<br>• Decrease in TBARS levels (lipid peroxidation)<br>• Increase in H-Caco-2 cells of MnSOD and CAT activity	(Amigo-Benavent et al., 2014)
290	ADLYNPR	Pea	• DPPH radical scavenging activity<br>• Hydroxyl radical scavenging activity	(Ding et al., 2020)
291	ADVFNPR	Oil palm kernel (*Elaeis guineensis Jacq.*)	• ABTS radical-scavenging activity<br>• Superoxide radical scavenging activity	(Zheng, Li and Zhang, 2017)
292	AEHGSLH	Chickpea seeds	• Reducing power activity<br>• DPPH radical scavenging activity<br>• Inhibit peroxyl radical-induced DCFH oxidation to DCF in Caco-2 cells	(Torres-Fuentes et al., 2015)
293	AEYVRLY	Garden pea (*Pisum sativum*)	Antioxidant	(Babini et al., 2017)
294	AIILHQQ	Wheat (*Triticum aestivum*)	Antioxidant	(Babini et al., 2017)
295	ALEPDHR	Chickpea (*Cicer arietinum*)	Antioxidant	(Torres-Fuentes et al., 2015)
296	APGAGVY	Wheat	Antioxidant	(Matsui, Li and Osajima, 1999)

		(*Triticum aestivum*)		
		Olive seeds (*Olea europaea*)		(Esteve, Marina and García, 2015)
297	CGFPGHC	Purple wheat bran (*Triticum aestivum* L.)	• Superoxide anion radical scavenging activity<br>• Trolox equivalent antioxidant capacity	(Zhao, Zhao and Lu, 2020)
298	CSQAPLA	Corn gluten	• DPPH radical scavenging activity<br>• Superoxide anion radical scavenging activity<br>• Reducing power	(Jin et al., 2016)
299	DAQEFKR	Kamut	• Inhibition of linoleic peroxidation<br>• DPPH radical scavenging activity	(Coda et al., 2012)
300	DFYNPKA	Soybean (*Glycine max*)	Antioxidant	(Puchalska, Concepción García and Luisa Marina, 2014)
301	ESGAVTE	Cherry (*Prunus cerasus*)	Antioxidant	(García et al., 2015)
302	FDAVGVK	Cherry (*Prunus cerasus*)	Antioxidant	(García et al., 2015)
303	FGPEMEQ	Cherry (*Prunus cerasus*)	Antioxidant	(García et al., 2015)
304	FLVPPQE	Soybean (*Glycine max*)	Antioxidant	(Puchalska, Concepción García and Luisa Marina, 2014)
305	FNVPATK	Mung bean (*Vigna radiata* (L.) Wilczek	• ABTS radical scavenging activity<br>• FRAP<br>• Metal chelating activity	(Lapsongphon and Yongsawatdigul, 2013)
306	FRDEHKK	Rice (*Oryza sativa*)	Antioxidant	(Zhang et al., 2010)
307	GANSLLN	Soybean (*Glycine max*)	Antioxidant	(Puchalska, Concepción García and Luisa Marina, 2014)
308	GSIFVPH	Cottonseed	• DPPH radical scavenging activity<br>• ABTS radical scavenging activity<br>• hydroxyl radical scavenging activity<br>• Fe^{2+} chelating activities	(Wang et al., 2021)
309	GVGLFVR	Mung bean (*Vigna radiata* (L.) Wilczek	• ABTS radical scavenging activity<br>• FRAP<br>• Metal chelating activity	(Lapsongphon and Yongsawatdigul, 2013)
310	HGDPEER	Lentil seeds (*Lens culinaris* L. var. Castellana)	• ORAC<br>• Inhibit ROS generation	(Bautista-Expósito et al., 2018)
311	HHQDPPW	Cottonseed	• DPPH radical scavenging activity<br>• ABTS radical scavenging activity<br>• hydroxyl radical scavenging activity<br>• Fe^{2+} chelating activities	(Wang et al., 2021)
312	HNVAMER	Mung bean (*Vigna radiata* (L.) Wilczek	• ABTS radical scavenging activity<br>• FRAP<br>• Metal chelating activity	(Lapsongphon and Yongsawatdigul, 2013)
313	HQNIGSS	Chickpea seeds	• Reducing power activity<br>• DPPH radical scavenging activity<br>• Inhibit peroxyl radical-induced DCFH oxidation to DCF in Caco-2 cells	(Torres-Fuentes et al., 2015)
314	HQTSEFK	Sorghum (*Sorghum bicolor*)	Antioxidant	(Agrawal, Joshi and Gupta, 2017)

315	HVAGTVA	Olive seeds (*Olea europaea*)	Antioxidant	(Esteve, Marina and García, 2015)
316	IILHQQQ	Wheat (*Triticum aestivum*)	Antioxidant	(Babini et al., 2017)
317	IPSEVLA	Soybean (*Glycine max*)	Antioxidant	(Puchalska, Concepción García and Luisa Marina, 2014)
318	IPSEVLS	Soybean (*Glycine max*)	Antioxidant	(Puchalska, Concepción García and Luisa Marina, 2014)
319	KAQYPYV	Coconut cake albumin	• Hydroxyl radical scavenging activity<br>• Iron (II) ion chelating activity<br>• Superoxide radical scavenging activity.<br>• Protect EA.hy926 cells against H_2O_2 induced damage	(Zheng, Li and Li, 2019)
320	KNPQLQD	Chickpea seeds	• Reducing power activity<br>• DPPH radical scavenging activity<br>• Inhibit peroxyl radical-induced DCFH oxidation to DCF in Caco-2 cells	(Torres-Fuentes et al., 2015)
321	LCLAALP	Emmer (*Triticum dicoccum*)	Antioxidant	(Babini et al., 2017)
322	LDDDGRL	Watermelon seed (*Citrullus lanatus*)	• ABTS radical scavenging activity<br>• DPPH radical scavenging activity<br>• High ORAC value<br>• Increase cell viability of H_2O_2-treated HepG2 cell	(Wen et al., 2020)
323	LDESKRF	Semen cassiae (*Cassia obtusifolia* L.)	ABTS radical scavenging activity	(Chai et al., 2019)
324	LDLVKPQ	Hemp seed (*Cannabis sativa* L.)	• ABTS radical scavenging activity<br>• Fe^{2+}-chelating activity<br>• $^{\bullet}OH$ scavenging activity<br>• Causes dose-dependent increase in viability of H_2O_2-treated $HepG_2$ cells<br>• Inhibit ROS generation in H_2O_2-treated $HepG_2$ cells<br>• Increase activity of superoxide dismutase (SOD), catalase (CAT) and glutathione peroxidase (GSH-Px)	(Gao et al., 2021)
325	LLPGANH	Cherry (*Prunus cerasus*)	Antioxidant	(García et al., 2015)
326	LLPHLRR	Mung bean (*Vigna radiata* (L.) Wilczek	• ABTS radical scavenging activity<br>• FRAP<br>• Metal chelating activity	(Lapsongphon and Yongsawatdigul, 2013)
327	LPAAHKA	Golden melon seed (*Cucurbita pepo* L. var. medullosa Alef)	Ferric reducing power activity	(Chen et al., 2021)
328	NQNGRFY	Cottonseed	• DPPH radical scavenging activity<br>• ABTS radical scavenging activity<br>• hydroxyl radical scavenging activity<br>• Fe^{2+} chelating activities	(Wang et al., 2021)

329	NTGNLLG	Soybean (*Glycine max*)	Antioxidant	(Puchalska, Concepción García and Luisa Marina, 2014)
330	PQIPEQF	Barley (*Hordeum vulgare*)	Antioxidant	(Xia et al., 2012)
331	RAILTLV	Mung bean	• DPPH radical scavenging activity<br>• Hydroxyl radical scavenging activity<br>• Superoxide radical scavenging activity<br>• Metal chelating activity<br>• Ferric reducing antioxidant power	(Chunkao et al., 2020)
332	RPNYTDA	Rice (*Oryza sativa*)	Antioxidant	(Yan et al., 2015)
333	SAEHGSL	Chickpea seeds	• Reducing power activity<br>• DPPH radical scavenging activity<br>• Inhibit peroxyl radical-induced DCFH oxidation to DCF in Caco-2 cells	(Torres-Fuentes et al., 2015)
334	SGVVPGY	Mung bean (*Vigna radiata* (L.) Wilczek	• ABTS radical scavenging activity<br>• FRAP<br>• Metal chelating activity	(Lapsongphon and Yongsawatdigul, 2013)
335	SMRKPPG	Peony seed (*Paeonia suffruticosa* Andr.)	• DPPH radical scavenging activity<br>• ABTS radical scavenging activity<br>• Hydroxyl radical scavenging activity<br>• Fe^{2+} chelating activity<br>• Reducing power activity	(F, Zhang et al., 2019)
336	SSSSPDI	Chickpea seeds	• Reducing power activity<br>• DPPH radical scavenging activity<br>• Inhibit peroxyl radical-induced DCFH oxidation to DCF in Caco-2 cells	(Torres-Fuentes et al., 2015)
337	TIIPLPV	Soybean (*Glycine max*)	Antioxidant	(Jiménez-Escrig et al., 2009)
338	VGAPSVS	Olive seeds (*Olea europaea*)	Antioxidant	(Esteve, Marina and García, 2015)
339	VGPLSPT	Olive seeds (*Olea europaea*)	Antioxidant	(Esteve, Marina and García, 2015)
340	VIPAGYP	Soybean (*Glycine max*)	Antioxidant	(Chen, Muramoto and Yamauchi, 1995)
341	VLRPPLS	Feng Dan seed (*Paeonia ostia*)	• ABTS radical scavenging activity<br>• Hydroxyl radical scavenging activity	(Wang et al., 2019)
342	VVALALI	Brown rice	• ABTS radical scavenging activity<br>• Cu^{2+} chelating activity	(Selamassakul et al., 2018)
343	YGNWLYK	Kidney bean (*Phaseolus vulgaris*)	Antioxidant	(Mojica, Chen and Mejía, 2015)
344	YGPQQQE	Cherry (*Prunus cerasus*)	Antioxidant	(García et al., 2015)
345	AHAAGYGG	Golden melon seed (*Cucurbita pepo* L. var. medullosa Alef)	Ferric reducing power activity	(Chen et al., 2021)
346	AIILHQQQ	Wheat (*Triticum aestivum*)	Antioxidant	(Babini et al., 2017)
347	AIRQGDVF	Rice (*Oryza sativa*)	Antioxidant	(Adebiyi et al., 2008)

348	ALEDVVEK	Fennel seed (*Foeniculum vulgare*)	No ABTS radical scavenging activity but it can inhibit albumin protein denaturation (which have the possible applications in health-promoting supplement to alleviate aging-related diseases).	(Mohana Dass, Chai and Wong, 2019)
349	AQKIPAGT	Mung bean	• DPPH radical scavenging activity<br>• Hydroxyl radical scavenging activity<br>• Superoxide radical scavenging activity<br>• Metal chelating activity<br>• Ferric reducing antioxidant power	(Chunkao et al., 2020)
350	ATAFGLMK	Lentil seeds (*Lens culinaris* L. var. Castellana)	• ORAC<br>• Inhibit ROS generation	(Bautista-Expósito et al., 2018)
351	AVKPEPAR	Mung bean (*Vigna radiata* (L.) Wilczek)	• ABTS radical scavenging activity<br>• FRAP<br>• Metal chelating activity	(Lapsongphon and Yongsawatdigul, 2013)
352	CAGVSAIR	Oil palm kernel (*Elaeis guineensis Jacq.*)	• ABTS radical scavenging activity<br>• Superoxide radical scavenging activity	(Zheng, Li and Zhang, 2017)
353	DAAGRLQE	Watermelon seed (*Citrullus lanatus*)	• ABTS radical scavenging activity<br>• DPPH radical scavenging activity<br>• High ORAC value<br>• Increase cell viability of H_2O_2-treated $HepG_2$ cell	(Wen et al., 2020; Wen et al., 2021)
354	DGDPLLDQ	Cherry (*Prunus cerasus*)	Antioxidant	(García et al., 2015)
355	DLPVLRWL	Lentil seeds (*Lens culinaris* var. Castellana)	• Identified from S2 hydrolysate (which has the highest ORAC values)<br>• Peptides identified from lentil hydrolysates produced by HP-assisted proteolysis using Savinase at 300 MPa. Highest ORAC values were observed	(Garcia-Mora et al., 2015)
356	DNIPIVIR	Whole wheat	• Inhibition of linoleic peroxidation<br>• DPPH radical scavenging activity	(Coda et al., 2012)
357	EALEAMFL	Spelt	• Inhibition of linoleic peroxidation<br>• DPPH radical scavenging activity<br>• High hydrophobicity, access hydrophobic radical species	(Coda et al., 2012)
358	EGGLLLPH	Lentil seeds (*Lens culinaris* var. Castellana)	Identified from S2 hydrolysate (which has the highest ORAC values).	(Garcia-Mora et al., 2014)
359	ESDVVSDL	Brown rice	Identified from F4 fraction, which has higher<br>• DPPH scavenging activity<br>• ABTS scavenging activity<br>• hydroxyl scavenging activity	(Selamassakul et al., 2020)
360	FPLEMMPF	Maize (*Zea mays*)	Antioxidant	(Zheng et al., 2006)
361	GHSVIYVQ	Tomato seed (*Solanum lycopersicum*)	• DPPH radical scavenging activity<br>• Phosphomolybdate reducing activity	(Meshginfar et al., 2018)
362	GIEEQQQR	Soybean	• ABTS radical scavenging activity<br>• Peroxyl radical scavenging capacity<br>• Increased No_2^- production<br>• Decrease in TBARS levels (lipid peroxidation)	(Amigo-Benavent et al., 2014)

			• Increase in H-Caco-2 cells of MnSOD and CAT activity	
363	GLLLPHYN	Tomato seed (*Solanum lycopersicum*)	• DPPH radical scavenging activity<br>• Phosphomolybdate reducing activity	(Meshginfar et al., 2018)
364	GSGVGGAK	Brown rice	Identified from F4 fraction, which has higher<br>• DPPH scavenging activity<br>• ABTS scavenging activity<br>• Hydroxyl scavenging activity	(Selamassakul et al., 2020)
365	GTFLQPHQ	Garden pea (*Pisum sativum*)	Antioxidant	(Babini et al., 2017)
366	HPVPPKKK	Spelt	• Inhibition of linoleic peroxidation<br>• DPPH radical scavenging activity<br>• His residue acts as metal ion chelator	(Coda et al., 2012)
367	IIPAGHPV	Chickpea seeds	• Reducing power activity<br>• DPPH radical scavenging activity<br>• Inhibit peroxyl radical-induced DCFH oxidation to DCF in Caco-2 cells	(Torres-Fuentes et al., 2015)
368	ILAGPTTI	Mung bean	• Hydroxyl radical scavenging activity<br>• Superoxide radical scavenging activity<br>• Metal chelating activity<br>• Ferric reducing antioxidant power	(Chunkao et al., 2020)
369	INDKYVLL	Lentil seeds (*Lens culinaris* var. Castellana)	Identified from S2 hydrolysate (which has the highest ORAC values).	(Garcia-Mora et al., 2014)
370	ITFAAYRR	Spelt	• Inhibition of linoleic peroxidation<br>• DPPH radical scavenging activity<br>• Show highest activity	(Coda et al., 2012)
371	KHDRGDEF	Rice (*Oryza sativa*)	Antioxidant	(Zhang et al., 2010)
372	KHNRGDEF	Rice (*Oryza sativa*)	Antioxidant	(Zhang et al., 2009)
373	KKGVLGLA	Mung bean	• DPPH radical scavenging activity<br>• Hydroxyl radical scavenging activity<br>• Superoxide radical scavenging activity<br>• Metal chelating activity<br>• Ferric reducing antioxidant power	(Chunkao et al., 2020)
374	LAGNHEQE	Chickpea seeds	• Reducing power activity<br>• DPPH radical scavenging activity<br>• Inhibit peroxyl radical-induced DCFH oxidation to DCF in Caco-2 cells	(Torres-Fuentes et al., 2015)
375	LAGNPENE	Cherry (*Prunus cerasus*)	Antioxidant	(García et al., 2015)
376	LAGNPQDE	Cherry (*Prunus cerasus*)	Antioxidant	(García et al., 2015)
377	LDLPVLRW	Pea	• Superoxide anion and hydroxyl radicals scavenging activity.<br>• ABTS radical scavenging activity.<br>• DPPH radical scavenging activity<br>• Lipid peroxidation inhibition	(Babini et al., 2017)
378	LPQHIDAD	Chickpea seeds	• Reducing power activity<br>• DPPH radical scavenging activity	(Torres-Fuentes et al., 2015)

			• Inhibit peroxyl radical-induced DCFH oxidation to DCF in Caco-2 cells	
379	LPQHTDAD	Chickpea seeds	• Reducing power activity<br>• DPPH radical scavenging activity<br>• Inhibit peroxyl radical-induced DCFH oxidation to DCF in Caco-2 cells	(Torres-Fuentes et al., 2015)
380	LVYIIQGR	Oil palm kernel (*Elaeis guineensis Jacq.*)	• ABTS radical scavenging activity<br>• Superoxide radical scavenging activity<br>• Has highest antioxidant activity among the four peptides discovered	(Zheng, Li and Zhang, 2017)
381	NGDPLLDQ	Cherry (*Prunus cerasus*)	Antioxidant	(García et al., 2015)
382	NIEDPYRA	Lotus seed (*Nelumbo nucifera* Gaertn.)	• DPPH radical scavenging activity<br>• ABTS radical scavenging activity<br>• H_2O_2 scavenging activity<br>• Reducing power activity	(Yu et al., 2021)
383	NSGPLVNP	Soybean (*Glycine max*)	Antioxidant	(Puchalska, Concepción García and Luisa Marina, 2014)
384	PLPLHMLP	Pinto beans (*Phaseolus vulgaris* cv. Pinto)	Identified from <3 peptide fraction, which has<br>• ABTS radical scavenging activity<br>• FRAP	(Ngoh and Gan, 2016)
385	PLPPHMLP	Pinto beans (*Phaseolus vulgaris* cv. Pinto)	Identified from <3 peptide fraction, which has<br>• ABTS radical scavenging activity<br>• FRAP	(Ngoh and Gan, 2016)
386	QQRQQQGL	Walnut meal (*Juglans regia* L.)	• Hydroxyl radical scavenging activity<br>• Increase viability of SH-SY5Y cells that are treated with H_2O_2<br>• Reduce intracellular ROS	(Sheng et al., 2019)
387	QTFPHQPQ	Emmer	• DPPH scavenging activity	(Babini et al., 2017)
388	RDRHQKIG	Sesame seed (*Sesamum indicum* L.)	• DPPH radical scavenging activity<br>• ABTS radical scavenging activity	(Lu et al., 2019)
389	SAEHGSLH	Chickpea (*Cicer arietinum*)	Antioxidant	(Torres-Fuentes et al., 2015)
390	TDRHQKLR	Sesame seed (*Sesamum indicum* L.)	• DPPH radical scavenging activity<br>• ABTS radical scavenging activity	(Lu et al., 2019)
391	TRTGDPFF	Rice (*Oryza sativa*)	Antioxidant	(Yan et al., 2015)
392	TSQLLSDQ	Rice (*Oryza sativa*)	Antioxidant	(Yan et al., 2015)
393	TVFDGELR	Oil palm kernel (*Elaeis guineensis Jacq.*)	• Antioxidant capacity (AC) of ABTS radical scavenging and β-carotene-linoleate bleaching (BCB) increases with increased protein content<br>• AC of Ferric Reducing Antioxidant Power decreases with increase protein content	(Chang et al., 2015)
394	VAITLTMK	Sorghum (*Sorghum bicolor*)	Antioxidant	(Agrawal, Joshi and Gupta, 2017)
395	VLPPQQQY	Wheat	Antioxidant	(Babini et al., 2017)

		(*Triticum aestivum*)		
396	VNNDDRDS	Soybean	• ABTS radical scavenging activity<br>• Peroxyl radical scavenging capacity<br>• Increased No_2^- production<br>• Decrease in TBARS levels (lipid peroxidation)<br>• Increase in H-Caco-2 cells of MnSOD and CAT activity	(Amigo-Benavent et al., 2014)
397	VNPHDHQN	Soybean (*Glycine max*)	Antioxidant	(Chen, Muramoto and Yamauchi, 1995)
398	VPPSKLSP	Sorghum (*Sorghum bicolor*)	Antioxidant	(Agrawal, Joshi and Gupta, 2017)
399	VSKSVLVK	Sorghum (*Sorghum bicolor*)	Antioxidant	(Agrawal, Joshi and Gupta, 2017)
400	VTYDYYKN	Cherry (*Prunus cerasus*)	Antioxidant	(García et al., 2015)
401	VVGGDGDV	Oil palm kernel (*Elaeis guineensis Jacq.*)	• Antioxidant capacity (AC) of ABTS radical scavenging and β-carotene-linoleate bleaching (BCB) increases with increased protein content.<br>• AC of Ferric Reducing Antioxidant Power decreases with increase protein content	(Chang et al., 2015)
402	VVRPPFSQ	Tomato seed (*Solanum lycopersicum*)	• DPPH radical scavenging activity<br>• Phosphomolybdate reducing activity	(Meshginfar et al., 2018)
403	YGRDEISV	Hemp seed (*Cannabis sativa* L.)	• ABTS radical scavenging activity<br>• Fe^{2+}-chelating activity<br>• $^{\bullet}OH$ scavenging activity<br>• Causes dose-dependent increase in viability of H_2O_2-treated $HepG_2$ cells<br>• Inhibit ROS generation in H_2O_2-treated $HepG_2$ cells<br>• Increase activity of superoxide dismutase (SOD), catalase (CAT) and glutathione peroxidase (GSH-Px)	(Gao et al., 2021)
404	YPKLAPNE	Corn gluten	• DPPH radical scavenging activity<br>• Superoxide anion radical scavenging activity<br>• Reducing power	(Jin et al., 2016)
405	YPQLLPNE	Corn gluten	• DPPH radical scavenging activity<br>• Superoxide anion radical scavenging activity<br>• Reducing power	(Jin et al., 2016)
406	YRQYPFQQ	Tomato seed (*Solanum lycopersicum*)	• DPPH radical scavenging activity<br>• Phosphomolybdate reducing activity	(Meshginfar et al., 2018)
407	YSNQNGRF	Cottonseed	• DPPH radical scavenging activity<br>• ABTS radical scavenging activity<br>• hydroxyl radical scavenging activity<br>• Fe^{2+} chelating activities	(Wang et al., 2021)
408	YSSPIHIW	Pea	• DPPH radical scavenging activity<br>• Hydroxyl radical scavenging activity	(Ding et al., 2020)

409	ASHGDFRIL	Tomato seed (*Solanum lycopersicum*)	• DPPH radical scavenging activity<br>• Phosphomolybdate reducing activity	(Meshginfar et al., 2018)
410	EFFDVSNEQ	Brown rice	• ABTS radical scavenging activity<br>• Cu^{2+} chelating activity	(Selamassakul et al., 2018)
411	ENLQNYRLL	Garden pea (*Pisum sativum*)	Antioxidant	(Babini et al., 2017)
412	ESTLHLVLR	Emmer (*Triticum dicoccum*)	Antioxidant	(Babini et al., 2017)
413	FGGSGGPGG	Brown rice	Identified from F4 fraction, which has higher<br>• DPPH radical scavenging activity<br>• ABTS radical scavenging activity<br>• Hydroxyl radical scavenging activity	(Selamassakul et al., 2020)
414	FNQLDDEVR	Lotus seed (*Nelumbo nucifera* Gaertn.)	• DPPH radical scavenging activity<br>• ABTS radical scavenging activity<br>• H_2O_2 scavenging activity<br>• Reducing power activity	(Yu et al., 2021)
415	FPFPRPPHQ	Soybean (*Glycine max*)	Antioxidant	(Puchalska, Concepción García and Luisa Marina, 2014)
416	HTQHQFFHG	Tomato seed (*Solanum lycopersicum*)	• DPPH radical scavenging activity<br>• Phosphomolybdate reducing activity	(Meshginfar et al., 2018)
417	HYDSEAILF	Pea protein	• DPPH radical scavenging activity<br>• Hydroxyl (OH) radical scavenging activity	(Ding et al., 2020)
418	LGNTDYAVH	*Ginkgo biloba* seeds (*Ginkgo biloba)*	• DPPH radical scavenging activity<br>• Hydroxyl radical scavenging activity<br>• Superoxide radical scavenging activity	(Wu et al., 2013)
419	LIRHVIQSR	Tomato seed (*Solanum lycopersicum*)	• DPPH radical scavenging activity<br>• Phosphomolybdate reducing activity	(Meshginfar et al., 2018)
420	LLPHHADAD	Soybean	• ABTS radical scavenging activity<br>• Peroxyl radical scavenging capacity<br>• Increased No_2^- production<br>• Decrease in TBARS levels (lipid peroxidation)<br>• Increase in H-Caco-2 cells of MnSOD and CAT activity	(Amigo-Benavent et al., 2014)
421	LMRVLAQLG	Golden melon seed (*Cucurbita pepo* L. var. *medullosa* Alef)	Ferric reducing power activity	(Chen et al., 2021)
422	LQPGQGQQG	Wheat (*Triticum aestivum*)	Antioxidant	(Suetsuna and Chen, 2002)
423	LVNNDDRDS	Soybean	• ABTS radical scavenging activity<br>• Peroxyl radical scavenging capacity<br>• Increased No_2^- production<br>• Decrease in TBARS levels (lipid peroxidation)<br>• Increase in H-Caco-2 cells of MnSOD and CAT activity	(Amigo-Benavent et al., 2014)

424	LVNPHDHQN	Soybean (*Glycine max*)	Antioxidant	(Chen, Muramoto and Yamauchi, 1995)
425	MNDRVNQGE	Sesame seed (*Sesamum indicum* L.)	• DPPH radical scavenging activity<br>• ABTS radical scavenging activity	(Lu et al., 2019)
426	NEDVIVKVS	Lentil seeds (*Lens culinaris* var. Castellana)	Identified from S2 hydrolysate (which has the highest ORAC values).	(Garcia-Mora et al., 2014)
427	NINAHSVVY	Oat bran	Inhibition of linoleic acid peroxidation	(Esfandi et al., 2021)
428	NLDALEPSR	Lotus seed (*Nelumbo nucifera* Gaertn.)	• DPPH radical scavenging activity<br>• ABTS radical scavenging activity<br>• H_2O_2 scavenging activity<br>• Reducing power activity	(Yu et al., 2021)
429	PSYLNTPLL	Tomato seed (*Solanum lycopersicum*)	• DPPH radical scavenging activity<br>• Phosphomolybdate reducing activity	(Meshginfar et al., 2018)
430	SDLDPIRHK	Tomato seed (*Solanum lycopersicum*)	• DPPH radical scavenging activity<br>• Phosphomolybdate reducing activity	(Meshginfar et al., 2018)
431	SDRFTYVAF	Lentil seeds (*Lens culinaris* var. Castellana)	Peptides identified from lentil hydrolysates produced by HP-assisted proteolysis using Savinase at 300 MPa. Highest ORAC values were observed.	(Garcia-Mora et al., 2015)
432	SLDLPVLRW	Lentil seeds (*Lens culinaris* var. Castellana)	Identified from S2 hydrolysate (which has the highest ORAC values).	(Garcia-Mora et al., 2014)
433	SPFWNINAH	Oat bran	Inhibition of linoleic acid peroxidation	(Esfandi et al., 2021)
434	SPQLQNLRD	Soybean	• ABTS radical scavenging activity<br>• Peroxyl radical scavenging capacity<br>• Increased No_2^- production<br>• Decrease in TBARS levels (lipid peroxidation)<br>• Increase in H-Caco-2 cells of MnSOD and CAT activity	(Amigo-Benavent et al., 2014)
435	SSAPGGGRP	Brown rice	• ABTS radical scavenging activity<br>• Cu^{2+} chelating activity	(Selamassakul et al., 2018)
436	SSVGGGSAG	Brown rice	Identified from F4 fraction, which has higher<br>• DPPH radical scavenging activity<br>• ABTS radical scavenging activity<br>• Hydroxyl radical scavenging activity	(Selamassakul et al., 2020)
437	SYPTECRMR	Sesame seed (*Sesamum indicum* L.)	• DPPH radical scavenging activity<br>• ABTS radical scavenging activity	(Lu et al., 2019)
438	VLDTGLAGA	Olive seeds (*Olea europaea*)	Antioxidant	(Esteve, Marina and García, 2015)
439	VLEANPRSF	Rice (*Oryza sativa*)	Antioxidant	(Adebiyi et al., 2008)
440	VLSPPFTGE	Olive seeds (*Olea europaea*)	Antioxidant	(Esteve, Marina and García, 2015)
441	VMTVHNTPY	Wheat (*Triticum aestivum*)	Antioxidant	(Babini et al., 2017)
442	YLAGNHEQE	Chickpea seeds	• Reducing power activity<br>• DPPH radical scavenging activity<br>• Inhibit peroxyl radical-induced DCFH oxidation to DCF in Caco-2 cells	(Torres-Fuentes et al., 2015)

443	AQGTFLQPHQ	Wheat (*Triticum aestivum*)	Antioxidant	(Babini et al., 2017)
444	DLPVLRWLKL	Lentil seeds (*Lens culinaris* var. Castellana)	• Identified from S2 hydrolysate (which has the highest ORAC values)<br>• Peptides identified from lentil hydrolysates produced by HP-assisted proteolysis using Savinase at 300 MPa. Highest ORAC values were observed	(Garcia-Mora et al., 2015)
445	ENENGHIRLL	Lentil seeds (*Lens culinaris* var. Castellana)	Peptides identified from lentil hydrolysates produced by HP-assisted proteolysis using Savinase at 300 MPa. Highest ORAC values were observed.	(Garcia-Mora et al., 2015)
446	ESDLDPIRHK	Tomato seed (*Solanum lycopersicum*)	• DPPH radical scavenging activity<br>• Phosphomolybdate reducing activity	(Meshginfar et al., 2018)
447	FEITPEKNPQ	Soybean	• ABTS radical scavenging activity<br>• Peroxyl radical scavenging capacity<br>• Increased No_2^- production<br>• Decrease in TBARS levels (lipid peroxidation)<br>• Increase in H-Caco-2 cells of MnSOD and CAT activity	(Amigo-Benavent et al., 2014)
448	FGGGGAGAGG	Brown rice	Identified from F4 fraction, which has higher<br>• DPPH radical scavenging activity<br>• ABTS radical scavenging activity<br>• Hydroxyl radical scavenging activity	(Selamassakul et al., 2020)
449	GFAGDDAPRA	Watermelon seed (*Citrullus lanatus*)	• ABTS radical scavenging activity<br>• DPPH radical scavenging activity<br>• High ORAC value<br>• Increase cell viability of H_2O_2-treated $HepG_2$ cell	(Wen et al., 2020; Wen et al., 2021)
450	GHDDDRGEIV	Lotus seed (*Nelumbo nucifera* Gaertn.)	• DPPH scavenging activity<br>• ABTS scavenging activity<br>• H_2O_2 scavenging activity<br>• Reducing power activity	(Yu et al., 2021)
451	GKGEGGGGLA	Brown rice	• ABTS radical scavenging activity<br>• Cu^{2+} chelating activity	(Selamassakul et al., 2018)
452	GLLGKNFTSK	Sorghum (*Sorghum bicolor*)	Antioxidant	(Agrawal, Joshi and Gupta, 2017)
453	GVSLIRHVIQ	Tomato seed (*Solanum lycopersicum*)	• DPPH radical scavenging activity<br>• Phosphomolybdate reducing activity	(Meshginfar et al., 2018)
454	HLPSFSPSPQ	Chickpea seeds	• Reducing power activity<br>• DPPH radical scavenging activity<br>• Inhibit peroxyl radical-induced DCFH oxidation to DCF in Caco-2 cells	(Torres-Fuentes et al., 2015)
455	KQVHPDIGIS	Tomato seed (*Solanum lycopersicum*)	• DPPH radical scavenging activity<br>• Phosphomolybdate reducing activity	(Meshginfar et al., 2018)
456	KTISSEDQPF	Soybean	• ABTS radical scavenging activity<br>• Peroxyl radical scavenging capacity<br>• Increased No_2^- production	(Amigo-Benavent et al., 2014)

			• Decrease in TBARS levels (lipid peroxidation)<br>• Increase in H-Caco-2 cells of MnSOD and CAT activity	
457	LCPVHRAADL	Rye	• Inhibition of linoleic peroxidation<br>• DPPH radical scavenging activity<br>• High hydrophobicity, access hydrophobic radical species	(Coda et al., 2012)
458	LDSCKDYVME	Sorghum (*Sorghum bicolor*)	Antioxidant	(Agrawal, Joshi and Gupta, 2017)
459	LFINDKYVLL	Lentil seeds (*Lens culinaris* var. Castellana)	Identified from S2 hydrolysate (which has the highest ORAC values).	(Garcia-Mora et al., 2014)
460	LGSFLYGYSR	Mung bean (*Vigna radiata* (L.) Wilczek)	• ABTS radical scavenging activity<br>• FRAP<br>• Metal chelating activity	(Lapsongphon and Yongsawatdigul, 2013)
461	LLNDEVKEGQ	Cherry (*Prunus cerasus*)	Antioxidant	(García et al., 2015)
462	LLPHHADADY	Soybean (*Glycine max*)	Antioxidant	(Chen, Muramoto and Yamauchi, 1995)
463	LPWRPATNVF	Palm Kernel cake	• DPPH radical scavenging activity<br>• Metal chelating activity	(Zarei et al., 2014)
464	NILEASYNTR	Pea	• Superoxide anion and hydroxyl radicals scavenging activity<br>• ABTS radical scavenging activity<br>• DPPH radical scavenging activity<br>• Lipid peroxidation inhibition	(Babini et al., 2017)
465	QITEGEDGGG	*Caragana ambigua* seed	• Scavenge superoxide anions<br>• Inhibit rate of linoleic acid oxidation<br>• Delay auto-oxidation of walnut oil	(Jie et al., 2019)
466	QKPFPQQPPF	Barley (*Hordeum vulgare*)	Antioxidant	(Xia et al., 2012)
467	QLNEPDNRLQ	Cherry (*Prunus cerasus*)	Antioxidant	(García et al., 2015)
468	QLPEPDNRLQ	Cherry (*Prunus cerasus*)	Antioxidant	(García et al., 2015)
469	RENIDKPSRA	Sesame seed (*Sesamum indicum* L.)	• DPPH radical scavenging activity<br>• ABTS radical scavenging activity	(Lu et al., 2019)
470	REQIEELRRL	Lentil seeds (*Lens culinaris* var. Castellana)	Peptides identified from lentil hydrolysates produced by HP-assisted proteolysis using Savinase at 300 MPa. Highest ORAC values were observed.	(Garcia-Mora et al., 2015)
471	RLSAEYVRLY	Lentil seeds (*Lens culinaris* var. Castellana)	Identified from S2 hydrolysate (which has the highest ORAC values).	(Garcia-Mora et al., 2014)
472	RQSHFANAQP	Chickpea (*Cicer arietinum* L.)	• DPPH radical scavenging activity<br>• Hydroxyl radical scavenging activity<br>• Reducing power<br>• TEAC	(Kou et al., 2013)
473	RRIEAEAGRL	Lotus seed (*Nelumbo nucifera* Gaertn.)	• DPPH radical scavenging activity<br>• ABTS radical scavenging activity<br>• H_2O_2 scavenging activity<br>• Reducing power activity	(Yu et al., 2021)
474	RSDQDNPFIF	Lentil seeds (*Lens culinaris* var. Castellana)	Peptides identified from lentil hydrolysates produced by HP-assisted proteolysis using Savinase at 300 MPa. Highest ORAC values were observed.	(Garcia-Mora et al., 2015)

475	RVQVVNNNGK	Brown rice	• ABTS radical scavenging activity<br>• Cu^{2+} chelating activity	(Selamassakul et al., 2018)
476	SDQENPFIFK	Lentil seeds (*Lens culinaris* var. Castellana)	• ORAC<br>• Inhibit ROS generation	(Bautista-Expósito et al., 2018)
477	SGHKIPAIGL	Tomato seed (*Solanum lycopersicum*)	• DPPH radical scavenging activity<br>• Phosphomolybdate reducing activity	(Meshginfar et al., 2018)
478	SGPSGGGGAL	Brown rice	Identified from F4 fraction, which has higher<br>• DPPH radical scavenging activity<br>• ABTS radical scavenging activity<br>• Hydroxyl scavenging activity	(Selamassakul et al., 2020)
479	SLDLPVLRWL	Lentil seeds (*Lens culinaris* var. Castellana)	Identified from S2 hydrolysate (which has the highest ORAC values).	(Garcia-Mora et al., 2014)
480	SLPNFHPMPR	Tomato seed (*Solanum lycopersicum*)	• DPPH radical scavenging activity<br>• Phosphomolybdate reducing activity	(Meshginfar et al., 2018)
481	SLVNNDDRDS	Soybean	• ABTS radical scavenging activity<br>• Peroxyl radical scavenging capacity<br>• Increased No_2^- production<br>• Decrease in TBARS levels (lipid peroxidation)<br>• Increase in H-Caco-2 cells of MnSOD and CAT activity	(Amigo-Benavent et al., 2014)
482	SPFWNINAHS	Brown rice	• ABTS radical scavenging activity<br>• Cu^{2+} chelating activity	(Selamassakul et al., 2018)
483	STTTGHLIYK	Emmer (*Triticum dicoccum*)	Antioxidant	(Babini et al., 2017)
484	TETWNPNHPE	Chickpea seeds	• Reducing power activity<br>• DPPH radical scavenging activity<br>• Inhibit peroxyl radical-induced DCFH oxidation to DCF in Caco-2 cells	(Torres-Fuentes et al., 2015)
485	THPDVPGEPT	Tomato seed (*Solanum lycopersicum*)	• DPPH radical scavenging activity<br>• Phosphomolybdate reducing activity	(Meshginfar et al., 2018)
486	TLVNNDDRDS	Soybean	• ABTS radical scavenging activity<br>• Peroxyl radical scavenging capacity<br>• Increased No_2^- production<br>• Decrease in TBARS levels (lipid peroxidation)<br>• Increase in H-Caco-2 cells of MnSOD and CAT activity	(Amigo-Benavent et al., 2014)
487	TMAEYHHQDQ	Brown rice	• ABTS radical scavenging activity<br>• Cu^{2+} chelating activity	(Selamassakul et al., 2018)
488	TSLDLPVLRW	Garden pea (*Pisum sativum*)	Antioxidant	(Babini et al., 2017)
489	TSVEIITSSK	Sorghum (*Sorghum bicolor*)	Antioxidant	(Agrawal, Joshi and Gupta, 2017)
490	VEVGGGARAP	Brown rice	• ABTS radical scavenging activity<br>• Cu^{2+} chelating activity	(Selamassakul et al., 2018)
491	YFDEQNEQFR	Oat bran	Inhibition of linoleic acid peroxidation	(Esfandi et al., 2021)
492	YGIKVGYAIP	Palm Kernel cake	• DPPH radical scavenging activity<br>• Metal chelating activity<br>• High metal chelating activity	(Zarei et al., 2014)

493	YLESQQSSQQ	Peach seed (*Prunus persica* (L.) *Batsch*)	Identified from the whole Thermolysin extract, and this extract has<br>• ABTS radical scavenging activity<br>• hydroxyl radical scavenging activity<br>• lipid peroxidation inhibition activity<br>• ferric reducing antioxidant power	(Vásquez-Villanueva, Marina and García, 2016)
494	YQETSSSSSQ	Brown rice	• ABTS radical scavenging activity<br>• Cu^{2+} chelating activity	(Selamassakul et al., 2018)
495	YSEEQQPSTR	Brown rice	• ABTS radical scavenging activity<br>• Cu^{2+} chelating activity	(Selamassakul et al., 2018)
496	YVVNPDNDEN	Soybean	• ABTS radical scavenging activity<br>• Peroxyl radical scavenging capacity<br>• Increased No_2^- production<br>• Decrease in TBARS levels (lipid peroxidation)<br>• Increase in H-Caco-2 cells of MnSOD and CAT activity	(Amigo-Benavent et al., 2014)
497	YVVNPDNNEN	Soybean	• ABTS radical scavenging activity<br>• Peroxyl radical scavenging capacity<br>• Increased No_2^- production<br>• Decrease in TBARS levels (lipid peroxidation)<br>• Increase in H-Caco-2 cells of MnSOD and CAT activity	(Amigo-Benavent et al., 2014)
498	AGGGGGGVVAG	Brown rice	Identified from F4 fraction, which has higher<br>• DPPH radical scavenging activity<br>• ABTS radical scavenging activity<br>• hydroxyl radical scavenging activity	(Selamassakul et al., 2020)
499	DFNQLDDEVRN	Lotus seed (*Nelumbo nucifera* Gaertn.)	• DPPH radical scavenging activity<br>• ABTS radical scavenging activity<br>• H_2O_2 scavenging activity<br>• Reducing power activity	(Yu et al., 2021)
500	DHHQVYSPGEQ	Rice (*Oryza sativa*)	Antioxidant	(Wei et al., 2007)
501	DMIAVPDGVTH	Lotus seed (*Nelumbo nucifera* Gaertn.)	• DPPH radical scavenging activity<br>• ABTS radical scavenging activity<br>• H_2O_2 scavenging activity<br>• Reducing power activity	(Yu et al., 2021)
502	EFFDVSNELFQ	Brown rice	• $ABTS^{\cdot+}$ scavenging activity<br>• Cu^{2+} chelating activity	(Selamassakul et al., 2018)
503	EHIMPLGQNGR	Chañar seeds (*Geoffroea decorticans*)	• DPPH radical scavenging activity<br>• ABTS radical scavenging activity	(Cotabarren et al., 2021)
504	EPFNLRSRNPI	Soybean	• ABTS radical scavenging activity<br>• Peroxyl radical scavenging capacity<br>• Increased No_2^- production<br>• Decrease in TBARS levels (lipid peroxidation)<br>• Increase in H-Caco-2 cells of MnSOD and CAT activity	(Amigo-Benavent et al., 2014)

505	FLGSFLYGYSR	Mung bean (*Vigna radiata* (L.) Wilczek)	• ABTS radical scavenging activity<br>• FRAP<br>• Metal chelating activity	(Lapsongphon and Yongsawatdigul, 2013)
506	GEQQQQPGMTR	Brown rice	• ABTS radical scavenging activity<br>• Cu^{2+} chelating activity	(Selamassakul et al., 2018)
507	GGGGGAAAAGA	Brown rice	Identified from F4 fraction, which has higher<br>• DPPH scavenging activity<br>• ABTS scavenging activity<br>• hydroxyl scavenging activity	(Selamassakul et al., 2020)
508	GPIYSNEFGKF	Garden pea (*Pisum sativum*)	Antioxidant	(Babini et al., 2017)
509	HIAGKSSIFRA	Brown rice	• ABTS radical scavenging activity<br>• Cu^{2+} chelating activity	(Selamassakul et al., 2018)
510	HNLDTQTESDV	Walnut meal (*Juglans regia* L.)	• Hydroxyl radical scavenging activity<br>• Increase viability of SH-SY5Y cells that are treated with H_2O_2<br>• Reduce intracellular ROS	(Sheng et al., 2019)
511	ISPRILSYNLR	Perilla seed (*Perilla frutescens* var. *japonica* HARA)	• DPPH radical scavenging activity<br>• ABTS radical scavenging activity<br>• Reducing power	(Kim, Liceaga and Yoon, 2019)
512	ITLAIPVNKPG	Soybean	• ABTS radical scavenging activity<br>• Peroxyl radical scavenging capacity<br>• Increased No_2^- production<br>• Decrease in TBARS levels (lipid peroxidation)<br>• Increase in H-Caco-2 cells of MnSOD and CAT activity	(Amigo-Benavent et al., 2014)
513	KPFNLRSRDPI	Soybean	• ABTS radical scavenging activity<br>• Peroxyl radical scavenging capacity<br>• Increased No_2^- production<br>• Decrease in TBARS levels (lipid peroxidation)<br>• Increase in H-Caco-2 cells of MnSOD and CAT activity	(Amigo-Benavent et al., 2014)
514	KTISSEDQPFN	Soybean	• ABTS radical scavenging activity<br>• Peroxyl radical scavenging capacity<br>• Increased No_2^- production<br>• Decrease in TBARS levels (lipid peroxidation)<br>• Increase in H-Caco-2 cells of MnSOD and CAT activity	(Amigo-Benavent et al., 2014)
515	KVALMSAGSMH	Rye	• Inhibition of linoleic peroxidation<br>• DPPH radical scavenging activity	(Coda et al., 2012)
516	LAGNPHQQQQN	Walnut meal (*Juglans regia* L.)	• Hydroxyl radical scavenging activity<br>• Increase viability of SH-SY5Y cells that are treated with H_2O_2<br>• Reduce intracellular ROS	(Sheng et al., 2019)
517	LAQGTFLQPHQ	Wheat (*Triticum aestivum*)	Antioxidant	(Babini et al., 2017)
518	LPENAKVDQVK	Brown rice	• ABTS radical scavenging activity<br>• Cu^{2+} chelating activity	(Selamassakul et al., 2018)

519	MEGPSHGVHPL	Tomato seed (*Solanum lycopersicum*)	• DPPH radical scavenging activity<br>• Phosphomolybdate reducing activity	(Meshginfar et al., 2018)
520	MPVDVIANAYR	Brown rice	• ABTS radical scavenging activity<br>• Cu^{2+} chelating activity	(Selamassakul et al., 2018)
521	PAEMVAAALDR	Rye	• Inhibition of linoleic peroxidation<br>• DPPH radical scavenging activity<br>• High hydrophobicity, access hydrophobic radical species	(Coda et al., 2012)
522	PEFEEEQPHRP	Tomato seed (*Solanum lycopersicum*)	• DPPH radical scavenging activity<br>• Phosphomolybdate reducing activity	(Meshginfar et al., 2018)
523	QVVRSDQGSVR	Brown rice	• ABTS radical scavenging activity<br>• Cu^{2+} chelating activity	(Selamassakul et al., 2018)
524	SILSDEEDERQ	Lotus seed (*Nelumbo nucifera* Gaertn.)	• DPPH radical scavenging activity<br>• ABTS radical scavenging activity<br>• H_2O_2 scavenging activity<br>• Reducing power activity	(Yu et al., 2021)
525	SRSDQDNPFIF	Lentil seeds (*Lens culinaris* var. Castellana)	Peptides identified from lentil hydrolysates produced by HP-assisted proteolysis using Savinase at 300 MPa. Highest ORAC values were observed.	(Garcia-Mora et al., 2015)
526	TETWNPNHPEL	Chickpea (*Cicer arietinum*)	Antioxidant	(Torres-Fuentes et al., 2015)
527	TSLDLPVLRWL	Lentil seeds (*Lens culinaris* var. Castellana)	Identified from S2 hydrolysate (which has the highest ORAC values).	(Garcia-Mora et al., 2014)
528	VEGNLQVLRPR	Walnut meal (*Juglans regia* L.)	• Hydroxyl radical scavenging activity<br>• Increase viability of SH-SY5Y cells that are treated with H_2O_2<br>• Reduce intracellular ROS	(Sheng et al., 2019)
529	VTSLDLPVLRW	Garden pea (*Pisum sativum*)	Antioxidant	(Babini et al., 2017)
530	VVYFDQTQAQA	Brown rice	• ABTS radical scavenging activity<br>• Cu^{2+} chelating activity	(Selamassakul et al., 2018)
531	WSVWEQELEDR	Walnut meal (*Juglans regia* L.)	• Hydroxyl radical scavenging activity<br>• Increase viability of SH-SY5Y cells that are treated with H_2O_2<br>• Reduce intracellular ROS	(Sheng et al., 2019)
532	YNILSGFDTEL	Brown rice	• ABTS radical scavenging activity<br>• Cu^{2+} chelating activity	(Selamassakul et al., 2018)
533	YQETSSSSSQE	Brown rice	• ABTS radical scavenging activity<br>• Cu^{2+} chelating activity	(Selamassakul et al., 2018)
534	AAAAAGGGEGEG	Brown rice	• ABTS radical scavenging activity<br>• Cu^{2+} chelating activity	(Selamassakul et al., 2018)
535	AGEQGFEYVTFR	Sesame seed (*Sesamum indicum* L.)	• DPPH radical scavenging activity<br>• ABTS radical scavenging activity	(Lu et al., 2019)
536	AGNDGFEYVTLK	Walnut meal (*Juglans regia* L.)	• Hydroxyl radical scavenging activity<br>• Increase viability of SH-SY5Y cells that are treated with H_2O_2<br>• Reduce intracellular ROS	(Sheng et al., 2019)
537	AKIDWKETPQAH	Tomato seed (*Solanum lycopersicum*)	• DPPH radical scavenging activity<br>• Phosphomolybdate reducing activity	(Meshginfar et al., 2018)

538	ALEPTHQIEAEA	Lotus seed (*Nelumbo nucifera* Gaertn.)	• DPPH radical scavenging activity<br>• ABTS radical scavenging activity<br>• H_2O_2 scavenging activity<br>• Reducing power activity	(Yu et al., 2021)
539	AQQQEQAQQQEQ	Brown rice	• $ABTS^{\cdot+}$ scavenging activity<br>• Cu^{2+} chelating activity	(Selamassakul et al., 2018)
540	EQEEEESTGRMK	Walnut meal (*Juglans regia* L.)	• Hydroxyl radical scavenging activity<br>• Increase viability of SH-SY5Y cells that are treated with H_2O_2<br>• Reduce intracellular ROS	(Sheng et al., 2019)
541	FAFGINAENNQR	Soybean	• ABTS radical scavenging activity<br>• Peroxyl radical scavenging capacity<br>• Increased No_2^- production<br>• Decrease in TBARS levels (lipid peroxidation)<br>• Increase in H-Caco-2 cells of MnSOD and CAT activity	(Amigo-Benavent et al., 2014)
542	FEITPEKNPQLR	Soybean	• ABTS radical scavenging activity<br>• Peroxyl radical scavenging capacity<br>• Increased No_2^- production<br>• Decrease in TBARS levels (lipid peroxidation)<br>• Increase in H-Caco-2 cells of MnSOD and CAT activity	(Amigo-Benavent et al., 2014)
543	GGVPRSGEQEQQ	Sesame seed (*Sesamum indicum* L.)	• DPPH radical scavenging activity<br>• ABTS radical scavenging activity	(Lu et al., 2019)
544	GLHLPSFSPSPQ	Chickpea seeds	• Reducing power activity<br>• DPPH radical scavenging activity<br>• Inhibit peroxyl radical-induced DCFH oxidation to DCF in Caco-2 cells	(Torres-Fuentes et al., 2015)
545	GNQEKVLELVQR	Spelt	• Inhibition of linoleic peroxidation<br>• DPPH radical scavenging activity	(Coda et al., 2012)
546	GPIYSNEFGKFF	Garden pea (*Pisum sativum*)	Antioxidant	(Babini et al., 2017)
547	GVQEGAGHYALL	Palm Kernel cake	• DPPH radical scavenging activity<br>• Metal chelating activity<br>• Chelating activity is low	(Zarei et al., 2014)
548	GVSNAAVVAGGH	Kamut	• Inhibition of linoleic peroxidation<br>• DPPH radical scavenging activity<br>• C-terminal His residue acts as radical scavenger	(Coda et al., 2012)
549	HHLGGAKQAGDV	Velvet bean (*Mucuna pruriens*)	Antioxidant	(Herrera-ChaléFrancisco et al., 2016)
550	HNAVDSQIAGKA	Brown rice	• ABTS radical scavenging activity<br>• Cu^{2+} chelating activity	(Selamassakul et al., 2018)
551	KEFLLAGNNNRA	Brown rice	• ABTS radical scavenging activity<br>• Cu^{2+} chelating activity	(Selamassakul et al., 2018)
552	LTETWNPNHPEL	Chickpea seeds	• Reducing power activity<br>• DPPH radical scavenging activity<br>• Inhibit peroxyl radical-induced DCFH oxidation to DCF in Caco-2 cells	(Torres-Fuentes et al., 2015)
553	MDATALHYENQK	Wheat germ	ABTS scavenging activity	(Karami et al., 2019)
554	NEGALLLPHFNS	Soybean	• ABTS radical scavenging activity	(Amigo-Benavent et al., 2014)

			• Peroxyl radical scavenging capacity<br>• Increased No_2^- production<br>• Decrease in TBARS levels (lipid peroxidation)<br>• Increase in H-Caco-2 cells of MnSOD and CAT activity	
555	NKRSPQLQNLRD	Soybean	• ABTS radical scavenging activity<br>• Peroxyl radical scavenging capacity<br>• Increased No_2^- production<br>• Decrease in TBARS levels (lipid peroxidation)<br>• Increase in H-Caco-2 cells of MnSOD and CAT activity	(Amigo-Benavent et al., 2014)
556	NQRSPQLQNLRD	Soybean	• ABTS radical scavenging activity<br>• Peroxyl radical scavenging capacity<br>• Increased No_2^- production<br>• Decrease in TBARS levels (lipid peroxidation)<br>• Increase in H-Caco-2 cells of MnSOD and CAT activity	(Amigo-Benavent et al., 2014)
557	SILSDEEDERQQ	Lotus seed (*Nelumbo nucifera* Gaertn.)	• DPPH radical scavenging activity<br>• ABTS radical scavenging activity<br>• H_2O_2 scavenging activity<br>• Reducing power activity	(Yu et al., 2021)
558	STTTKKHHPQYL	Tomato seed (*Solanum lycopersicum*)	• DPPH radical scavenging activity<br>• Phosphomolybdate reducing activity	(Meshginfar et al., 2018)
559	TVTSLDLPVLRW	Garden pea (*Pisum sativum*)	Antioxidant	(Babini et al., 2017)
560	VEINEGGLLLPH	Lentil seeds (*Lens culinaris* var. Castellana)	Identified from S2 hydrolysate (which has the highest ORAC values).	(Garcia-Mora et al., 2014)
561	YFPTQALNFAFK	Emmer (*Triticum dicoccum*)	Antioxidant	(Babini et al., 2017)
562	YFPVGGDRPESF	Rice (*Oryza sativa*)	Antioxidant	(Adebiyi et al., 2008)
563	YQETSSSSSQEQ	Brown rice	• ABTS radical scavenging activity<br>• Cu^{2+} chelating activity	(Selamassakul et al., 2018)
564	AAVKKMYDIQAKK	Brown rice	• ABTS radical scavenging activity<br>• Cu^{2+} chelating activity	(Selamassakul et al., 2018)
565	ACSNHSPLGWRGH	Kidney bean (*Phaseolus vulgaris*)	Antioxidant	(Ngoh and Gan, 2016)
566	AELQVVDHLGQTV	Walnut meal (*Juglans regia* L.)	• Hydroxyl radical scavenging activity<br>• Increase viability of SH-SY5Y cells that are treated with H_2O_2<br>• Reduce intracellular ROS	(Sheng et al., 2019)
567	AIPVNRPGQLQSF	Lentil seeds (*Lens culinaris* var. Castellana)	Peptides identified from lentil hydrolysates produced by HP-assisted proteolysis using Savinase at 300 MPa. Highest ORAC values were observed.	(Garcia-Mora et al., 2015)
568	AVYVYDVNNNANQ	Brown rice	• ABTS radical scavenging activity<br>• Cu^{2+} chelating activity	(Selamassakul et al., 2018)
569	DLAIPVNRPGQLQ	Lentil seeds (*Lens culinaris* var. Castellana)	Peptides identified from lentil hydrolysates produced by HP-assisted proteolysis using Savinase at 300	(Garcia-Mora et al., 2015)

			MPa. Highest ORAC values were observed.	
570	FDTADLPSGKGYL	Brown rice	• ABTS radical scavenging activity<br>• Cu^{2+} chelating activity	(Selamassakul et al., 2018)
571	FEITPEKNPQLRD	Soybean	• ABTS radical scavenging activity<br>• Peroxyl radical scavenging capacity<br>• Increased No_2^- production<br>• Decrease in TBARS levels (lipid peroxidation)<br>• Increase in H-Caco-2 cells of MnSOD and CAT activity	(Amigo-Benavent et al., 2014)
572	FVDAQPKKKEEGN	Soybean	• ABTS radical scavenging activity<br>• Peroxyl radical scavenging capacity<br>• Increased No_2^- production<br>• Decrease in TBARS levels (lipid peroxidation)<br>• Increase in H-Caco-2 cells of MnSOD and CAT activity	(Amigo-Benavent et al., 2014)
573	FVDAQPQQKEEGN	Soybean	• ABTS radical scavenging activity<br>• Peroxyl radical scavenging capacity<br>• Increased No_2^- production<br>• Decrease in TBARS levels (lipid peroxidation)<br>• Increase in H-Caco-2 cells of MnSOD and CAT activity	(Amigo-Benavent et al., 2014)
574	GENDQRGGIVNVE	Lotus seed (*Nelumbo nucifera* Gaertn.)	• DPPH radical scavenging activity<br>• ABTS radical scavenging activity<br>• H_2O_2 scavenging activity<br>• Reducing power activity	(Yu et al., 2021)
575	GGRLDSGKQPPRQ	Brown rice	• ABTS radical scavenging activity<br>• Cu^{2+} chelating activity	(Selamassakul et al., 2018)
576	KKSKADLTEVTHK	Brown rice	• ABTS radical scavenging activity<br>• Cu^{2+} chelating activity	(Selamassakul et al., 2018)
577	LAIPVNKPGRFES	Soybean	• ABTS radical scavenging activity<br>• Peroxyl radical scavenging capacity<br>• Increased No_2^- production<br>• Decrease in TBARS levels (lipid peroxidation)<br>• Increase in H-Caco-2 cells of MnSOD and CAT activity	(Amigo-Benavent et al., 2014)
578	LQGENDQRGPIIQ	Lotus seed (*Nelumbo nucifera* Gaertn.)	• DPPH radical scavenging activity<br>• ABTS radical scavenging activity<br>• H_2O_2 scavenging activity<br>• Reducing power activity	(Yu et al., 2021)
579	LRENNKLMLLELK	Kidney bean (*Phaseolus vulgaris*)	Antioxidant	(Mojica, Chen and Mejía, 2015)
580	QEINKENVIVKVS	Lentil seeds (*Lens culinaris* var. Castellana)	Identified from S2 hydrolysate (which has the highest ORAC values).	(Garcia-Mora et al., 2014)
581	QNIFSGFSTELLS	Brown rice	• ABTS radical scavenging activity<br>• Cu^{2+} chelating activity	(Selamassakul et al., 2018)
582	QQFQQSGQAQLTE	Brown rice	• ABTS radical scavenging activity<br>• Cu^{2+} chelating activity	(Selamassakul et al., 2018)
583	SEALGVSSQVARQ	Brown rice	• ABTS radical scavenging activity<br>• Cu^{2+} chelating activity	(Selamassakul et al., 2018)

584	SILSDEEDERQQE	Lotus seed (*Nelumbo nucifera* Gaertn.)	• DPPH radical scavenging activity<br>• ABTS radical scavenging activity<br>• H_2O_2 scavenging activity<br>• Reducing power activity	(Yu et al., 2021)
585	SLSLPNFHPMPRL	Tomato seed (*Solanum lycopersicum*)	• DPPH radical scavenging activity<br>• Phosphomolybdate reducing activity	(Meshginfar et al., 2018)
586	SPDIYNPEAGRIK	Pea	• Superoxide anion and hydroxyl radicals scavenging activity<br>• ABTS radical scavenging activity<br>• DPPH radical scavenging activity<br>• Lipid peroxidation inhibition	(Babini et al., 2017)
587	SPFLQSAAFQLRN	Brown rice	• ABTS radical scavenging activity<br>• Cu^{2+} chelating activity	(Selamassakul et al., 2018)
588	SVEIKEGSLLLPH	Lentil seeds (*Lens culinaris* var. Castellana)	Identified from S2 hydrolysate (which has the highest ORAC values).	(Garcia-Mora et al., 2014)
589	SVEINEGGLLLPH	Lentil seeds (*Lens culinaris* var. Castellana)	Identified from S2 hydrolysate (which has the highest ORAC values).	(Garcia-Mora et al., 2014)
590	TAGTGGGQFQPMR	Brown rice	• ABTS radical scavenging activity<br>• Cu^{2+} chelating activity	(Selamassakul et al., 2018)
591	TVTSLDLPVLRWL	Lentil seeds (*Lens culinaris* var. Castellana)	Identified from S2 hydrolysate (which has the highest ORAC values).	(Garcia-Mora et al., 2014)
592	DMNEGALLLPHFNS	Soybean	• ABTS radical scavenging activity<br>• Peroxyl radical scavenging capacity<br>• Increased No_2^- production<br>• Decrease in TBARS levels (lipid peroxidation)<br>• Increase in H-Caco-2 cells of MnSOD and CAT activity	(Amigo-Benavent et al., 2014)
593	DQSYFVAGPEHRQQ	Tomato seed (*Solanum lycopersicum*)	• DPPH radical scavenging activity<br>• Phosphomolybdate reducing activity	(Meshginfar et al., 2018)
594	DVYKIGGIGTVPVG	Emmer	DPPH radical scavenging activity	(Babini et al., 2017)
595	MAPAAVAAAEAGS K	Whole wheat	• Inhibition of linoleic peroxidation<br>• DPPH radical scavenging activity<br>• High hydrophobicity, access hydrophobic radical species	(Coda et al., 2012)
596	NEGALLLPHFNSKA	Soybean	• ABTS radical scavenging activity<br>• Peroxyl radical scavenging capacity<br>• Increased No_2^- production<br>• Decrease in TBARS levels (lipid peroxidation)<br>• Increase in H-Caco-2 cells of MnSOD and CAT activity	(Amigo-Benavent et al., 2014)
597	NIGHPTRSDVYNPR	Tomato seed (*Solanum lycopersicum*)	• DPPH radical scavenging activity<br>• Phosphomolybdate reducing activity	(Meshginfar et al., 2018)
598	NSSYFVEWIPNNVK	Emmer (*Triticum dicoccum*)	Antioxidant	(Babini et al., 2017)
599	QTIYENENGHIRLL	Lentil seeds (*Lens culinaris* var. Castellana)	Peptides identified from lentil hydrolysates produced by HP-assisted proteolysis using Savinase at 300 MPa. Highest ORAC values were observed.	(Garcia-Mora et al., 2015)

600	RRQEINKENVIVKV	Lentil seeds (*Lens culinaris* var. Castellana)	Identified from S2 hydrolysate (which has the highest ORAC values).	(Garcia-Mora et al., 2014)
601	SGGSYADELVSTAK	Wheat germ	ABTS radical scavenging activity	(Karami et al., 2019)
602	SILSEDEEQQQEKN	Lotus seed (*Nelumbo nucifera* Gaertn.)	• DPPH radical scavenging activity<br>• ABTS radical scavenging activity<br>• H_2O_2 scavenging activity<br>• Reducing power activity	(Yu et al., 2021)
603	SIVDMNEGALLLPH	Soybean	• ABTS radical scavenging activity<br>• Peroxyl radical scavenging capacity<br>• Increased No_2^- production<br>• Decrease in TBARS levels (lipid peroxidation)<br>• Increase in H-Caco-2 cells of MnSOD and CAT activity	(Amigo-Benavent et al., 2014)
604	SSPDIYNPEAGRIK	Pea	• Superoxide anion and hydroxyl radicals scavenging activity.<br>• ABTS radical scavenging activity.<br>• DPPH radical scavenging activity<br>• Lipid peroxidation inhibition	(Babini et al., 2017)
605	SVEINEGGLLLPHY	Lentil seeds (*Lens culinaris* var. Castellana)	Identified from S2 hydrolysate (which has the highest ORAC values).	(Garcia-Mora et al., 2014)
606	VVSNFGKTVFDGVL	Brown rice	• ABTS radical scavenging activity<br>• Cu^{2+} chelating activity	(Selamassakul et al., 2018)
607	DEEEGQEEETTKQVQ	Lentil seeds (*Lens culinaris* var. Castellana)	Peptides identified from lentil hydrolysates produced by HP-assisted proteolysis using Savinase at 300 MPa. Highest ORAC values were observed.	(Garcia-Mora et al., 2015)
608	DLAIPVNRPGQLQSF	Lentil seeds (*Lens culinaris* var. Castellana)	Peptides identified from lentil hydrolysates produced by HP-assisted proteolysis using Savinase at 300 MPa. Highest ORAC values were observed.	(Garcia-Mora et al., 2015)
609	FEITPEKNPQLRDLD	Soybean	• ABTS radical scavenging activity<br>• Peroxyl radical scavenging capacity<br>• Increased No_2^- production<br>• Decrease in TBARS levels (lipid peroxidation)<br>• Increase in H-Caco-2 cells of MnSOD and CAT activity	(Amigo-Benavent et al., 2014)
610	LSIVDMNEGALLLPH	Soybean	• ABTS radical scavenging activity<br>• Peroxyl radical scavenging capacity<br>• Increased No_2^- production<br>• Decrease in TBARS levels (lipid peroxidation)<br>• Increase in H-Caco-2 cells of MnSOD and CAT activity	(Amigo-Benavent et al., 2014)
611	NAENNQRNFLAGSQD	Soybean	• ABTS radical scavenging activity<br>• Peroxyl radical scavenging capacity<br>• Increased No_2^- production<br>• Decrease in TBARS levels (lipid peroxidation)<br>• Increase in H-Caco-2 cells of MnSOD and CAT activity	(Amigo-Benavent et al., 2014)
612	NLQSGDALRVPAGTT	Soybean	• ABTS radical scavenging activity	(Amigo-Benavent et al., 2014)

			• Peroxyl radical scavenging capacity<br>• Increased No_2^- production<br>• Decrease in TBARS levels (lipid peroxidation)<br>• Increase in H-Caco-2 cells of MnSOD and CAT activity	
613	QPSPQDYLNAHNAAR	Chañar seeds (*Geoffroea decorticans*)	• DPPH radical scavenging activity<br>• ABTS radical scavenging activity	(Cotabarren et al., 2021)
614	REQEQEQEEGDVHYQ	Tomato seed (*Solanum lycopersicum*)	• DPPH radical scavenging activity<br>• Phosphomolybdate reducing activity	(Meshginfar et al., 2018)
615	RRQEINKENVIVKVS	Lentil seeds (*Lens culinaris* var. Castellana)	Identified from S2 hydrolysate (which has the highest ORAC values).	(Garcia-Mora et al., 2014)
616	SILSDEEDERQQEKN	Lotus seed (*Nelumbo nucifera* Gaertn.)	• DPPH radical scavenging activity<br>• ABTS radical scavenging activity<br>• H_2O_2 scavenging activity<br>• Reducing power activity	(Yu et al., 2021)
617	SVEIKEGSLLLPHYN	Lentil seeds (*Lens culinaris* var. Castellana)	Identified from S2 hydrolysate (which has the highest ORAC values).	(Garcia-Mora et al., 2014)
618	VNSVEIKEGSLLLPH	Lentil seeds (*Lens culinaris* var. Castellana)	Identified from S2 hydrolysate (which has the highest ORAC values).	(Garcia-Mora et al., 2014)
619	YVVNPDNNENLRLIT	Soybean	• ABTS radical scavenging activity<br>• Peroxyl radical scavenging capacity<br>• Increased No_2^- production<br>• Decrease in TBARS levels (lipid peroxidation)<br>• Increase in H-Caco-2 cells of MnSOD and CAT activity	(Amigo-Benavent et al., 2014)
620	AAGAAAAARSAGQCGR	Spelt	• Inhibition of linoleic peroxidation<br>• DPPH radical scavenging activity<br>• Show highest activity	(Coda et al., 2012)
621	ASEEQIRAISEHASRS	Tomato seed (*Solanum lycopersicum*)	• DPPH radical scavenging activity<br>• Phosphomolybdate reducing activity	(Meshginfar et al., 2018)
622	DLAIPVNRPGQLQSFLL	Lentil seeds (*Lens culinaris* var. Castellana)	Peptides identified from lentil hydrolysates produced by HP-assisted proteolysis using Savinase at 300 MPa. Highest ORAC values were observed.	(Garcia-Mora et al., 2015)
623	DRRQEINKENVIVKVS	Lentil seeds (*Lens culinaris* var. Castellana)	Identified from S2 hydrolysate (which has the highest ORAC values).	(Garcia-Mora et al., 2014)
624	HKEMQAIFDVYIMFIN	Kamut	• Inhibition of linoleic peroxidation<br>• DPPH radical scavenging activity<br>• N-terminal His residue acts as metal ion chelator	(Coda et al., 2012)
625	LNSGDALRVPSGTTYY	Soybean (*Glycine max*)	Antioxidant	(Chen, Muramoto and Yamauchi, 1995)
626	LQSGDALRVPSGTTYY	Soybean (*Glycine max*)	Antioxidant	(Chen, Muramoto and Yamauchi, 1995)
627	LSSLEMGSLGALFVCM	Kidney bean (*Phaseolus vulgaris*)	Antioxidant	(Ngoh and Gan, 2016)
628	RLSLPAGAPVTVAVSP	Rye	• Inhibition of linoleic peroxidation<br>• DPPH radical scavenging activity	(Coda et al., 2012)

629	SVEIKEGSLLLPHYNS	Lentil seeds (*Lens culinaris* var. Castellana)	Identified from S2 hydrolysate (which has the highest ORAC values).	(Garcia-Mora et al., 2014)
630	SWNLQNGERANVVIAF	Lentil seeds (*Lens culinaris* var. Castellana)	Identified from S2 hydrolysate (which has the highest ORAC values).	(Garcia-Mora et al., 2014)
631	VEIKEGSLLLPHYNSR	Lentil seeds (*Lens culinaris* var. Castellana)	Identified from S2 hydrolysate (which has the highest ORAC values).	(Garcia-Mora et al., 2014)
632	AGTGAGAGGGAGTKTSS	Brown rice	• ABTS radical scavenging activity<br>• Cu^{2+} chelating activity	(Selamassakul et al., 2018)
633	DPAQPNYPWTAVLVFRH	Cumin seeds (*Cuminum cyminum*)	Antioxidant	(Siow and Gan, 2016)
634	DSEGHRTAPCYVMKILF	Bitter bean (*Parkia speciosa*)	Antioxidant	(Siow and Gan, 2013)
635	FEGTVFENGIDAAYRST	Lentil seeds (*Lens culinaris* var. Castellana)	Identified from S2 hydrolysate (which has the highest ORAC values).	(Garcia-Mora et al., 2014)
636	GPSSSPDIYNPEAGRIK	Garden pea (*Pisum sativum*)	Antioxidant	(Babini et al., 2017)
637	KLQGENDQRGPIIHVKE	Lotus seed (*Nelumbo nucifera* Gaertn.)	• DPPH radical scavenging activity<br>• ABTS radical scavenging activity<br>• H_2O_2 scavenging activity<br>• Reducing power activity	(Yu et al., 2021)
638	NLERGDTIKLPAGTIAY	Lentil seeds (*Lens culinaris* var. Castellana)	Identified from S2 hydrolysate (which has the highest ORAC values).	(Garcia-Mora et al., 2014)
639	RFQTLYENENGHIRLLQ	Lentil seeds (*Lens culinaris* var. Castellana)	Identified from S2 hydrolysate (which has the highest ORAC values).	(Garcia-Mora et al., 2014)
640	SVEINEGGLLLPHYNSR	Lentil seeds (*Lens culinaris* var. Castellana)	Identified from S2 hydrolysate (which has the highest ORAC values).	(Garcia-Mora et al., 2014)
641	TIDPNGLHLPSFSPSPQ	Chickpea seeds	• Reducing power activity<br>• DPPH radical scavenging activity<br>• Inhibit peroxyl radical-induced DCFH oxidation to DCF in Caco-2 cells	(Torres-Fuentes et al., 2015)
642	TVVPPKGGSFYPGETTP	Wheat (*Triticum aestivum*)	Antioxidant	(Babini et al., 2017)
643	VAPDMEHPHGTPGHRHH	Tomato seed (*Solanum lycopersicum*)	• DPPH radical scavenging activity<br>• Phosphomolybdate reducing activity	(Meshginfar et al., 2018)
644	EGSLLLPHYNSRAIVIVT	Lentil seeds (*Lens culinaris* var. Castellana)	Identified from S2 hydrolysate (which has the highest ORAC values).	(Garcia-Mora et al., 2014)
645	IGPSSSPDIYNPEAGRIK	Garden pea (*Pisum sativum*)	Antioxidant	(Babini et al., 2017)
646	NFLAGSKDNVISQIPSQV	Soybean	• ABTS radical scavenging activity<br>• Peroxyl radical scavenging capacity<br>• Increased No_2^- production<br>• Decrease in TBARS levels (lipid peroxidation)<br>• Increase in H-Caco-2 cells of MnSOD and CAT activity	(Amigo-Benavent et al., 2014)
647	NLERGDTIKLPAGTIAYL	Lentil seeds (*Lens culinaris* var. Castellana)	Identified from S2 hydrolysate (which has the highest ORAC values).	(Garcia-Mora et al., 2014)

648	NRFQTLYENENGHIR LLQ	Lentil seeds (*Lens culinaris* var. Castellana)	Identified from S2 hydrolysate (which has the highest ORAC values).	(Garcia-Mora et al., 2014)
649	QAGTGAGAGGGAG TKTSS	Brown rice	• ABTS radical scavenging activity<br>• Cu^{2+} chelating activity	(Selamassakul et al., 2018)
650	VGIKEQQQKQKQEE EPLE	Soybean	• ABTS radical scavenging activity<br>• Peroxyl radical scavenging capacity<br>• Increased No_2^- production<br>• Decrease in TBARS levels (lipid peroxidation)<br>• Increase in H-Caco-2 cells of MnSOD and CAT activity	(Amigo-Benavent et al., 2014)
651	VGLKEQQQEQQQEE QPLE	Soybean	• ABTS radical scavenging activity<br>• Peroxyl radical scavenging capacity<br>• Increased No_2^- production<br>• Decrease in TBARS levels (lipid peroxidation)<br>• Increase in H-Caco-2 cells of MnSOD and CAT activity	(Amigo-Benavent et al., 2014)
652	LAFPGSAQDVERLL KEQRE	Soybean	• ABTS radical scavenging activity<br>• Peroxyl radical scavenging capacity<br>• Increased No_2^- production<br>• Decrease in TBARS levels (lipid peroxidation)<br>• Increase in H-Caco-2 cells of MnSOD and CAT activity	(Amigo-Benavent et al., 2014)
653	NIGPSSSPDIYNPEAG RIK	Pea	• Superoxide anion and hydroxyl radicals scavenging activity.<br>• ABTS radical scavenging activity<br>• DPPH radical scavenging activity<br>• Lipid peroxidation inhibition	(Babini et al., 2017)
654	VNSVEIKEGSLLLPH YNSR	Lentil seeds (*Lens culinaris* var. Castellana)	Identified from S2 hydrolysate (which has the highest ORAC values).	(Garcia-Mora et al., 2014)
655	YEWEPTVPNFDVAK DVTDM	Kamut	• Inhibition of linoleic peroxidation<br>• DPPH radical scavenging activity	(Coda et al., 2012)
656	YGENIAWSSGDLSG TAAVK	Chañar seeds (*Geoffroea decorticans*)	• DPPH radical scavenging activity<br>• ABTS radical scavenging activity	(Cotabarren et al., 2021)
657	GREQEREQEQEQEE GDVHYQ	Tomato seed (*Solanum lycopersicum*)	• DPPH radical scavenging activity<br>• Phosphomolybdate reducing activity	(Meshginfar et al., 2018)
658	LAFPGSAQAVEKLL KNQRES	Soybean	• ABTS radical scavenging activity<br>• Peroxyl radical scavenging capacity<br>• Increased No_2^- production<br>• Decrease in TBARS levels (lipid peroxidation)<br>• Increase in H-Caco-2 cells of MnSOD and CAT activity	(Amigo-Benavent et al., 2014)
659	TVVPPKGGSFYPGET TPLQQ	Wheat (*Triticum aestivum*)	Antioxidant	(Babini et al., 2017)
660	AIAGAGVLSGYDQL QILFFGK	Spelt	• Inhibition of linoleic peroxidation<br>• DPPH radical scavenging activity	(Coda et al., 2012)
661	FNTEYEEIEKVLLEE QEQKSQ	Lentil seeds (*Lens culinaris* var. Castellana)	Peptides identified from lentil hydrolysates produced by HP-assisted proteolysis using Savinase at 300	(Garcia-Mora et al., 2015)

			MPa. Highest ORAC values were observed.	
662	PPGPGPGPPPPPGAA GRGGGG	Kamut	• Inhibition of linoleic peroxidation<br>• DPPH radical scavenging activity	(Coda et al., 2012)
663	RCMAFLLSDGAAAA QQLLPQYW	Cumin seeds (*Cuminum cyminum*)	Antioxidant	(Siow and Gan, 2016)
664	FFRSKLLSDGAAAA KGALLPQYW	Cumin seeds (*Cuminum cyminum*)	Antioxidant	(Siow and Gan, 2016)
665	FLGQQQPFPPQQPYP QPQPFPSQQP	Wheat (*Triticum aestivum*)	Antioxidant	(Babini et al., 2017)
666	LGQQQPFPPQQPYPQ PQPFPSQQPY	Wheat (*Triticum aestivum*)	Antioxidant	(Babini et al., 2017)
667	NAENNQRNFLAGSQ DNVISQIPSQV	Soybean	• ABTS radical scavenging activity<br>• Peroxyl radical scavenging capacity<br>• Increased No_2^- production<br>• Decrease in TBARS levels (lipid peroxidation)<br>• Increase in H-Caco-2 cells of MnSOD and CAT activity	(Amigo-Benavent et al., 2014)
668	SEVGVPNLPWDDTV AAYAQNYANQR	Chañar seeds (*Geoffroea decorticans*)	• DPPH radical scavenging activity<br>• ABTS radical scavenging activity	(Cotabarren et al., 2021)
669	DLADIPQQQRLMAG LALVVATVIFLK	Rye	• Inhibition of linoleic peroxidation<br>• DPPH radical scavenging activity<br>• Show highest activity among peptides in rye	(Coda et al., 2012)
670	FLGQQQPFPPQQPYP QPQPFPSQQPY	Wheat (*Triticum aestivum*)	Antioxidant	(Babini et al., 2017)
671	VFVDEGLEVLGWRP VPFNVSVVGRNAK	Rye	• Inhibition of linoleic peroxidation<br>• DPPH radical scavenging activity	(Coda et al., 2012)
672	KNGSIFNSPSATAATI IHGHNYSGLAYLDF VTSK	Rye	• Inhibition of linoleic peroxidation<br>• DPPH radical scavenging activity<br>• Show highest activity among peptides in rye	(Coda et al., 2012)
673	NANGELCPNNMCCS QWGYCGLGSEFCGN GCQSGACCPEK	Rye	• Inhibition of linoleic peroxidation<br>• DPPH radical scavenging activity	(Coda et al., 2012)
674	SKWQHQQDSCRKQ KQGVNLTPCEKHIM EKIQGRGDDDDDDD DD	Soybean (*Glycine max*)	Antioxidant	(Lule et al., 2015; Singh, Vij and Hati, 2014)
675	GTIFFSQEGDGPTSV TGSVSGLKPGLHGF HVHALGDTTNGCMS TGPHFNPTGK	Rye	• Inhibition of linoleic peroxidation<br>• DPPH radical scavenging activity<br>• Show highest activity among peptides in rye	(Coda et al., 2012)
676	DTAAGYVAPPDPAV STGDYGLAGAEAPH PHESAVMSGAAAAA VAPGGEAYTR	Kamut	• Inhibition of linoleic peroxidation<br>• DPPH radical scavenging activity	(Coda et al., 2012)
677	TGGGSTSSSSSSSSL GGGASRGSVVEAAP PATQGAAAANAPAV PVVVVDTQEAGIR	Kamut	• Inhibition of linoleic peroxidation<br>• DPPH radical scavenging activity	(Coda et al., 2012)

Access date: 5-7 October 2021

References

Adebiyi, A.P., Adebiyi, A.O., Ogawa, T. and Muramoto, K., 2008. Purification and characterisation of antioxidative peptides from unfractionated rice bran protein hydrolysates. *International Journal of Food Science & Technology*, [e-journal]

43(1), pp.35–43. https://doi.org/10.1111/J.1365-2621.2006.01379.X.
Agrawal, H., Joshi, R. and Gupta, M., 2017. Isolation and characterisation of enzymatic hydrolysed peptides with antioxidant activities from green tender sorghum. *LWT*, [e-journal] 84, pp.608–616.
Amadou, I. et al., 2013. Purification and characterization of foxtail millet-derived peptides with antioxidant and antimicrobial activities. *Food Research International*, [e-journal] 51(1), pp.422–428.
Amigo-Benavent, M. et al., 2014. Use of phytochemomics to evaluate the bioavailability and bioactivity of antioxidant peptides of soybean β-conglycinin. *ELECTROPHORESIS*, [e-journal] 35(11), pp.1582–1589. https://doi.org/10.1002/ELPS.201300527.
Babini, E. et al., 2017. LC-ESI-QTOF-MS identification of novel antioxidant peptides obtained by enzymatic and microbial hydrolysis of vegetable proteins. *Food Chemistry*, [e-journal] 228, pp.186–196.
Bautista-Expósito, S. et al., 2018. Combination of pH-controlled fermentation in mild acidic conditions and enzymatic hydrolysis by Savinase to improve metabolic health-promoting properties of lentil. *Journal of Functional Foods*, [e-journal] 48, pp.9–18.
Beermann, C., Euler, M., Herzberg, J. and Stahl, B., 2009. Anti-oxidative capacity of enzymatically released peptides from soybean protein isolate. *European Food Research and Technology 2009 229:4*, [e-journal] 229(4), pp.637–644. https://doi.org/10.1007/S00217-009-1093-1.
Chai, T.T. et al., 2019. Antioxidant activity of semen cassiae protein hydrolysate: thermal and gastrointestinal stability, peptide identification, and in silico analysis. *Modern Food Science and Technology*, [e-journal] 35(9), pp.38–48.
Chai, T.T. et al., 2021. Identification of antioxidant peptides derived from tropical jackfruit seed and investigation of the stability profiles. *Food Chemistry*, [e-journal] 340, p.127876.
Chang, S.K. et al., 2015. Antioxidant peptides purified and identified from the oil palm (Elaeis guineensis Jacq.) kernel protein hydrolysate. *Journal of Functional Foods*, [e-journal] 14, pp.63–75.
Chen, H.M., Muramoto, K. and Yamauchi, F., 1995. Structural Analysis of Antioxidative Peptides from Soybean β-Conglycinin. *Journal of Agricultural and Food Chemistry*, [e-journal] 43(3), pp.574–578. https://doi.org/10.1021/jf00051a004.
Chen, H.M., Muramoto, K., Yamauchi, F. and Nokihara, K., 1996. Antioxidant Activity of Designed Peptides Based on the Antioxidative Peptide Isolated from Digests of a Soybean Protein. *Journal of Agricultural and Food Chemistry*, [e-journal] 44(9), pp.2619–2623. https://doi.org/10.1021/jf950833m.
Chen, L. et al., 2021. Characterisation of antioxidant peptides from enzymatic hydrolysate of golden melon seeds protein. *International Journal of Food Science & Technology*. https://doi.org/10.1111/IJFS.15250.
Chunkao, S. et al., 2020. Structure and function of mung bean protein- derived iron-binding antioxidant peptides. *Foods*, [e-journal] 9(10), p.1406. https://doi.org/10.3390/foods9101406.
Coda, R., Rizzello, C.G., Pinto, D. and Gobbetti, M., 2012. Selected lactic acid bacteria synthesize antioxidant peptides during sourdough fermentation of cereal flours. *Applied and Environmental Microbiology*, [e-journal] 78(4), pp.1087–1096.
Cotabarren, J. et al., 2021. Purification and identification of novel antioxidant peptides isolated from geoffroea decorticans seeds with anticoagulant activity. *Pharmaceutics*, [e-journal] 13(8), p.1153. https://doi.org/10.3390/pharmaceutics13081153.
Daliri, E.B.-M. et al., 2020. Untargeted Metabolomics of Fermented Rice Using UHPLC Q-TOF MS/MS Reveals an Abundance of Potential Antihypertensive Compounds. *Foods 2020, Vol. 9, Page 1007*, [e-journal] 9(8), p.1007. https://doi.org/10.3390/FOODS9081007.
Ding, J. et al., 2020. Optimization of pea protein hydrolysate preparation and purification of antioxidant peptides based on an in silico analytical approach. *LWT*, [e-journal] 123, p.109126.
Esfandi, R., Seidu, I., Willmore, W. and Tsopmo, A., 2021. Antioxidant, pancreatic lipase, and α-amylase inhibitory properties of oat bran hydrolyzed proteins and peptides. *Journal of Food Biochemistry*, p.e13762. https://doi.org/10.1111/JFBC.13762.
Esteve, C., Marina, M.L. and García, M.C., 2015. Novel strategy for the revalorization of olive (Olea europaea) residues based on the extraction of bioactive peptides. *Food Chemistry*, [e-journal] 167, pp.272–280.
Fang, L. et al., 2019. Protective role of hazelnut peptides on oxidative stress injury in human umbilical vein endothelial cells. *Journal of Food Biochemistry*, [e-journal] 43(3), p.e12722. https://doi.org/10.1111/JFBC.12722.
Feng, Y.X. et al., 2018. Purification, identification, and synthesis of five novel antioxidant peptides from Chinese chestnut (Castanea mollissima Blume) protein hydrolysates. *LWT*, [e-journal] 92, pp.40–46.
Gao, J. et al., 2021. Identification and molecular docking of antioxidant peptides from hemp seed protein hydrolysates. *LWT*, [e-journal] 147, p.111453.
Garcia-Mora, P. et al., 2015. High-pressure improves enzymatic proteolysis and the release of peptides with angiotensin I converting enzyme inhibitory and antioxidant activities from lentil proteins. *Food Chemistry*, [e-journal] 171, pp.224–232.
Garcia-Mora, P., Peñas, E., Frias, J. and Martínez-Villaluenga, C., 2014. Savinase, the Most Suitable Enzyme for Releasing Peptides from Lentil (Lens culinaris var. Castellana) Protein Concentrates with Multifunctional Properties. *Journal of Agricultural and Food Chemistry*, [e-journal] 62(18), pp.4166–4174. https://doi.org/10.1021/JF500849U.
García, M.C., Endermann, J., González-García, E. and Marina, M.L., 2015. HPLC-Q-TOF-MS Identification of Antioxidant and Antihypertensive Peptides Recovered from Cherry (Prunus cerasus L.) Subproducts. *Journal of Agricultural and Food Chemistry*, [e-journal] 63(5), pp.1514–1520. https://doi.org/10.1021/JF505037P.
García, M.C., Puchalska, P., Esteve, C. and Marina, M.L., 2013. Vegetable foods: A cheap source of proteins and peptides with antihypertensive, antioxidant, and other less occurrence bioactivities. *Talanta*, [e-journal] 106, pp.328–349.
Girgih, A.T. et al., 2014. Structural and functional characterization of hemp seed (Cannabis sativa L.) protein-derived antioxidant and antihypertensive peptides. *Journal of Functional Foods*, [e-journal] 6(1), pp.384–394.
Girgih, A.T., He, R. and Aluko, R.E., 2014. Kinetics and Molecular Docking Studies of the Inhibitions of Angiotensin

Converting Enzyme and Renin Activities by Hemp Seed (Cannabis sativa L.) Peptides. *Journal of Agricultural and Food Chemistry*, [e-journal] 62(18), pp.4135–4144. https://doi.org/10.1021/JF5002606.

Gu, M. et al., 2015. Identification of antioxidant peptides released from defatted walnut (Juglans Sigillata Dode) meal proteins with pancreatin. *LWT - Food Science and Technology*, [e-journal] 60(1), pp.213–220.

Guo, P., Qi, Y., Zhu, C. and Wang, Q., 2015. Purification and identification of antioxidant peptides from Chinese cherry (Prunus pseudocerasus Lindl.) seeds. *Journal of Functional Foods*, [e-journal] 19, pp.394–403.

Herrera-ChaléFrancisco, Carlos, R.-R., Betancur-AnconaDavid and Rubi, S.-C., 2016. Potential Therapeutic Applications of Mucuna pruriens Peptide Fractions Purified by High-Performance Liquid Chromatography as Angiotensin-Converting Enzyme Inhibitors, Antioxidants, Antithrombotic and Hypocholesterolemic Agents. *https://home.liebertpub.com/jmf*, [e-journal] 19(2), pp.187–195. https://doi.org/10.1089/JMF.2015.0098.

Hong, J. et al., 2014. Purification and characterization of an antioxidant peptide (GSQ) from Chinese leek (Allium tuberosum Rottler) seeds. *Journal of Functional Foods*, [e-journal] 10, pp.144–153.

Jie, Y. et al., 2019. Isolation of antioxidative peptide from the protein hydrolysate of Caragana ambigua seeds and its mechanism for retarding lipid auto-oxidation. *Journal of the Science of Food and Agriculture*, [e-journal] 99(6), pp.3078–3085. https://doi.org/10.1002/JSFA.9521.

Jiménez-Escrig, A., Alaiz, M., Vioque, J. and Rupérez, P., 2009. Health-promoting activities of ultra-filtered okara protein hydrolysates released by in vitro gastrointestinal digestion: identification of active peptide from soybean lipoxygenase. *European Food Research and Technology 2009 230:4*, [e-journal] 230(4), pp.655–663. https://doi.org/10.1007/S00217-009-1203-0.

Jin, D.X. et al., 2016. Preparation of antioxidative corn protein hydrolysates, purification and evaluation of three novel corn antioxidant peptides. *Food Chemistry*, [e-journal] 204, pp.427–436.

Karami, Z. et al., 2019. Identification and synthesis of multifunctional peptides from wheat germ hydrolysate fractions obtained by proteinase K digestion. *Journal of Food Biochemistry*, [e-journal] 43(4), p.e12800. https://doi.org/10.1111/JFBC.12800.

Kim, J.M., Liceaga, A.M. and Yoon, K.Y., 2019. Purification and identification of an antioxidant peptide from perilla seed (Perilla frutescens) meal protein hydrolysate. *Food Science and Nutrition*, [e-journal] 7(5), pp.1645–1655. https://doi.org/10.1002/fsn3.998.

Kou, X. et al., 2013. Purification and identification of antioxidant peptides from chickpea (Cicer arietinum L.) albumin hydrolysates. *LWT - Food Science and Technology*, [e-journal] 50(2), pp.591–598.

Lapsongphon, N. and Yongsawatdigul, J., 2013. Production and purification of antioxidant peptides from a mungbean meal hydrolysate by Virgibacillus sp. SK37 proteinase. *Food Chemistry*, [e-journal] 141(2), pp.992–999.

Liang, L. li et al., 2020. Purification of antioxidant peptides of Moringa oleifera seeds and their protective effects on H2O2 oxidative damaged Chang liver cells. *Journal of Functional Foods*, [e-journal] 64, p.103698.

Liu, C. et al., 2018. Cytoprotective effect and purification of novel antioxidant peptides from hazelnut (C. heterophylla Fisch) protein hydrolysates. *Journal of Functional Foods*, [e-journal] 42, pp.203–215.

Lu, X. et al., 2019. Extraction, identification and structure-activity relationship of antioxidant peptides from sesame (Sesamum indicum L.) protein hydrolysate. *Food Research International*, [e-journal] 116, pp.707–716.

Lule, V.K. et al., 2015. "Potential Health Benefits of Lunasin: A Multifaceted Soy-Derived Bioactive Peptide." *Journal of Food Science*, [e-journal] 80(3), pp.R485–R494. https://doi.org/10.1111/1750-3841.12786.

Ma, C. et al., 2019. A new dual-peptide strategy for enhancing antioxidant activity and exploring the enhancement mechanism. *Food & Function*, [e-journal] 10(11), pp.7533–7543. https://doi.org/10.1039/C9FO01858A.

Ma, Y. et al., 2010. Fractionation and evaluation of radical scavenging peptides from in vitro digests of buckwheat protein. *Food Chemistry*, [e-journal] 118(3), pp.582–588.

Matsui, T., Li, C.-H. and Osajima, Y., 1999. Preparation and Characterization of Novel Bioactive Peptides Responsible for Angiotensin I-Converting Enzyme Inhibition from Wheat Germ. *Journal of Peptide Science J. Peptide Sci*, [e-journal] 5, pp.289–297.

Meshginfar, N. et al., 2018. Production of antioxidant peptide fractions from a by-product of tomato processing: mass spectrometry identification of peptides and stability to gastrointestinal digestion. *Journal of Food Science and Technology 2018 55:9*, [e-journal] 55(9), pp.3498–3507. https://doi.org/10.1007/S13197-018-3274-Z.

Mohana Dass, S., Chai, T.T. and Wong, F.C., 2019. Antioxidant and protein protection potentials of fennel seed-derived protein hydrolysates and peptides. *Modern Food Science and Technology*, [e-journal] 35(9), pp.22–49.

Mojica, L., Chen, K. and Mejía, E.G. de, 2015. Impact of Commercial Precooking of Common Bean (Phaseolus vulgaris) on the Generation of Peptides, After Pepsin–Pancreatin Hydrolysis, Capable to Inhibit Dipeptidyl Peptidase-IV. *Journal of Food Science*, [e-journal] 80(1), pp.H188–H198. https://doi.org/10.1111/1750-3841.12726.

Mojica, L. and de Mejía, E.G., 2015. Characterization and Comparison of Protein and Peptide Profiles and their Biological Activities of Improved Common Bean Cultivars (Phaseolus vulgaris L.) from Mexico and Brazil. *Plant Foods for Human Nutrition 2015 70:2*, [e-journal] 70(2), pp.105–112. https://doi.org/10.1007/S11130-015-0477-6.

Ngoh, Y.Y. and Gan, C.Y., 2016. Enzyme-assisted extraction and identification of antioxidative and α-amylase inhibitory peptides from Pinto beans (Phaseolus vulgaris cv. Pinto). *Food Chemistry*, [e-journal] 190, pp.331–337.

Puchalska, P., Concepción García, M. and Luisa Marina, M., 2014. Identification of native angiotensin-I converting enzyme inhibitory peptides in commercial soybean based infant formulas using HPLC-Q-ToF-MS. *Food Chemistry*, [e-journal] 157, pp.62–69.

Qin, X.Y. et al., 2020. Structure and composition of a potential antioxidant obtained from the chelation of pea oligopeptide and sodium selenite. *Journal of Functional Foods*, [e-journal] 64, p.103619.

Ren, X. et al., 2018. Stability and antioxidant activities of corn protein hydrolysates under simulated gastrointestinal digestion. *Cereal Chemistry*, [e-journal] 95(6), pp.760–769. https://doi.org/10.1002/CCHE.10092.

Samaei, S.P. et al., 2021. Antioxidant and Angiotensin I-Converting Enzyme (ACE) Inhibitory Peptides Obtained from

Alcalase Protein Hydrolysate Fractions of Hemp (Cannabis sativa L.) Bran. *Journal of Agricultural and Food Chemistry*, [e-journal] 69(32), pp.9220–9228. https://doi.org/10.1021/ACS.JAFC.1C01487.

Selamassakul, O. et al., 2020. Bioactive peptides from brown rice protein hydrolyzed by bromelain: Relationship between biofunctional activities and flavor characteristics. *Journal of Food Science*, [e-journal] 85(3), pp.707–717. https://doi.org/10.1111/1750-3841.15052.

Selamassakul, O. et al., 2018. Isolation and characterisation of antioxidative peptides from bromelain-hydrolysed brown rice protein by proteomic technique. *Process Biochemistry*, [e-journal] 70, pp.179–187.

Sheng, J. et al., 2019. Antioxidative Effects and Mechanism Study of Bioactive Peptides from Defatted Walnut (Juglans regia L.) Meal Hydrolysate. *Journal of Agricultural and Food Chemistry*, [e-journal] 67(12), pp.3305–3312. https://doi.org/10.1021/ACS.JAFC.8B05722.

Silva-Sánchez, C. et al., 2008. Bioactive Peptides in Amaranth (Amaranthus hypochondriacus) Seed. *Journal of Agricultural and Food Chemistry*, [e-journal] 56(4), pp.1233–1240. https://doi.org/10.1021/JF072911Z.

Singh, B.P., Vij, S. and Hati, S., 2014. Functional significance of bioactive peptides derived from soybean. *Peptides*, [e-journal] 54, pp.171–179.

Siow, H.L. and Gan, C.Y., 2016. Extraction, identification, and structure–activity relationship of antioxidative and α-amylase inhibitory peptides from cumin seeds (Cuminum cyminum). *Journal of Functional Foods*, [e-journal] 22, pp.1–12.

Siow, H.L. and Gan, C.Y., 2013. Extraction of antioxidative and antihypertensive bioactive peptides from Parkia speciosa seeds. *Food Chemistry*, [e-journal] 141(4), pp.3435–3442.

Sonklin, C., Alashi, A.M., Laohakunjit, N. and Aluko, R.E., 2021. Functional characterization of mung bean meal protein-derived antioxidant peptides. *Molecules*, [e-journal] 26(6), p.1515. https://doi.org/10.3390/molecules26061515.

Suetsuna, K. and Chen, J.R., 2002. Isolation and Characterization of Peptides with Antioxidant Activity Derived from Wheat Gluten. *Food Science and Technology Research*, [e-journal] 8(3), pp.227–230.

Torres-Fuentes, C. et al., 2015. Identification and characterization of antioxidant peptides from chickpea protein hydrolysates. *Food Chemistry*, [e-journal] 180, pp.194–202.

Vásquez-Villanueva, R., Marina, M.L. and García, M.C., 2016. Identification by hydrophilic interaction and reversed-phase liquid chromatography–tandem mass spectrometry of peptides with antioxidant capacity in food residues. *Journal of Chromatography A*, [e-journal] 1428, pp.185–192.

Wang, L., Ma, M., Yu, Z. and Du, S. kui, 2021. Preparation and identification of antioxidant peptides from cottonseed proteins. *Food Chemistry*, [e-journal] 352, p.129399.

Wang, M. et al., 2019. In vitro and in silico antioxidant activity of novel peptides prepared from paeonia ostii 'feng dan' hydrolysate. *Antioxidants*, [e-journal] 8(10), p.433. https://doi.org/10.3390/antiox8100433.

Wang, X. et al., 2017. A novel antioxidant and ACE inhibitory peptide from rice bran protein: Biochemical characterization and molecular docking study. *LWT*, [e-journal] 75, pp.93–99.

Wang, X.J. et al., 2014. Purification and evaluation of a novel antioxidant peptide from corn protein hydrolysate. *Process Biochemistry*, [e-journal] 49(9), pp.1562–1569.

Wei, C., Su, D.N., Mee, R.K. and Sok, D.E., 2007. Rice albumin N-terminal (Asp-His-His-Gln) prevents against copper ion-catalyzed oxidations. *Journal of Agricultural and Food Chemistry*, [e-journal] 55(6), pp.2149–2154. https://doi.org/10.1021/jf062387g.

Wen, C. et al., 2020. Purification and identification of novel antioxidant peptides from watermelon seed protein hydrolysates and their cytoprotective effects on H2O2-induced oxidative stress. *Food Chemistry*, [e-journal] 327, p.127059.

Wen, C. et al., 2021. Study on the structure–activity relationship of watermelon seed antioxidant peptides by using molecular simulations. *Food Chemistry*, [e-journal] 364, p.130432.

Wu, C. et al., 2013. Purification and identification of novel antioxidant peptides from enzymatic hydrolysate of ginkgo biloba seed proteins. *Food Science and Technology Research*, [e-journal] 19(6), pp.1029–1035.

Xia, Y., Bamdad, F., Gänzle, M. and Chen, L., 2012. Fractionation and characterization of antioxidant peptides derived from barley glutelin by enzymatic hydrolysis. *Food Chemistry*, [e-journal] 134(3), pp.1509–1518.

Yan, Q.J. et al., 2015. Isolation, identification and synthesis of four novel antioxidant peptides from rice residue protein hydrolyzed by multiple proteases. *Food Chemistry*, [e-journal] 179, pp.290–295.

Yang, Juanjuan et al., 2018. Purification and identification of two novel antioxidant peptides from perilla (Perilla frutescens L. Britton) seed protein hydrolysates. *PLOS ONE*, [e-journal] 13(7), p.e0200021. https://doi.org/10.1371/JOURNAL.PONE.0200021.

Yang, R. et al., 2017. Identification of novel peptides from 3 to 10 kDa pine nut (Pinus koraiensis) meal protein, with an exploration of the relationship between their antioxidant activities and secondary structure. *Food Chemistry*, [e-journal] 219, pp.311–320.

Yokomizo, A., Takenaka, Y. and Takenaka, T., 2002. Antioxidative Activity of Peptides Prepared from Okara Protein. *Food Science and Technology Research*, [e-journal] 8(4), pp.357–359.

Yu, Y.P. et al., 2021. Peptidomic analysis of low molecular weight antioxidative peptides prepared by lotus (Nelumbo nucifera Gaertn.) seed protein hydrolysates. *LWT*, [e-journal] 144, p.111138.

Zarei, M. et al., 2014. Identification and characterization of papain-generated antioxidant peptides from palm kernel cake proteins. *Food Research International*, [e-journal] 62, pp.726–734.

Zhang, F. et al., 2019. Purification and identification of an antioxidative peptide from peony (Paeonia suffruticosa Andr.) seed dreg. *Food Chemistry*, [e-journal] 285, pp.266–274.

Zhang, J. et al., 2009. Antioxidant activities of the rice endosperm protein hydrolysate: identification of the active peptide. *European Food Research and Technology 2009 229:4*, [e-journal] 229(4), pp.709–719. https://doi.org/10.1007/S00217-009-1103-3.

Zhang, J. et al., 2010. Isolation and identification of antioxidative peptides from rice endosperm protein enzymatic hydrolysate by consecutive chromatography and MALDI-TOF/TOF MS/MS. *Food Chemistry*, [e-journal] 119(1), pp.226–234.

Zhang, S. et al., 2019. Preparation, identification, and activity evaluation of antioxidant peptides from protein hydrolysate of corn germ meal. *Journal of Food Processing and Preservation*, [e-journal] 43(10), p.e14160. https://doi.org/10.1111/jfpp.14160.

Zhang, T., Li, Y., Miao, M. and Jiang, B., 2011. Purification and characterisation of a new antioxidant peptide from chickpea (Cicer arietium L.) protein hydrolysates. *Food Chemistry*, [e-journal] 128(1), pp.28–33.

Zhao, Y., Zhao, Q. and Lu, Q., 2020. Purification, structural analysis, and stability of antioxidant peptides from purple wheat bran. *BMC Chemistry*, [e-journal] 14(1), pp.1–12. https://doi.org/10.1186/s13065-020-00708-z.

Zheng, L. et al., 2012. Isolation and Characterization of an Oxygen Radical Absorbance Activity Peptide from Defatted Peanut Meal Hydrolysate and Its Antioxidant Properties. *Journal of Agricultural and Food Chemistry*, [e-journal] 60(21), pp.5431–5437. https://doi.org/10.1021/JF3017173.

Zheng, X. et al., 2006. Production of hydrolysate with antioxidative activity by enzymatic hydrolysis of extruded corn gluten. *Applied Microbiology and Biotechnology 2006 73:4*, [e-journal] 73(4), pp.763–770. https://doi.org/10.1007/S00253-006-0537-9.

Zheng, Y., Li, Y. and Li, G., 2019. ACE-inhibitory and antioxidant peptides from coconut cake albumin hydrolysates: Purification, identification and synthesis. *RSC Advances*, [e-journal] 9(11), pp.5925–5936. https://doi.org/10.1039/c8ra10269d.

Zheng, Y., Li, Y. and Zhang, Y., 2017. Purification and identification of antioxidative peptides of palm kernel expeller glutelin-1 hydrolysates. *RSC Advances*, [e-journal] 7(85), pp.54196–54202. https://doi.org/10.1039/c7ra11657h.

Zou, Z. et al., 2020. Antihypertensive and antioxidant activities of enzymatic wheat bran protein hydrolysates. *Journal of Food Biochemistry*, [e-journal] 44(1), p.e13090. https://doi.org/10.1111/JFBC.13090.

Table S2: Search words used in Scopus to compile seed-derived antioxidant peptides.

No.	Search word
1	"antioxidant peptides" OR "antioxidative peptides" AND "seeds"
2	"antioxidant peptide" OR "antioxidative peptide" AND "seed"
3	"antioxidant peptide" OR "antioxidative peptide" AND "cereal"
4	"antioxidant peptide" OR "antioxidative peptide" AND "legume"
5	"antioxidant peptide" OR "antioxidative peptide" AND "pea"
6	"antioxidant peptide" OR "antioxidative peptide" AND "nut"
7	"antioxidant peptide" OR "antioxidative peptide" AND "cocoa bean"
8	"antioxidant peptide" OR "antioxidative peptide" AND "coffee bean"
9	"antioxidant peptide" OR "antioxidative peptide" AND "bean"
10	"antioxidant peptide" OR "antioxidative peptide" AND "durian seed"
11	"antioxidant peptide" OR "antioxidative peptide" AND "mustard seed"
12	"antioxidant peptide" OR "antioxidative peptide" AND "poppy seed"
13	"antioxidant peptide" OR "antioxidative peptide" AND "pomegranate seed"
14	"antioxidant peptide" OR "antioxidative peptide" AND "chestnut"
15	"antioxidant peptide" OR "antioxidative peptide" AND "peanut"
16	"antioxidant peptide" OR "antioxidative peptide" AND "hazelnut"
17	"antioxidant peptide" OR "antioxidative peptide" AND "palm kernel"
18	"antioxidant peptide" OR "antioxidative peptide" AND "coconut kernel"

Table S3: Coordinates of box center and box size for different targets in molecular docking, and RMSD values.

Target	Box center			Box size (Å)			RMSD value
	x	y	z	x	y	z	
$p47^{phox}$	-	-	-	-	-	-	1.54
Keap1	5	7	2	15	15	15	1.41
MPO	-25	-45	43	25	25	25	1.07
XO	29	21	15	25	25	25	1.82

Table S4: Docking scores for peptides that were experimentally demonstrated to inhibit p47^{phox}-p22^{phox} interaction and NADPH oxidase, in comparison with p22^{phox}

Peptide	Docking score
p22^{phox}-derived proline-rich peptide (GPLGSKQPPSNPPPRPPAEARKKPS)[1]	-309.862
RRSSIRNAHSIHQRSRKRLS[2]	-268.079
ISNSESGPRGVHFIFNKENF[3]	-266.627
RSRKRLSQDAYRRNSVRFLQQR[2]	-257.842
AGGPPGGPQVNPIPVTDEVV[3]	-202.725

HPEPDOCK access date: 1/12/2021

[1] Ogura, K.; Nobuhisa, I.; Yuzawa, S.; Takeya, R.; Torikai, S.; Saikawa, K.; Sumimoto, H.; Inagaki, F. NMR solution structure of the tandem Src homology 3 domains of p47^{phox} complexed with a p22^{phox}-derived proline-rich peptide. Journal of Biological Chemistry 2006, 281, 3660-3668, doi:10.1074/jbc.M505193200.

[2] Huang, J.; Kleinberg, M.E. Activation of the phagocyte NADPH oxidase protein p47^{phox}: Phosphorylation controls SH3 domain-dependent binding to p22^{phox}*. Journal of Biological Chemistry 1999, 274, 19731-19737, doi:10.1074/jbc.274.28.19731.

[3] Nakanishi, A.; Imajoh-Ohmi, S.; Fujinawa, T.; Kikuchi, H.; Kanegasaki, S. Direct evidence for interaction between COOH-terminal regions of cytochrome b_{558} subunits and cytosolic 47-kDa protein during activation of an O($_{2}^{-}$)-generating system in neutrophils. Journal of Biological Chemistry 1992, 267, 19072-19074.

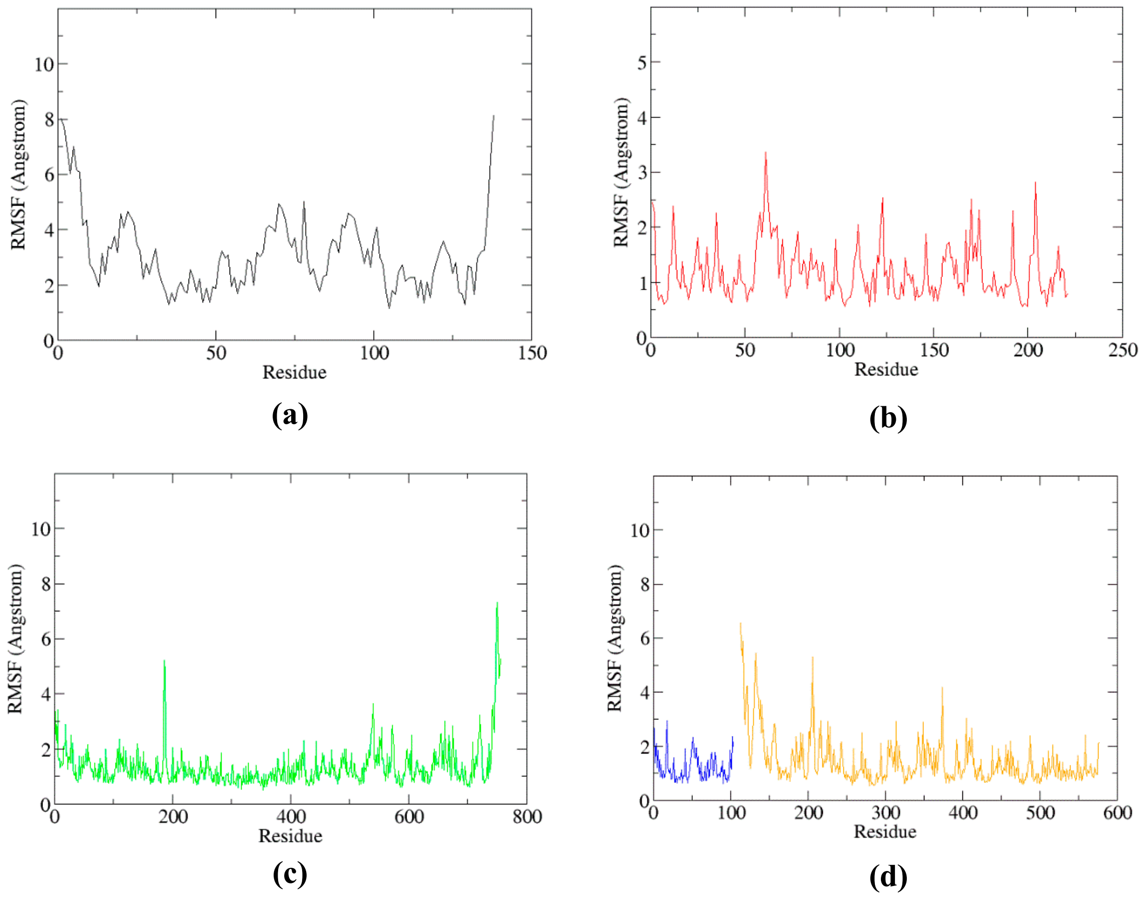

Figure S1: RMSF plots for **(a)** PSYLNTPLL-p47^{phox}, **(b)** LYSPH-Keap1, **(c)** LYSPH-XO, and **(d)** LYSPH-MPO.

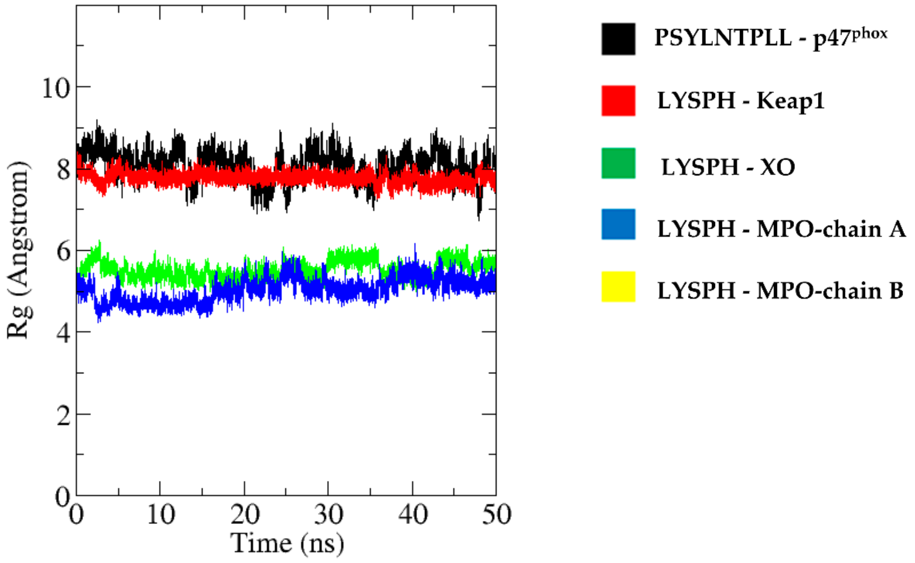

Figure S2: Gyration (Rg) plots of each complex.
